
JOURNAL INFORMATION
==============================
NLM Title Abbreviation: Insights Imaging
Journal ID: Insights Imaging
NLM Journal ID: 101532453

Insights into Imaging

EISSN: 1869-4101
Publisher: Springer

ARTICLE INFORMATION
==============================
PMCID: PMC10229496
PMID: 37249670
DOI: 10.1186/s13244-023-01434-5
Article ID: 1434
Article version: 1
Subjects: Meeting Abstracts

ESGAR 2023 Book of Abstracts

Electronic publication date: 2023 May 30
Publication date: 2023 May
Volume: 14
Issue: Suppl 2
Issue sponsor: Publication costs were funded by the European Society of Gastrointestinal and Abdominal Radiology.
Electronic Location ID: 101
Copyright: © The Author(s) 2023
License: Open AccessThis article is licensed under a Creative Commons Attribution 4.0 International License, which permits use, sharing, adaptation, distribution and reproduction in any medium or format, as long as you give appropriate credit to the original author(s) and the source, provide a link to the Creative Commons licence, and indicate if changes were made. The images or other third party material in this article are included in the article's Creative Commons licence, unless indicated otherwise in a credit line to the material. If material is not included in the article's Creative Commons licence and your intended use is not permitted by statutory regulation or exceeds the permitted use, you will need to obtain permission directly from the copyright holder. To view a copy of this licence, visit http://creativecommons.org/licenses/by/4.0/.
License URL: https://creativecommons.org/licenses/by/4.0/

Conference name: 34th Annual Meeting and Postgraduate Course ESGAR 2023
Conference location: Valencia, Spain
Conference date: 13-16 June 2023
issue-copyright-statement: © European Society of Radiology (ESR) 2023

==============================

European Society ESGAR Gastrointestinal and Abdominal Radiology

### Insights into Imaging

Critical Science and Reviews advancing the practice of radiology

ESGAR 2023 Book of Abstracts / Volume 14 / Supplement 2 / May 2023

ESGAR 2023 / June 13 - 16 / Valencia, Spain
34th Annual Meeting and Postgraduate Course

ESR European Society of Radiology

European Society ESGAR Gastrointestinal and Abdominal Radiology

### ESGAR

### Annual Meeting and Postgraduate Course

### 2023

### June 13-16

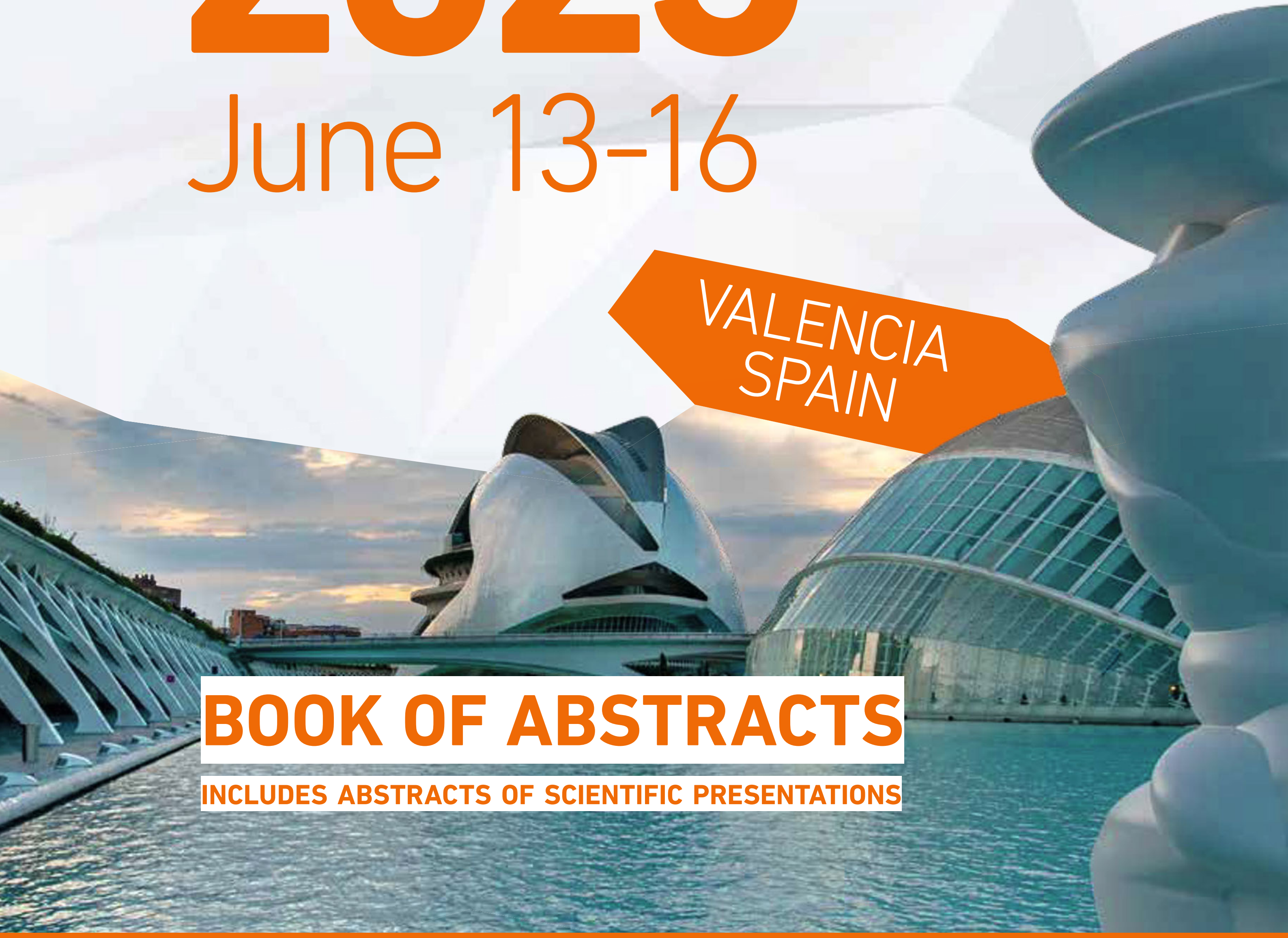

### BOOK OF ABSTRACTS

INCLUDES ABSTRACTS OF SCIENTIFIC PRESENTATIONS

*IMPORTANT ADDRESSES*

**ORGANISING SECRETARIAT**
**Central ESGAR Office**
Esslinggasse 2/3
AT – 1010 Vienna
Phone: +43 1 535 89 27
E-Mail: office@esgar.org www.esgar.org

### CME CREDITS

The "ESGAR European Society of Gastrointestinal and Abdominal Radiology" (or) "ESGAR 2023 – 34th ANNUAL MEETING AND POSTGRADUATE COURSE" is accredited by the European Accreditation Council for Continuing Medical Education (EACCME) to provide the following CME activity for medical specialists. The EACCME is an institution of the European Union of Medical Specialists (UEMS), www.uems.net.

The 34th Annual Meeting and Postgraduate Course ESGAR 2023, Valencia, Spain, 13/06/2023-16/06/2023 has been accredited by the European Accreditation Council for Continuing Medical Education (EACCME®) with **25** European CME credits (ECMEC®s). Each medical specialist should claim only those hours of credit that he/she actually spent in the educational activity.

### CONTRIBUTING SOCIETIES

European Society of Urogenital Radiology

ASAR – Asian Society of Abdominal Radiology – Since 2007

### SPONSORS

ESGAR wishes to gratefully acknowledge the support of its Corporate Members:

BAYER

BRACCO – LIFE FROM INSIDE

GE

Guerbet

The Final Programme of ESGAR 2023 is available on the ESGAR Website www.esgar.org

Insights Imaging (2023) 14 (Suppl 2): 101
https://doi.org/10.1186/s13244-023-01434-5
Published: 30 May 2023

### *TABLE OF CONTENTS*

| | | |
|---|---|---|
| Scientific Sessions, Wednesday, June 14 | (SS 1.1 – SS 1.10) | 8-10 |
| Scientific Sessions, Wednesday, June 14 | (SS 2.1 – SS 2.9) | 10-12 |
| Scientific Sessions, Wednesday, June 14 | (SS 3.1 – SS 3.10) | 13-15 |
| Scientific Sessions, Wednesday, June 14 | (SS 4.1 – SS 4.10) | 15-18 |
| Scientific Sessions, Thursday, June 15 | (SS 5.1 – SS 5.10) | 19-21 |
| Scientific Sessions, Thursday, June 15 | (SS 6.1 – SS 6.11) | 21-24 |
| Scientific Sessions, Thursday, June 15 | (SS 7.1 – SS 7.10) | 25-27 |
| Scientific Sessions, Thursday, June 15 | (SS 8.1 – SS 8.10) | 27-30 |
| Scientific Sessions, Thursday, June 15 | (SS 9.1 – SS 9.10) | 30-32 |
| Scientific Sessions, Friday, June 16 | (SS 10.1 – SS 10.10) | 33-35 |
| Scientific Sessions, Friday, June 16 | (SS 11.1 – SS 11.10) | 35-37 |
| Scientific Sessions, Friday, June 16 | (SS 12.1 – SS 12.10) | 38-40 |
| AUTHORS’ INDEX | | 42-45 |

### ESGAR EXECUTIVE COMMITTEE

PRESIDENT
*A. Laghi, Rome/IT*

PRESIDENT-ELECT
*M. Zins, Paris/FR*

VICE PRESIDENT
*J. Stoker, Amsterdam/NL*

SECRETARY/TREASURER
*V. Vilgrain, Clichy/FR*

PROGRAMME COMMITTEE CHAIR
*J. Stoker, Amsterdam/NL*

EDUCATION COMMITTEE CHAIR
*C.J. Zech, Basel/CH*

MEMBERSHIP AND COMMUNICATION COMMITTEE CHAIR
*G. Brancatelli, Palermo/IT*

RESEARCH COMMITTEE CHAIR
*S.A. Taylor, London/UK*

WORKSHOP COMMITTEE CHAIR
*M.A. Bali, Brussels/BE*

MEETING PRESIDENT 2023
*A. Torregrosa Andrés, Valencia/ES*

MEETING PRESIDENT 2024
*L. Blomqvist, Stockholm/SE*

MEETING PRESIDENT 2025
*J. Stoker, Amsterdam/NL*

MEMBERS AT LARGE
*S. Gourtsoyianni, Athens/GR*
*M. Karcaaltincaba, Ankara/TR*
*N. Kartalis, Stockholm/SE*
*M. Radzina, Riga/LV*
*D. Tolan, Leeds/UK*

ESGAR EXECUTIVE DIRECTOR
*B. Lindlbauer, Vienna/AT*

### ESGAR 2023 MEETING PRESIDENT

*Asunción Torregrosa Andrés*
*Area Clínica de Imagen Médica*
*Hospital Universitari i Politècnic La Fe*
*Avinguda de Fernando Abril Martorell, 106*
*46026 Valencia, Spain*

### ESGAR 2023 PROGRAMME COMMITTEE

CHAIRMAN
*J. Stoker, Amsterdam/NL*

MEMBERS
*M.A. Bali, Brussels/BE*
*L. Blomqvist, Stockholm/SE*
*G. Brancatelli, Palermo/IT*
*L. Curvo-Semedo, Coimbra/PT*
*S. Gourtsoyianni, Athens/GR*
*M. Karcaaltincaba, Ankara/TR*
*N. Kartalis, Stockholm/SE*
*A. Laghi, Rome/IT*
*M. Radzina, Riga/LV*
*S.A. Taylor, London/UK*
*D. Tolan, Leeds/UK*
*A. Torregrosa Andrés, Valencia/ES*
*V. Vilgrain, Clichy/FR*
*C.J. Zech, Basel/CH*
*M. Zins, Paris/FR*

### ESGAR 2023 LOCAL ORGANISING COMMITTEE

*A. Arjonilla López, Madrid/ES*
*A. Batista Doménech, Valencia/ES*
*L. Martí-Bonmatí, Valencia/ES*
*J.R. Olalla Munoz, Murcia/ES*
*A. Pérez-Girbés, Valencia/ES*
*T. Ripollés González, Valencia/ES*
*P. Rudenko, Valencia/ES*
*G. Tardáguila de la Fuente, Vigo/ES*

*O. Akhan, Ankara/TR*
*M.A. Bali, Brussels/BE*
*T.V. Bartolotta, Palermo/IT*
*A. Ba-Ssalamah, Vienna/AT*
*R.G.H. Beets-Tan, Amsterdam/NL*
*E. Biscaldi, Genoa/IT*
*A. Blachar, Tel Aviv/IL*
*L. Blomqvist, Stockholm/SE*
*I. Boulay Coletta, Paris/FR*
*G. Brancatelli, Palermo/IT*
*R. Cannella, Palermo/IT*
*F. Caseiro-Alves, Coimbra/PT*
*L. Crocetti, Pisa/IT*
*L. Curvo-Semedo, Coimbra/PT*
*G. D'Assignies, La Roche-sur-Yon/FR*
*K. De Paepe, Cambridge/UK*
*M. Dioguardi Burgio, Villejuif/FR*
*A. Dohan, Paris/FR*
*R.F. Dondelinger, Liège/BE*
*M. D'Onofrio, Verona/IT*
*H. Fenlon, Dublin/IE*
*A. Furlan, Pittsburgh, PA/US*
*V. Goh, London/UK*
*M. Gollub, New York, NY/US*
*S. Gourtsoyianni, Athens/GR*
*J.A. Guthrie, Leeds/UK*
*T. Helmberger, Munich/DE*
*F. Iafrate, Rome/IT*
*D. Ippolito, Monza/IT*
*S. Jackson, Plymouth/UK*
*M. Karcaaltincaba, Ankara/TR*
*A. Laghi, Rome/IT*
*D. Lambregts, Amsterdam/NL*
*M. Laniado, Dresden/DE*
*J.M. Lee, Seoul/KR*
*O. Lucidarme, Paris/FR*
*M. Maas, Amsterdam/NL*
*A.J. Madureira, Porto/PT*
*T. Mang, Vienna/AT*
*V. Maniatis, Sønderborg/DK*
*L. Martí-Bonmatí, Valencia/ES*
*C. Matos, Lisbon/PT*
*Y. Menu, Paris/FR*
*G. Morana, Treviso/IT*
*S. Mulé, Créteil/FR*
*P. Paolantonio, Rome/IT*
*N. Papanikolaou, Lisbon/PT*
*A. Plumb, Esher/UK*
*S. Pötter-Lang, Vienna/AT*
*R. Pozzi Mucelli, Verona/IT*
*P. Prassopoulos, Thessaloniki/GR*
*M. Ronot, Clichy/FR*
*W. Schima, Vienna/AT*
*S. Schmidt Kobbe, Lausanne/CH*
*A. Schreyer, Regensburg/DE*
*O. Seror, Bondy/FR*
*S. Skehan, Dublin/IE*
*E. Soloff, Seattle/US*
*M. Staunton, Cork/IE*
*S. Stojanovic, Novi Sad/RS*
*J. Stoker, Amsterdam/NL*
*C. Stroszczynski, Regensburg/DE*
*A. Taibbi, Palermo/IT*
*B. Taouli, New York, NY/US*
*J.P. Tasu, Poitiers/FR*
*S.A. Taylor, London/UK*
*D. Tolan, Leeds/UK*
*C. Triantopoulou, Athens/GR*
*V. Valek, Brno-Bohunice/CZ*
*F. Vernuccio, Palermo/IT*
*V. Vilgrain, Clichy/FR*
*M.-P. Vullierme, Clichy/FR*
*M. Wagner, Paris/FR*
*G. Zamboni, Verona/IT*
*C.J. Zech, Basel/CH*
*M. Zins, Paris/FR*

**09:00 - 10:30** **Room 1**

*Scientific Session SS 1*
### Colorectal cancer

### SS 1.1
### Incidence of advanced adenoma and colorectal cancer after screening CTC in the SAVE randomised trial
L. Sali, D. Puliti, M. Mascalchi, B. Mallardi, M. Zappa, G. Gorini, P. Mantellini; *Firenze/IT*

**Purpose:** The SAVE randomised trial compared single CTC versus three biennial faecal immunochemical test (FIT) rounds for population screening of colorectal cancer (CRC). We evaluated herein the incidence of advanced adenoma and CRC in subjects of the CTC and FIT groups after the completion of the trial.
**Material and Methods:** From 2017 to 2020, 1219 subjects in the CTC group and 5841 in the FIT group with a negative initial screening test were invited to two biennial FIT. Subjects positive at one of the subsequent FITs were referred to optical colonoscopy. The outcome was the number of advanced adenoma and CRC over the number of subjects with negative initial screening test.
**Results:** The average time interval between the initial screening test and the last subsequent FIT was 6.1 years in the CTC group and 3.5 years in the FIT group. Participation to subsequent FIT in the CTC group was 80% (976/1219) at first and 54% (661/1219) at second round, whereas in the FIT group, it was 72% (4204/5841) at first and 45% (2651/5841) at second round. Overall in the two subsequent FIT rounds, 4 advanced adenomas and no CRC were detected in the CTC group, whereas 39 advanced adenomas and 8 cancers were found in the FIT group (p=0.06).
**Conclusion:** We observed a trend in reduced incidence of advanced adenoma and CRC in subjects screened with single CTC as compared to those screened with three FIT rounds.

### SS 1.2
### CTC in routine UK clinical practice: do services meet British Society of Gastrointestinal and Abdominal Radiologists standards?
Y. Wu[1], L. Lee[1], J. Stephenson[2], K. Foley[3], D. Tolan[4]; *[1]Leicester/GB, [2]Market Harborough/GB, [3]Llantrisant/GB, [4]Leeds/GB*

**Purpose:** Quality evaluation of National Health Service (NHS) CTC services against British Society of Gastrointestinal and Abdominal Radiologists (BSGAR) standards published in 2021.
**Material and Methods:** This is a national, multi-centre, retrospective audit. Radiology services were invited to submit CTC examination data from 2019, which were evaluated against minimum and aspirational standards for image quality, polyp identification rate (PIR), positive predictive value (PPV), and technique including additional acquisition rate, radiation dose and same-day CT staging.
**Results:** 17 centres participated, serving over 8.4 million patients. Data from 22,559 CTC were included (mean=1327 per centre, range 168–2970). 96% CTC were symptomatic and 4% screening examinations. 59% (10/17) routinely used CTC reporting pro forma and 53% (9/17) used reporting codes. Considerable variation between 120 reporters existed (mean 188, range 2–886) with only 47/120 (39.6%) exceeding the aspirational target of 175 per annum. PIR ranged from 7.7 to 24%, with only 10/17 (59%) of centres exceeding the 13% minimum standard and 8/17 (47%) exceeding the 16% aspirational standard. PPV ranged from 61 to 100% with 13/16 (81.3%) centres exceeding the 80% minimum standard and 7/16 (43.8%) exceeding the 90% aspiration standard. Only 3/17 (17.6%) centres exceeded aspirational standards for both PIR and PPV. Additional acquisitions over dual position ranged 0–25%; 53% (9/17) of centres performed additional scans in <5% of CTC, under minimum standards.
**Conclusion:** While some NHS CTC services demonstrated excellence, either meeting or exceeding minimum standards, many UK services did not. Comprehensive audit and training are required to ensure services improve sufficiently to meet necessary quality assurance standards.

### SS 1.3
### Developing consensus guidance on CTC in colorectal cancer pathways: the British Society of Gastrointestinal and Abdominal Radiologists experience
D. Tolan[1], S. Taylor[2], A. Plumb[2]; *[1]Leeds/GB, [2]London/GB*

**Purpose:** CTC is an essential diagnostic modality in the UK for the management of patients with symptoms of colorectal cancer (CRC). Multiprofessional recommendations are needed to develop referral pathways, quality assurance, leadership, workforce and infrastructure to support future service delivery.
**Material and Methods:** A small leadership group presented themes for discussion to a group of 19 recognised experts representing radiology, radiography, gastroenterology and colorectal surgery in England. Statements created from the discussion were developed with a modified Delphi approach until consensus was reached.
**Results:** Experts concluded that CTC is an appropriate first line test for the exclusion of CRC and large polyps in patients with symptoms suggestive of bowel cancer. It is also indicated after incomplete colonoscopy for new symptoms suggesting CRC or colorectal surveillance; abdominal symptoms requiring colonic and extracolonic evaluation; where comorbidities increase risk of colonoscopy for new symptoms or colorectal surveillance; previous failed colonoscopy where a repeat is unlikely to succeed; or patient choice in symptomatic patients after counselling. CTC should be generally avoided in patients too unfit for the CTC procedure; symptoms or surveillance of IBD/microscopic colitis; anal canal symptoms; those under 40 years old; and after recent abdominal CT imaging where colonoscopy could be performed. Services should routinely use structured report templates and produce quality assurance reports every 3 years using British Society of Gastrointestinal and Abdominal Radiologists guidelines. Leadership, workforce and infrastructure needs were also defined.
**Conclusion:** Experts have now defined the role for CTC in CRC pathways and the quality assurance and resources required for services.

### SS 1.4
### Diagnostic accuracy of CT in preoperative staging of colon cancer patients: the impact of readers' experience
D. Ippolito[1], C. Maino[2], M. Ragusi[3], T. Giandola[1], C. Talei Franzesi[1], R. Corso[1]; *[1]Monza/IT, [2]Milan/IT, [3]Monza/IT*

**Purpose:** To assess agreement and diagnostic accuracy between different radiologists in the detection of specific features related to the T and N stage in colon cancer patients.
**Material and Methods:** We enrolled 75 patients who underwent preoperative contrast-enhanced computed tomography (CECT) and surgical resection for colon cancer. Pathological data were used as standard of reference. Images were reviewed blindly by 3 radiologists with different experience in abdominal imaging: the most experienced one (R1), an abdominal radiologist (R2) and a general radiologist (R3). Radiologists were asked to localize tumor and evaluate the following parameters: location, number of lesions, parietal thickening, tumor bulging, perivisceral fat stranding, lateroconal fascia invasion, vessel enlargement (> 2 mm), and node appearance (shape, enhancement, presence of clusters). Reliability analysis was assessed with κ statistic or interclass correlation coefficient.
**Results:** Tumor location, number of lesions and circumferential thickening showed an almost perfect concordance (κ=0.853, κ=0.800 and κ=0.671, respectively). T staging demonstrated an overall moderate agreement (κ=0.531), which was only considered fair for the T3 group (κ=0.402). Overall agreement was moderate in the evaluation of bulging (κ=0.478), perivisceral inhomogeneity (κ=0.490), and lateroconal fascia invasion (κ=0.436). Moderate to substantial agreement existed for nodal enhancement (κ=0.431), shape (κ=0.484), short axis (ICC=0.732) and intranodal necrosis (κ=0.606), while the evaluation of nodes' cluster was fair (κ=0.358). When comparing CT findings between R1 and R3 and between R2 and R3, all parameters showed reduced reliability for nodes (κ=0.287–0.477) and lesion characteristics (κ=0.288–0.545), except for tumor location and tumor focality (κ=0.710–0.825).
**Conclusion:** Despite CT represents a reliable diagnostic tool for the assessment of colon cancer patients, reader's experience remains one of the most important factors associated with correct evaluation of specific features in T and N staging.

### SS 1.5

### Structured templates in reporting CT staging on colon cancer

R.D. Andersen, M. Pedersen, L. Hesseldal, S. Rafaelsen; *Vejle/DK*

**Purpose:** To evaluate the use of CT-free text reports and structured report templates, when it comes to staging CT imaging of colon cancer.
**Material and Methods:** A multiple-choice questionnaire survey was conducted in the span of 4 weeks. Online questionnaire software was used, and a link was shared to medical doctors with help from the Danish Colorectal Cancer Group (DCCG) and from a social media radiologist group.
**Results:** Clinicians preferred the template style (95%), whereas the support for template reports was less among the radiologist (76%). All female responders preferred the template style; this was only true for 84% of the male responders. Furthermore, the survey showed a slightly deficient level of national CT-reporting quality, only seven out of thirteen questions and sub-questions, concerning CT report quality, achieved an approval rate of more than 85%. The colorectal cancer multi-disciplinary team consultants who always or usually work with template style reporting of CT scans of colon cancer tend to be more satisfied with the quality and content of the reports, compared to those who rarely use or read template reports.
**Conclusion:** The following indicators were insufficiently reported: tumor invasion growth, number of hepatic metastasis, segment location of hepatic metastasis and retroperitoneal lymph node involvement. In the template report group, nearly all participants found relevant information easily accessible.

### SS 1.6

### Diagnostic performance of contrast-enhanced CT in loco-regional staging and evaluation of retroperitoneal surgical margin of colon cancer patients

C. Maino[1], T. Giandola[2], M. Ragusi[1], C. Talei Franzesi[1], R. Corso[1], D. Ippolito[2]; *[1]Milan/IT, [2]Monza/IT*

**Purpose:** To evaluate the diagnostic accuracy of preoperative contrast-enhanced CT (CECT) in local staging of colon cancer compared with histopathological data as standard of reference.
**Material and Methods:** We retrospectively enrolled 75 patients who underwent CECT before surgical resection. Images were reviewed blindly by a radiologist with 15 years' experience in abdominal imaging to localize and stage the tumor. Each lesion was classified into T2, T3, and T4. The following parameters were collected: tumor bulging, fat stranding, lateroconal fascia invasion, enlarged vessels, the axial short diameter of the biggest node, shape, enhancement pattern, intranodal necrosis and nodes' cluster. Diagnostic values were computed and compared using contingency tables and ROC curves, respectively.
**Results:** The most frequent tumor location was the right colon (55%), followed by sigmoid (26%), left colon (14%) and transverse (5%). Sensitivity was highest for T3 lesions, reaching a value of 69%, whereas specificity was highest for T2 lesions (90%), and T4 (82.6%). The PPV was intermediate for all lesion categories, reaching a maximum value of 50%, while the NPV was acceptable for all lesion categories, with a maximum of 84.4% for T2 lesions. Overall, diagnostic accuracy reached optimal values for T2 [92% (85.2–99.1)] and T4 [76% (59.5–93.4)] lesions, while modest for T3 lesions [69% (55.3–82.9)]. The lateroconal fascia invasion and enlarged vessels resulted as independent predictor factors (OR=3.292 and OR=2.651) for T staging, while nodes' cluster and dimension as independent predictors factors of N staging (OR=3.798 and OR=1.083).
**Conclusion:** CT is a useful tool in staging T2 and T4 lesions.

### SS 1.7

### Proximal location of the largest lymph node on CT is associated with lymph node metastases in colon cancer

J. Platt, J. Seligmann, D. Tolan; *Leeds/GB*

**Purpose:** The accuracy of CT lymph node (N) staging in colon cancer (CC) is poor. Incorrect N stage may risk inappropriate systemic or surgical intervention, necessitating the need for novel strategies to identify nodal metastases. Here, we investigated whether characteristics of the largest node and the distribution of enlarged nodes within the primary tumour drainage territory are associated with the presence of nodal metastases. We also explored whether radiological features of the largest node differ between mismatch repair proficient (pMMR) and mismatch repair deficient (dMMR) CC.
**Material and Methods:** Through retrospective analysis of pre-operative CT scans from March 2019 to July 2020, all enlarged nodes (>5mm in long axis) on axial images were assessed for location and diameter (short and long). For the largest node, short-to-long axis ratio, average attenuation and variation in attenuation were recorded. Features were compared between pMMR and dMMR CC.
**Results:** In 92 patients with newly diagnosed CC, proximal location of the largest node was associated with having nodal metastases (P=0.038 for 'proximal' vs. 'distal'). Borderline significance was observed when classified as 'pericolic', 'intermediate' or 'main' (P=0.062). No other features were associated with the presence of nodal metastases. Short-to-long axis ratio and average attenuation of the largest node were significantly greater in dMMR CC than in pMMR CC (P<0.02 for both).
**Conclusion:** Proximal location of the largest node on CT is associated with nodal metastases in CC. Assessing node location may, therefore, represent a novel and effective strategy for improving CT staging accuracy in this population.

### SS 1.8

### Identifying pathways for recurrent disease in colorectal cancer patients after cytoreductive surgery and hyperthermic intraperitoneal chemotherapy

C. Rijsemus, E. Berardi, N. Kok, A. Aalbers, D. Lambregts, R. Beets-Tan, M. Lahaye; *Amsterdam/NL*

**Purpose:** Cytoreductive surgery (CRS) and hyperthermic intraperitoneal chemotherapy (HIPEC) patients are plagued by a high recurrence rate. No solutions have emerged in the literature to reduce recurrences. This study aims to identify pathways for recurrent disease after CRS–HIPEC in colorectal cancer (CRC) patients with peritoneal metastases (PM) by comparing MRIs before and after surgery.
**Material and Methods:** Included were CRC patients with recurrence after CRS–HIPEC who had an MRI at primary diagnosis (MRI1, before CRS–HIPEC) and at the time of recurrence (MRI2, after CRS–HIPEC). The MRIs were compared by 2 abdominal radiologists in consensus and the recurrent metastases were categorized as follows: (1) the same site as before surgery and (2) new PM, including abdominal wall metastases and (3) extra-peritoneal metastases. The recurrent metastases on MRI were compared with the histopathological findings of CRS–HIPEC. Reference standards for recurrent metastases were histopathology or follow-up imaging.
**Results:** Thirty-three patients were included, of which 74 recurrent metastases were depicted on MRI2. Thirty-seven (50%) metastases were visible in the same site as the pre-operative MRI1 and 37 (50%) metastases were found at sites where no metastases were seen on MRI1. In total, 18/74 metastases were found in the abdominal wall and 17/74 were extra-peritoneal metastases.
**Conclusion:** Recurrent metastases are often found in the same site as pre-operatively and in the abdominal wall. This knowledge suggests that 1. Surgeons must attempt to achieve a wider excision of metastases during CRS–HIPEC when possible. 2. Radiologists, when reporting follow-up scans, must evaluate in detail the abdominal wall and the sites where there were PM present before CRS–HIPEC.

### SS 1.9

**Correlation between MRI, surgical and pathological peritoneal cancer index for colorectal cancer patients undergoing cytoreductive surgery and hyperthermic intraperitoneal chemotherapy**

C. Rijsemus, N. Kok, A. Aalbers, R. Fijneman, P. Snaebjornsson, D. Lambregts, R. Beets-Tan, M. Lahaye; *Amsterdam/NL*

**Purpose:** Cytoreductive surgery and hyperthermic intraperitoneal chemotherapy (CRS–HIPEC) patients are selected depending on the extent and location of peritoneal metastases (PM) expressed using a surgical scoring system: the peritoneal cancer index (PCI). However, practice shows that lesions considered malignant by the surgeon during surgery and consequently resected may not contain viable tumor tissue at histology. DW-MRI is a less invasive way to determine the PCI and has a diagnostic performance comparable to surgery. Therefore, the aim of this study is to investigate if the PCI determined by DW-MRI (mriPCI) correlates better with the histological PCI (hPCI) than the surgical PCI (sPCI).
**Material and Methods:** Patients with PM from CRC who underwent CRS–HIPEC and an MRI pre-operatively were included. All initial MRI, surgical and pathological reports of patients were reviewed for the PCI.
**Results:** In this study, 62 patients were included. MRI outperformed the surgeon in 27/62 patients and the surgeon outperformed the MRI in 13/62 patients. The correlation coefficient between the sPCI and pPCI is 0.93, between mriPCI and pPCI 0.78 and between sPCI and mriPCI 0.79, all statistically significant.
**Conclusion:** All patients included in the study were correctly selected by MRI to undergo a CRS–HIPEC since all patients had a surgical PCI below 21. Although the human eye is (still) superior to the MRI when it comes to determining the actual extent of disease when pathology is the reference standard, it shows no clinical value. The agreement between the surgeon and the radiologist was excellent; MRI is a good alternative and less invasive way to select CRC patients for surgery.

### SS 1.10

**A supervised training of a deep learning 3D U-net method for automatic CT segmentation of colon cancers**

D. De Santis, M. Polici, D. Valanzuolo, M. Zerunian, D. Caruso, A. Laghi; *Rome/IT*

**Purpose:** To train a deep learning-based method for the automatic 3D colon cancer segmentation on preoperative CT scans.
**Material and Methods:** One hundred seven colon cancer patients at stage II–III were retrospectively enrolled from January 2015 to December 2020 at Sant'Andrea Hospital, Sapienza University of Rome. All patients had availability of preoperative CT scans and pathological diagnosis. One expert radiologist manually performed the segmentations of two regions of interest (ROIs), colon cancer and normal colon tracts, on CT portal venous phase using Slicer 3D software (v4.10.2). An expert physicist applied a supervised training of volumetric deep learning-based method (3D U-Net) to automatically segment the colon cancer. Manual segmentations were entered in supervised training as ground truth. Two training phases were performed, the first including only tumor ROIs and the second considering both tumor and normal colon tract ROIs. To assess the similarity between the predicted automatic segmentation and ground truth, the dice similarity coefficient (DSC) was calculated on a set of 16 CT scans. The time spent to perform colon cancer segmentation was also recorded and compared between automatic and manual segmentation with Mann–Whitney U test.
**Results:** The first phase of training showed a preliminary average DSC of 37% while the second phase of training reached a DSC of 47%. Automatic colon cancer segmentation was sensibly faster (183 ± 12 s) compared to manual segmentation (735 ± 32 s; P< 0.001).
**Conclusion:** Our results showed the adding value of normal colon tracts to improved network performance in tumor segmentation. Deep learning automatic segmentation could be an alternative to time-consuming manual segmentation.

**09:00 - 10:30** **Room 3**

*Scientific Session SS 2*

### Image guided therapy

### SS 2.1

**Necrosis volumetric assessment as a predictor for local tumor progression, post-radiofrequency ablation in HCC**

N.G. Iuhas, R.L. Dumitru, M.C. Grasu, M. Toma, I.G. Lupescu; *Bucharest/RO*

**Purpose:** Evaluating if necrosis volumetric assessment of pre-/post-radiofrequency ablation in HCC can be an accurate predictor of LTP (local tumor progression) and survival.
**Material and Methods:** This is a retrospective, descriptive and analytical study conducted between 01.01.2018 and 30.09.2021 in the Radiology and Medical Imaging Department of Fundeni Clinical Institute. 35 patients, each with one liver imaging reporting and data system (LI-RADS) 4- or 5-treated lesion, were eligible to be included. Volumetry was performed using OsiriX MD. Initial lesion volume, ideal necrosis volume (with 0.5–1 cm safety margin) and actual necrosis volume at 1 and 6 months post-procedure were measured.
**Results:** Actual necrosis volume is influenced by several factors: lesion position, proximity to great vessels or other critical structures and etiology of the liver disease. The overall recurrence-free survival rate was similar to data in other studies (77% in the first year post-RFA). This study showed that having the actual necrosis volume at 1 month smaller than ideal necrosis volume is a good predictor for local tumor progression ($p < 0.05$).
**Conclusion:** Volumetric assessment of necrosis is easily attainable and can help improve the prediction of local tumor progression which could lead to earlier diagnosis of LTP.

### SS 2.2

**Sarcopenia impacts on long-term outcomes of radiofrequency ablation for HCC**

J. Choi, B. Park, J. Kim, J. Lee, C. Lee, K. Kim; *Seoul/KR*

**Purpose:** To evaluate impacts of CT-assessed sarcopenia in HCC patients undergoing radiofrequency ablation (RFA) as a first-line treatment.
**Material and Methods:** This study was approved by the International Review Board of our institution. For consecutive 5 years, totally 124 patients who underwent RFA for untreated HCC were included. Cross-sectional area (CSA) of the skeletal muscles at L3 level on CT was measured and normalized for height. CSA less than 52.4 for men and 38.5 for women was diagnosed as sarcopenia. Overall survival (OS), disease-free survival (DFS), and marginal recurrence (MR) were compared between sarcopenic (SC) and non-sarcopenic groups (NSC). In addition, independent factors affecting sarcopenia were evaluated. Statistical analysis was made.
**Results:** The mean age of the patients was 60.3 years (range 36–89). There were 68 male and 56 female. The mean size of HCC was 1.76 cm (range 0.7–4.5). 68 patients were in SC and 56 in NSC. OS was 43.9 months in SC; 43.1 in NSC (p=0.735). DFS was 26.9 months in SC; 29.1 in NSC (p=0.515). MR was 11.8% in SC; 3.6% in NSC (p=0.092). There were no statistical differences between the two groups. The independent factor(s) for OS was international normalized ratio of prothrombin time (p=0.006); for DFS was HCC size (p=0.01); for MR were sarcopenia, HCC size, and total bilirubin (p=0.02, p=0.002, p=0.03, respectively).
**Conclusion:** Sarcopenia is one of the factors affecting MR, but did not impact OS and DFS. We believe RFA could be a preferential treatment modality when a HCC patient is sarcopenic.

### SS 2.3

### The accuracy of PET–MRI in the evaluation of treatment response following radioembolisation

S. Server, O. Ulusoy, M. Cantasdemir, N. Inan Gurcan; *Istanbul/TR*

**Purpose:** We aimed to determine the accuracy of fluorine-18 deoxyglucose positron emission tomography (FDG) PET–MRI in the evaluation of response following radioembolization of liver malignancies.
**Material and Methods:** This study included 15 patients (mean age, 62 years; 10 men, 5 women) with HCC (n=6), ICC (n=3), NET (n=3), CRM (n=3) treated by TARE who underwent pre-treatment and 12 weeks following treatment FDG PET–MRI. We compared the accuracy of MRI imagings (diffusion-weighted imaging (DWI) and contrast-enhanced sequences) with fused hybrid images (CE-PET and DWI-PET images) and pre- and post-treatment SUV max value of lesions as well. The CE PET–MRI protocol was performed by acquiring axial serial CE 3D FS VIBE images in the upper abdomen, completing the whole body in the late phase in the axial plane (Σ = 30 min). Response was analyzed using mRECIST criteria.
**Results:** All images with diagnostic quality for PET and MRI were evaluated separately and as fused images. They were statistically significant in predicting residual tumor detected by the MRI sequences and hybrid images ($p < 0.05$). Residual tumor was detected in two patients by hybrid images. Furthermore, there was a significant decrease in SUV max values on post-treatment images.
**Conclusion:** Hybrid images including DWI, CE-MRI and PET could provide complementary information and facilitate improved clinical decision-making.

### SS 2.4

### Prognostic value of tumor response in patients with advanced HCC treated with selective internal radiation therapy

M. Dupuis, J. Gregory, L. Garzelli, M. Dioguardi Burgio, M. Bouattour, R. Lebtahi, V. Vilgrain, M. Ronot; *Clichy/FR*

**Purpose:** To assess the association between tumor response and survival in patients with HCC treated by selective internal radiation therapy (SIRT).
**Material and Methods:** 102 patients (83 men, 81%, median 71 yrs (IQR 60–77) with HCC treated with SIRT between 2018 and 2020 were included. Most patients had advanced-stage HCC (BCLC-C 88%), unilobar (59%), multifocal (58%), with portal venous tumor thrombosis (51%) and a median (IQR) size of index lesion of 63mm (34–85mm). Response was assessed retrospectively using response evaluation criteria in solid tumors (RECIST 1.1), modified-RECIST (mRECIST), and the liver imaging reporting and data system (LI-RADS) treatment response algorithm (LR-TRA) on the 3-month follow-up CT. Uni-and multivariate Cox-regression and Kaplan–Meier survival analyses were used to explore differences in progression-free (PFS) and overall survival (OS) between responders and non-responders for response criteria.
**Results:** Median follow-up was 32.0 months (95%CI: 16.8–60.9), during which 62/102 patients died (61%), and 90/102 patients showed tumor progression (88%). The median OS and PFS were 21 months (95%CI: 11.5–38.2) and 5 months (95%CI: 2.74–9.13), respectively; 1-year OS and PFS rates were 68% and 86%, respectively. Tumor response was not associated with OS in univariate analysis, whatever the criteria. An objective response per mRECIST and nonviable tumor per LR-TRA were associated with PFS (HR 0.52, 95%CI: 0.35–0.79 and 0.50, 95%CI: 0.31–0.80, respectively). RECIST was not associated with PFS. On multivariate analysis, OS was associated with age, ECOG and cirrhosis status, BCLC stage, and bilirubin but not with tumor response, whatever the criteria.
**Conclusion:** Response per mRECIST and the LR-TRA at 3 months after SIRT for HCC are associated with PFS, but RECIST is not. However, the response is not an independent prognostic factor, whatever the assessment criteria.

### SS 2.5

### The added value of morphologic criteria on CT to assess treatment response of liver metastases after stereotactic body radiotherapy

M. El Homsi, D. Hamdan, F. Ahmed, N. Horvat, C. Hajj; *New York, NY/US*

**Purpose:** To assess treatment response of liver metastases after stereotactic body radiotherapy (SBRT) using criteria of response in solid tumors (RECIST 1.1) and morphologic groups (MG) on CT scan.
**Material and Methods:** Retrospective study including 26 consecutive patients with liver metastases treated with SBRT. Patients had 4 follow-up (FU) CT scans. Assessment of treatment response was performed using RECIST1.1 and MG, defined as follows: group 1, homogeneous and hypoattenuating lesion with sharp contour; group 2, mixed overall attenuation with variable contour; and group 3, heterogeneous attenuation with ill-defined contour. Regression from group 3/2 to 1 is optimal response, from group 3 to 2 is incomplete response, and no change from group 2/3 or progression reflect no response. Chi-square test was used to assess association between RECIST1.1 and response based on MG (RBMG) and association of each method with complete response (CR).
**Results:** Total of 26 patients with liver metastases (median size 3.39cm) were followed for a median of 368 days over 4 intervals. 16 had CR and 9 had incomplete response/progression. There was significant association between RECIST 1.1 with CR at 2nd, 3rd and 4th FU (p value 0.03, 0.01, and 0.003) and between RBMG and CR (p value 0.03, <0.001 and <0.001); no significant association of either method with CR on 1st FU. There was significant correlation between RECIST1.1 and RBMG on the 3rd and 4th intervals (p value 0.006 and 0.0024, respectively).
**Conclusion:** MG of liver metastasis response to SBRT is associated with RECIST 1.1 and CR, with successive increase in significance upon further follow-up imaging.

### SS 2.6

### Recurrence after thermal ablation of liver metastases: does it affect the survival of patients with colorectal carcinoma?

V. Valek[1], T. Rohan[1], T. Andrasina[1], P. Matkulcik[1], J. Zavadil[2]; *[1]Brno/CZ, [2]Ostrava/CZ*

**Purpose:** To analyse the role of routine PET/MR follow-up after thermal ablation of liver metastases in patients with colorectal carcinoma.
**Material and Methods:** Patients treated with radiofrequency or microwave thermal ablation from January 2013 to December 2022 were included in this retrospective study. Finally, local tumor recurrence and survival time were analyzed in patients followed up regularly with PET/MR (since 2016). Log-rank test was used to compare survival between patients with and without local recurrence after liver ablation. Group characteristics were compared using Mann–Whitney and Fisher exact tests.
**Results:** 94 patients (62 men, 63±11 years) were treated for 181 liver metastasis. PET/MR was used for regular follow-up in 42 patients (24 men (61±11 years)) with 78 treated tumors. In this group, local recurrence after ablation was observed in 37 ablation zones (in 24 patients, 13 men (p=0.346), 59±14 years (p=0.822)). 10 of them underwent reablation. Tumors were nonsignificantly larger in the recurrent group (19.8mm vs 16.5mm, p=0.184). The time between ablation and disease progression was shorter in recurrent group (135±120d vs 228±244d, p=0.142). There was no difference in survival between the two groups (4.3y vs 4.4y, p=0.8). The time to detect progressive disease was longer in patients where CT was used for follow-up (294.3±256.1d, p=0.054).
**Conclusion:** Using PET/MR, earlier detection of recurrence in the ablation zone of liver metastasis could allow early reintervention. This could hypothetically equalize survival in this less favorable group compared with patients without local recurrence.

### SS 2.7

### The use of strain elastography in Klatskin tumor biopsies: preliminary results

M. Ozdemir, U. Koc, M. Gokhan, M. Besler; *Ankara/TR*

**Purpose:** This study aims to demonstrate the experience of our center in the use of strain elastography in terms of specimen adequacy and complications in US-guided biopsies with suspected Klatskin tumor.
**Material and Methods:** After ethics committee approval (E2-22-2212), 16 liver biopsies performed with the suspicion of Klatskin tumor by one interventional radiologist between March 2019 and July 2022 were evaluated retrospectively. The elastographic and sonographic features of lesions were examined. The strain index values were calculated. 7 patients who underwent biopsy with elastography were compared with 9 patients without, in terms of complications and of biopsy adequacy. $p<0.05$ was accepted as the statistical significance level.
**Results:** The mean age of the patients was 64.9±9.19 and 12 were female and 4 were male. In gray scale, 3 lesions were hyperechoic, 5 were iso-hyperechoic, 3 were isoechogenic, 4 were iso-hypoechoic, and 1 was hypoechoic. Mean strain index was found to be 4.15±0.41 in lesions with elastography. While there was no need for repeat biopsy in biopsies performed using elastography, re-biopsy was recommended in 2 biopsies performed with gray scale. However, there is no significant difference between the groups (p=0.475). No post-biopsy complication was observed in either group.
**Conclusion:** In isoechoic lesions, using elastography provides convenience in determining the lesion boundaries. The use of elastography for Klatskin could be helpful in biopsy and diagnosis.

### SS 2.8

### 10-Year outcomes from CT-guided retroperitoneal biopsy: how effective? How safe?

D. Bradley[1], S. Billingsley[2], C. Walklett[2], J. Cairns[2], D. Tolan[2]; [1]*Bradford/GB*, [2]*Leeds/GB*

**Purpose:** CT retroperitoneal biopsy (CT-RB) is a challenging interventional technique that is essential to guide patient management in benign and malignant diseases in the abdomen and pelvis. The study will determine the 10-year outcomes and complications from CT retroperitoneal biopsy (CT-RB) to inform patient consent and clinical service design.
**Material and Methods:** 312 consecutive CT-RB procedures between 2010 and 2021 were evaluated to correlate with histopathology to determine sample yield for final diagnosis, frequency of complications, tumour seeding and time to death post-procedure.
**Results:** CT-RB cohort consisted 170 men and 142 women with mean age 62 (range 21–93). Adequate samples were obtained with a coaxial needle in 89% procedures with conclusive histological diagnosis including tumour subtype in 74%. There was no significant difference in sample adequacy related to needle size (p=0.1209) or number of passes (p=0.1375). Additional biopsies were required in 5.8% to achieve a conclusive diagnosis. Complications occurred in only 4 out of 312 (1.3%) cases and none required additional treatment or admission. All complications occurred within 1 hour post-CT-RB. No cases of tumour seeding were identified on any follow-up imaging of CT-RB sites.
**Conclusion:** CT-RB is safe with a high diagnostic yield for MDT decision-making with no serious or long-term complications or risk of tumour seeding. Patients have a 1% risk of complications occurring within 1 hour of the procedure and 6% will need additional biopsy. There is no evidence that more than 1 hour of patient recovery is necessary after biopsy to detect procedural complications.

### SS 2.9

### Transarterial embolization in the management of lower GI tract bleeding: single-centre experience

K. Nowak, B. Regent, E. Szurowska, T. Gorycki; *Gdansk/PL*

**Purpose:** Defined as below the ligament of Treitz, lower GI bleeding (LGIB) is a potentially life-threatening condition. While endoscopic management is not always sufficient in localizing and stopping the bleeding, endovascular approach is considered the next go-to treatment. As transarterial embolization (TAE) is still a relatively novel, minimally invasive procedure, the aim of this study was to investigate whether TAE is an effective and safe method for gaining hemostasis in LGIB.
**Material and Methods:** We analyzed a study group of 24 patients who underwent TEA as a treatment of LGIB between January 2018 and December 2022 in UCK Gdańsk, Poland. Prior to TAE, patients were diagnosed with LGIB based on angio-CT or endoscopic examination, which were successful in localizing active bleeding in 10 and 12 cases, respectively. A total of 26 TAE procedures were conducted via standard femoral access, proceeded with direct arteriography which revealed active extravasation in 19 cases and pathological enhancement in 7. Embolizing materials included microcoils (15), glue (8), microspheres (2), PHIL liquid (1) and gelatin sponge (3).
**Results:** In control arteriography, complete hemostasis was achieved in 100% of cases. Complications included relapse of bleeding, further anemization or bowel necrosis, which occurred in 2 (7.7%), 3 (11.5%) and 2 (7.7%) cases, respectively. Postoperative mortality, defined as death within 30 days post-TEA reached 4 (15.4%) cases, where none of them was directly linked to the procedure but were caused by other comorbidities (multiple organ failure, sepsis, terminal cancer).
**Conclusion:** TAE is an effective method for the management of LGIB with low complication rate. Endovascular approach seems to be a useful tool in localizing active LGIB.

**11:00 - 12:30** **Room 1**

### *Scientific Session SS 3*

### Pancreatic adenocarcinoma: detection, staging and treatment response prediction

### *SS 3.1*

### Short protocol MRI for intraductal papillary mucinous neoplasm follow-up: time has come

L. Geraci, L. Tomaiuolo, E. Bardhi, D. Autelitano, A. Carli, M. Todesco, N. Cardobi, R. De Robertis, M. D'Onofrio*; Verona/IT*

**Purpose:** To validate MRI short-protocols analyzing three short-protocol performance for intraductal papillary mucinous neoplasms (IPMNs) follow-up and to compare them to the traditional one in assessing patient's management.
**Material and Methods:** 223 patients with IPMNs studied with 1.5 T MRI with T2-SSH TSE, T2 fat-sat, T1-FFE fat-sat, DWI-EPI, T2-SSH TSE CPRM and post-contrast T1-THRIVE sequences were identified. 896 randomized images were extracted and grouped into four different protocols as below: T1, T2w and T2-CPRM sequences (protocol 1), T1, T2w and T2-CPRM and DWI sequences (protocol 2), T1, T2w, T2-CPRM and post-contrast T1-sequences (protocol 3), a comprehensive protocol including all sequences (protocol 4). Three blinded radiologists with different experience (20 years for I reader and 10 years for II and III readers) were requested to suggest the patient's management with a subsequent follow-up, a further exam or a surgery valuation. The complete protocol was then compared to the sequence-shortened ones and the interobserver agreement was calculated with Cohen's kappa test.
**Results:** For the more expert reader, protocols 1 and 2 result in a substantial agreement, 0.63 and 0.72, respectively, with clinical patient's management. Comparison between readers showed moderate to substantial agreement for protocol 1 (0.50 I vs II reader, 0.71 I vs III reader) and moderate to substantial agreement for protocol 2 (0.54 I vs II reader, 0.68 I vs III reader). Substantial to almost perfect agreement for protocol 4 (0.78 I vs II reader, 0.85 I vs III reader) was found.
**Conclusion:** MRI short protocols reveal substantial agreement to final decision for the expert reader. For short protocols, moderate to good interobserver agreement was found.

### *SS 3.2*

### Computed high b value high-resolution diffusion-weighted imaging improves solid lesion detection in intraductal papillary mucinous neoplasm of the pancreas

F.N. Harder, E. Jung, K. Weiss, V. Phillip, E.I. Demir, M.R. Makowski, D.C. Karampinos, R. Braren*; Munich/DE*

**Purpose:** To examine the effect of high b value computed diffusion-weighted imaging (cDWI) on solid lesion detection and classification in pancreatic intraductal papillary mucinous neoplasm (IPMN), using EUS and histopathology as a standard of reference.
**Material and Methods:** Eighty-two patients with IPMN were retrospectively enrolled. Computed high b value images at b = 1000 s/mm$^2$ were calculated from standard (b = 0, 50, 300 and 600 s/mm$^2$) DWI images for conventional full field-of-view (fFOV, 3x3x4 mm$^3$ voxel size) DWI. A subset of 39 patients received additional high-resolution reduced field-of-view (rFOV, 2.5x2.5x3 mm$^3$ voxel size) DWI. In this cohort, rFOV cDWI was compared against fFOV cDWI additionally. Two experienced radiologists evaluated (Likert Scale 1–4) image quality (overall image quality, lesion detection and delineation, fluid suppression within the lesion). In addition, quantitative image parameters (apparent signal-to-noise ratio (aSNR), apparent contrast-to-noise ratio (aCNR), contrast ratio (CR)) were assessed. Diagnostic confidence regarding the presence/absence of diffusion restricted solid nodules was assessed in an additional reader study.
**Results:** High b value cDWI at b = 1000 s/mm$^2$ outperformed acquired DWI at b = 600 s/mm$^2$ regarding lesion detection, fluid suppression, aCNR, CR and lesion classification (p = <.001–.002). Comparing cDWI from fFOV and rFOV revealed higher image quality in high-resolution rFOV-DWI compared to conventional fFOV-DWI (p = <.001–.018). High b value cDWI images were rated non-inferior to directly acquired high b value DWI images (p = 0.095–.655).
**Conclusion:** High b value cDWI may improve detection and classification of solid lesions in IPMN. Combining high-resolution imaging and high b value cDWI may further increase diagnostic precision.

### *SS 3.3*

### Imaging assessment of potentially malignant mucinous cystic neoplasm of pancreas using Revised 2017 International Consensus Guidelines: comparison with branch-duct type intraductal papillary neoplasm

H. Kim, J. Kim, J. An, J. Choi*; Seoul/KR*

**Purpose:** Evaluating diagnostic performance of Revised 2017 International Consensus Guideline applied to preoperatively predict malignant MCNs compared with BD-IPMN.
**Material and Methods:** 224 patients with surgically confirmed MCNs (benign 73; malignant 17) and BD-IPMNs (benign 110; malignant 24) were retrospectively enrolled. All had contrast-enhanced CT or MRI before the pancreatectomy. Two radiologists analyzed CT findings suggestive of high-risk stigmata or worrisome features as proposed by 2017 International Consensus Guidelines. Multivariate analyses were performed to identify significant predictors of malignant MCNs.
**Results:** MCNs and BD-IPMNs were different in size, location, enhancing wall or septal thickening, and multiplicity (p <0.05). Only tumor size (OR, 1.335; 95% CI, 1.139–1.566, p <0.001) was significantly different between malignant and benign MCNs while size of mural nodule (OR, 1.203; 95% CI, 0.102–0.313, p < 0.001) was different in BD-IPMNs. The optimal cut-off tumor size differentiating benign from malignant MCNs was 8.95 cm having 70.6% sensitivity and 89% specificity with diagnostic accuracy 85.6%. With a 4.0 cm threshold based on the International Consensus Guideline 2012, sensitivity was 94.1% but specificity was as low as 42.5%. For BD-IPMNs, 5.5 cm was the best cut-off value distinguishing benign from malignant tumors with sensitivity 50%, specificity 93.6%, and accuracy 85.8%.
**Conclusion:** The maximal diameter of a tumor shows the association with the malignant MCNs and the optimal threshold 8.95 cm, much larger than 4 cm widely suggested by many guidelines. Size could be considered with priority for the initial risk stratification and for deciding the optimal management of MCNs.

### *SS 3.4*

### Analysis of adenocarcinomas of the pancreatobiliary type and neuroendocrine tumors of the pancreas using volumetric apparent diffusion coefficient histograms

M. Nalbant, E. Inci*; ISTANBUL/TR*

**Purpose:** We aimed to investigate the value of volumetric ADC histogram analysis in the differentiation of pancreatobiliary type adenocarcinomas (PPAC) and neuroendocrine tumors (pNET).
**Material and Methods:** A total of 524 patients were evaluated retrospectively. Final enrollment included 90 patients with histopathologically proven PPAC (n = 54) and pNET (n = 36), with a mean age of 61.67±8.23 years. MRI was acquired at 1.5 T (Verio; Siemens Medical Solutions, Erlangen, Germany), and diffusion-weighted imaging was obtained at b values of 1000 mm²/s. The histogram parameters of ADC values, comprising the mean, minimum, maximum, 5th, 10th, 25th, 50th, 75th, 90th, and 95th percentiles, as well as skewness, kurtosis, and variance, were calculated.
**Results:** ADCmin, ADCmean, and ADCmax, as well as the percentile values of ADC for the PPAC group, were all lower than those of the pNET group. The difference between ADCmax (P<0.001), 90th, and 95th percentiles of ADC values (P<0.05) was statistically significant. The PPAC group had higher variance and skewness than the pNET group (P<0.001). The area under the curve (AUC) of the variance was the highest (AUC = 0.744; cut-off 214.71; sensitivity 81.4%; and specificity 80.4%), followed by ADCmax (AUC = 0.679; cut-off 2.052 x 10-3 mm²/s; sensitivity 74.3%; and specificity 77.8%) and skewness (AUC = 0.642; cut-off 0.303; sensitivity 67.1%; and specificity 74.1%).
**Conclusion:** Volumetric ADC histogram analysis with b values of 1000 mm²/s can discriminate PPAC and pNET groups. Histogram examination of the whole tumor is a novel and valuable diagnostic technique.

### SS 3.5

### Imaging features of adenosquamous carcinomas of the pancreas compared to pancreatic ductal adenocarcinomas

C. Wild, P. Mayer, J. Kaiser, M. Klauß, H.-U. Kauczor; *Heidelberg/DE*

**Purpose:** Adenosquamous carcinoma of the pancreas (ASP) is a rare subtype of solid pancreatic neoplasms and accounts only for 1–4% of exocrine pancreatic tumors. Compared to the common pancreatic ductal adenocarcinoma (PDAC), ASP is more aggressive and associated with a worse prognosis. The present study aims to compare CT and MRI features of ASP and PDAC.

**Material and Methods:** 83 patients with ASP treated at Heidelberg University Hospital between December 2001 and December 2017 were retrospectively identified from a prospectively maintained database. 83 patients with PDAC served as comparison group. Lesion imaging features were retrospectively extracted from 145 CT scans and 48 MRI scans.

**Results:** Both ASP and PDAC presented as lobulated lesions with ill-defined borders. Ring enhancement was significantly more often observed in ASP than in PDAC. Post-contrast central hypodensity/T1-hypointensity indicating extensive tumor necrosis was more prevalent in ASP. Locoregional lymph node enlargement and distant metastases were observed to a similar extent in both groups. There was no difference in patient age or tumor location within the pancreas.

**Conclusion:** Due to the rareness of ASP, most of published literature is based on small patient cohorts or case reports. To our knowledge, this is the first study which identifies key radiologic characteristics of ASP and PDAC in a large patient cohort. Our results suggest that ring enhancement and extensive central tumor necrosis are strong imaging differentiators between ASP from PDAC.

### SS 3.6

### Automatic assessment of vascular involvement on CT in patients with localized pancreatic cancer using artificial intelligence

J. Bereska, I. Verpalen, Y. Nio, M. Kop, F. Struik, H. Marquering, M. Besselink, J. Stoker; *Amsterdam/NL*

**Purpose:** The aim of this study was to develop and evaluate a fully automatic framework for determining vascular involvement in patients with pancreatic ductal adenocarcinoma (PDAC) using artificial intelligence. Resectability of PDAC is determined by the degree of vascular involvement on CT scans (CTs), but has considerable inter-observer variability. A fully automatic framework to assess individual vascular involvement would reduce inter-observer variability but is currently lacking.

**Material and Methods:** We developed a self-learning-based deep learning model to segment PDAC, celiac trunk, hepatic artery, superior mesenteric artery, portal vein, and superior mesenteric vein in patients with PDAC. We applied a geometric calculation on the segmented structures to quantify vascular involvement (0–360 degrees). We subsequently classified the PDAC resectability stage (resectable, borderline resectable, non-resectable) using the resulting degrees of involvement and the Dutch Pancreatic Cancer Group (DPCG) PDAC resectability criteria. We compared the automated involvement measure with reference-truth resectability classification provided by experienced abdominal radiologists following categorization.

**Results:** Overall, we included 563 CTs of 467 patients with PDAC and 50 control patients. Our automated framework achieved an agreement of 227/300 (76%) patients with the reference-truth classifying the degrees of vascular involvement for individual vessels. Using the resulting degrees of vascular involvement and the DPCG criteria, we determined PDAC resectability with an agreement of 46/60 (80%).

**Conclusion:** These results show that state-of-the-art deep learning models may aid radiologists in determining PDAC resectability by providing a fully automatic quantification of vascular involvement in patients with PDAC.

### SS 3.7

### Intrascanner reproducibility of MRI radiomics features for pancreas cancer

F. Ichinohe, K. Oyama, T. Aonuma, T. Nonaka, T. Fukuzawa, Y. Fujinaga; *Matsumoto/JP*

**Purpose:** Although radiomics is a promising analysis for MRI, intrascanner reproducibility has not been fully evaluated because multiple MRIs of the same lesion are not usually acquired. The purpose of this retrospective study is to evaluate intrascanner reproducibility of MRI radiomic features for pancreatic cancer.

**Material and Methods:** Twenty patients with pathologically proven pancreatic cancer between April 2018 and November 2022 were enrolled in this study. They underwent two contrast-enhanced MRIs within one month: with extracellular agents for evaluation of the pancreatic cancer using a 3-T scanner and with hepatobiliary agents for detection of liver metastases using the other type of 3-T scanner. Two radiologists set regions of interest by consensus on the lesions on unenhanced fat-suppressed T1-weighted images using the Dixon method (T1WI) and T2-weighted images (T2WI). We calculated 18 first-order features and 75 second-order features of the lesions with syngo.via Frontier Radiomics (Siemens Healthcare, Erlangen, Germany). We evaluated the intrascanner reproducibility of them using intraclass correlation coefficients (ICC).

**Results:** Out of 18 first-order features, 4 features (22%) in T1WI and 7 features (39%) in T2WI showed good reproducibility ($ICC > 0.75$). Out of 75 second-features, 12 features (16%) in T1WI and 18 features (24%) in T2WI showed good reproducibility ($ICC > 0.75$).

**Conclusion:** Intrascanner reproducibility of MRI radiomics in pancreatic cancer is insufficient, and this would be an obstacle to the clinical application of radiomics studies.

### SS 3.8

### Repeatability of MR elastography of the pancreas in healthy volunteers and pancreatic ductal adenocarcinoma patients

N.P. Wassenaar, A.-S. van Schelt, E.M. Schrauben, H.W. van Laarhoven, J. Stoker, A.J. Nederveen, J.H. Runge; *Amsterdam/NL*

**Purpose:** MR elastography (MRE) can measure visco-elastic properties of the pancreas. The ability to differentiate between healthy and diseased tissue and/or assess treatment response, falls or stands with robust MRE. Our aims were to assess (I) MRE intra- and intersession repeatability in pancreatic ductal adenocarcinoma (PDAC) patients and healthy volunteers (HV) and (II) the ability to distinguish between healthy and malignant pancreatic tissue.

**Material and Methods:** In total, 8 pathological-proven PDAC patients (independent of staging or prior treatment) and 8 age- and gender-matched HV underwent two back-to-back consecutive MRE scans (MRE-A and B) following MRE-C after repositioning. The shear-wave-speed (SWS) and phase-angle ($\phi$) were calculated for healthy pancreas and tumor. One-way repeated measures ANOVA was used to determine intra- and intersession repeatability. Intra- and intersession coefficient of variation (CV) were also calculated. Unpaired t test was used to test for differences between HV and patients.

**Results:** SWS and $\phi$ did not significantly differ between the three MRE scans for patients and HV (Patients[SWS/$\phi$]: $F(2,14)=[0.63/1.64]$, $p=[0.55/0.23]$; HV[SWS/$\phi$]: $F(2,14)=[2.42/1.90]$, $p=[0.13/0.19]$). The intra- and intersession CV for SWS/$\phi$ were 10.4%/8.0% and 12.9%/10.5% for patients and 5.1%/2.8% and 8.1%/3.7% for HV. A significant difference between patients and HV was found for SWS at 1.89 versus 1.10m/s ($p<0.001$) and $\phi$ at 1.17 versus 0.81 radian ($p<0.001$).

**Conclusion:** No significant differences were found between repeated scans for both visco-elastic parameters and stiffness values were comparable to the literature. The intrasession CVs should be taken into account when using pancreatic MRE for treatment response assessment in PDAC.

### SS 3.9

### Quantitative MRI in pancreatic ductal adenocarcinoma patients and healthy volunteers

A.-S. van Schelt, N.P. Wassenaar, E.M. Schrauben, H. van Laarhoven, A.J. Nederveen, J.H. Runge, J. Stoker; *Amsterdam/NL*

**Purpose:** Pancreatic ductal adenocarcinoma (PDAC) has a poor prognosis. Of the patients receiving chemotherapy, only 20% become eligible for surgery. Quantitative MRI can non-invasively assess tumor microenvironment, which plays a large role in treatment efficacy. The aim of this study was to determine predictive markers within PDAC between treatment naive/chemotherapy and tumor staging using T2*, IVIM and MR elastography (MRE).
**Material and Methods:** MR was performed in 18 patients with PDAC (67% male, age=65±11 years, BMI=23.7±3.1 kg/m$^2$), 50% received prior chemotherapy (staging: 33%(resectable)/44%(LAPC)/22%(metastatic)) and 11 healthy volunteers (HV) (73% male, age=62±12 years, BMI=25.6±2.1 km/m$^2$). Mean values were determined for T2* (deoxygenation), IVIM resulted in D (diffusion) and f (perfusion) and MRE in SWS (stiffness) and φ (viscosity). One-way ANOVA was done for prior treatment and staging. Student's t test was used to determYangine differences between patients and HV.
**Results:** Mean values for PDAC were SWS=1.95±0.61 m/s, φ=1.11±0.17 radian, T2*=50±16 ms, D=1.58±0.32·10$^3$ mm$^2$/s and f=0.11±0.08%. One-way ANOVA showed no effects for T2*, SWS and φ of prior treatment, whilst D and f significantly increased (D:p=0.018, f:p=0.046). SWS showed significant differences between resectable and LAPC (SWS=1.41/2.36m/s, p=0.009). SWS, φ and T2* revealed a significant increase when compared to HV (p≤0.001), while f showed a significant decrease (p=0.001).
**Conclusion:** IVIM showed differences for prior treatment, which indicates a predictive biomarker for treatment efficacy with diffusion and perfusion increasing after chemotherapy. Hypothesized is that the increased SWS for LAPC compared to resectable tumors could be due to the dense stromal deposition in LAPC. Differences in T2*, f, SWS and φ for PDAC compared to HV are in line with the literature.

### SS 3.10

### Diffusion-weighted imaging for predicting response to neoadjuvant therapy in pancreatic cancer: a systematic review

C. Bilreiro, L. Costa Andrade, T. Bilhim, C. Matos; *Lisbon/PT*

**Purpose:** To assess the value of diffusion-weighted MRI, using apparent diffusion coefficient (ADC), for predicting response to neoadjuvant therapy (NT) in pancreatic cancer (PC), including chemotherapy, radiotherapy, or both.
**Material and Methods:** MEDLINE, Cochrane Library and EMBASE databases were searched for studies evaluating ADC for NT evaluation in PC. Data were extracted regarding the use of ADC pre- and/or post-NT, for predicting response as defined by imaging, histopathology, or clinical reference standards. Risk of bias was assessed using the Quality Assessment of Diagnostic Studies (QUADAS-2).
**Results:** From 315 initially yielded studies, 11 were included in the analysis (299 patients). Five studies reported pre-treatment ADC, overlapping between responders and non-responders: 1.00 to 1.61 x 10$^{-3}$mm$^2$/s, and 1.24 to 1.5 x 10$^{-3}$mm$^2$/s, respectively. Three studies reported ADC values post-NT: 1.28 to 1.42 x 10$^{-3}$mm$^2$/s for responders and 1.17 to 1.3 x 10$^{-3}$mm$^2$/s for non-responders. Three studies reported ADC increase after NT: 14.9 to 27.0% for responders and 2.3 to 10.3% for non-responders. Correlation with histopathological response was reported for pre-treatment ADC in 3 studies (r = 0.63 to 0.94). Finally, high risk of bias was identified across studies, mostly in the domain of reference standard, but also regarding patient selection and flow and timing. Regarding applicability, concerns were expressed in the domains of patient selection and reference standard. Pooling analyses were not performed due to heavy heterogeneity.
**Conclusion:** An increase in ADC post-NT might be useful for predicting response, but ADC values pre- and post-treatment overlap for responders and non-responders. Nevertheless, studies were heterogeneous and high risk for bias was identified.

**14:30 - 16:00** **Room 1**

### *Scientific Session SS 4*
### Liver steatosis and liver function

### SS 4.1

### Prospective comparison of ATI and CAP for the diagnosis of liver steatosis in patients with non-alcoholic fatty liver disease, type 2 diabetes, and at risk for non-alcoholic steatohepatitis

M. Dioguardi Burgio[1], L. Castera[1], M. Oufighou[1], P.-E. Rautou[1], V. Paradis[1], P. Bedossa[2], R. Sartoris[1], M. Ronot[1], P. Garteiser[2], B. Van Beers[1], V. Vilgrain[1], J.-M. Correas[2]; *[1]Clichy/FR, [2]Paris/FR*

**Purpose:** To prospectively compare US-based attenuation imaging (ATI) and controlled attenuation parameter (CAP) performances for the diagnosis of hepatic steatosis in patients with type 2 diabetes (T2D) non-alcoholic fatty liver disease (NAFLD) and at risk for non-alcoholic steatohepatitis (NASH) using histology and MRI proton density fat fraction (PDFF) as references.
**Material and Methods:** ATI, CAP, MRI, and biopsy were obtained on the same day. Steatosis was classified as S0, S1, S2, and S3 at histology (<5%, 5–33%, 33–66%, and >66%, respectively), while for MRI-PDFF, the thresholds of 6.4%, 17.4%, and 22.1% were used. Comparison of ROC curve was done with the Delong test.
**Results:** Liver steatosis was evaluable in 191 and 187 patients with liver biopsy and MRI-PDFF, respectively. The areas under the curve (AUC) of ATI were 0.92 (95%CI 0.87–0.95) and 0.86 (95%CI 0.81–0.91) for the diagnosis of histological and MRI-PDFF steatosis (S0 vs. S1–S3), respectively. When MRI-PDFF was used as a reference, ATI outperformed CAP [AUC 0.69 (95%CI 0.62–0.75)] for the diagnosis of steatosis (S0 vs. S1–S3, p=0.02) but no difference was found for the diagnosis of S2–S3 steatosis (p=0.60). When histology was used as a reference, ATI and CAP showed similar performance with AUC differences of 0.03 for both S0 vs. S1–S3 and S0–S1 vs. S2–S3 (p=0.64 and p=0.61, respectively).
**Conclusion:** In patients with T2D and NAFLD and at risk for NASH, ATI outperformed CAP for the diagnosis of hepatic steatosis (S1–S3) when liver MRI-PDFF was used as a reference and may be used as a first-line method for diagnosing hepatic steatosis.

Scientific Sessions

Authors' Index

### SS 4.2

### Both histopathologic large lipid droplet and microsteatosis contribute to MRI-proton density fat fraction reflecting the whole grade of hepatic steatosis

D. Marti Aguado, M. Fernandez Paton, A. Ten Esteve, A. Jimenez Pastor, L. Cerda Alberich, A. Alberich Bayarri, A. Sanchez Martin, C. Alfaro Cervello, M. Bauza, V. Puglia, A. Perez Girbes, A. Crespo, E. Coello, S. Benlloch, V. Aguilera, D. Escudero Garcia, L. Marti Bonmati*; Valencia/ES*

**Purpose:** MRI-proton density fat fraction (PDFF) is the most accurate non-invasive method for detecting hepatic steatosis. We aimed to evaluate the influence of lipid droplet number and size in liver PDFF values.
**Material and Methods:** Prospective, cross-sectional, multicentre study including chronic diffuse liver disease patients with paired liver biopsy and MRI between 2017 and 2022. MR examination included the MECSE sequence to measure PDFF after automatic whole-liver segmentation. Histologic samples were assessed with conventional microscope and digital image analysis (Ventana iScan). Slides were stained with H&E for semiquantitative scoring of steatosis (S0–3 grade and presence of microsteatosis) and IHQ adipophilin for digital pathology. Lipid droplets were categorized as large macrosteatosis (≥67 $\mu m^2$), small macrosteatosis (<67 $\mu m^2$) and microsteatosis (non-droplet positive adipophilin). Fat proportionate areas (FPA) and number of lipid droplets were assessed with digital pathology.
**Results:** There were 206 patients (57% women; age of 55±12 years) with BMI=29 (24–32) and mean PDFF=11.9±4.9%. Main liver disease aetiology was NAFLD (61%) and histologic steatosis distribution was 37% S0, 23% S1, 19% S2, and 21% S3. PDFF values had a stronger correlation with the number of lipid droplets in large macrosteatosis compared to small macrosteatosis (r=0.85 vs. r=0.70; P<0.01). The strength of PDFF–FPA correlation increased with lipid droplet size: r=0.74 for microsteatosis, r=0.78 for small macrosteatosis, r=0.84 for large macrosteatosis; being higher (r=0.88) with total steatosis. PDFF values were higher if microsteatosis was present (10.8% vs. 14.3%; P<0.01).
**Conclusion:** Pathologists grading system of steatosis should unify lipid droplet and microsteatosis assessment as MRI-PDFF correlates with the whole spectrum of hepatic steatosis.

### SS 4.3

### US imaging features of liver involvement of Wilson disease in adult patients

E. Belmonte, C. Garcia, J. Moreno, A. Darnell, J. Rimola, A. Soler, C. Perez, Z. Mariño, M. Garcia Criado*; Barcelona/ES*

**Purpose:** To describe the ultrasonographic (US) features of the liver in patients with Wilson's disease (WD). To assess the correlation between any of the findings and advanced liver disease or laboratory abnormalities.
**Material and Methods:** Retrospective study of patients with WD under clinical follow-up, US and laboratory analysis. 55 patients with WD were included: 28 men (51%), mean age 38y (27–47y), years of follow-up from diagnosis 17y (11.7–27y), 14 (25.5%) with liver cirrhosis, 28 (52.8%) with ASAT and/or ALAT >40UI/L. Statistical analysis was performed with SPSSv.27; variables were expressed in n(%) and mean (IQR 25–75).
**Results:** Liver echogenicity was increased (steatosis) in 31 (56.3%): mild 24 (77.4%), moderate 5 (16.2%), and severe 2 (6.4%). Liver lesions were identified in 11 patients (20%). 5/6 patients with hypoechoic nodules were cirrhotic. 4/6 patients with hypoechoic nodules had more than one nodule. One nodule was an HCC. Periportal echogenicity thickness could be assessed in 40/55 patients. In 14 patients (40%), it was abnormal (>=2mm), none of them with cirrhosis, hypertransaminasaemia or focal liver lesions. Splenomegaly (> 13cm) was present in 19 patients (34.5%), 22% of them without cirrhosis. Splenomegaly was related to lower platelet counts (136 vs 224, p>0.001), but no correlation could be observed with any other analytical or imaging parameters.
**Conclusion:** In this retrospective analysis of US features in patients with WD, steatosis was highly prevalent. Splenomegaly was observed in one third of the patients, also in the absence of cirrhosis. Abnormal thickness of periportal echogenicity is frequent and may increase the suspicion of Wilson´s disease.

### SS 4.4

### Detection of liver steatosis: prospective comparison of B-mode US and US-derived fat fraction with MRI proton density fat fraction

R. De Robertis, F. Spoto, D. Autelitano, A. Olivieri, D. Guagenti, G. Incarbone, P. Zanutto, M. D'Onofrio*; Verona/IT*

**Purpose:** To evaluate B-mode US and US-derived fat fraction (UDFF) for the detection of liver steatosis using MRI-proton density fat fraction (PDFF) as the reference standard.
**Material and Methods:** 129 patients underwent US, UDFF and MRI-PDFF on the same day. A radiologist performed US and 10 UDFF measurements in the right hepatic lobe; a second radiologist drew 10 regions of interest in the right hepatic lobe on the MRI-PDFF map. Agreement and relationships between UDFF and MRI-PDFF measurements were tested with the Bland–Altman method and the Spearman's correlation; sensitivity, specificity, positive and negative predictive values, and accuracy of B-mode and UDFF to detect liver steatosis (MRI-PDFF ≥5.5%) were calculated.
**Results:** Ten patients were excluded owing to MR (N=4) or US (N=6) artifacts; 119 patients were included (63 males, 56 females; median age 60 years). Thirty-one patients (26.1%) had steatosis. There were no significant differences between the overall UDFF and MRI-PDFF measurements as well as between those performed in segments 7–8 (p=0.092 and 0.447; mean bias, −0.43% and −0.23%, respectively). Measurements in segments 5–6 were significantly different between the two methods (p=0.019, mean bias −0.68%). There was a significant positive correlation between UDFF and MRI-PDFF (rho=0.669, p<0.001). Sensitivity, specificity, positive and negative predictive values, and accuracy of B-mode and UDFF for detection of liver steatosis were 58.1/87.1%, 71.6/75%, 41.9/55.1%, 82.9/94.3% and 68.1/78.2%, respectively.
**Conclusion:** UDFF has agreement with MRI-PDFF and has higher diagnostic value than B-mode US for diagnosis of liver steatosis; UDFF measurements in segments 7–8 are more reliable than those in segments 5–6.

### SS 4.5

### Diagnostic performance of US-guided attenuation parameter for the quantification of hepatic steatosis in patients with biopsy-proven nonalcoholic fatty liver disease: a prospective study

R. Cannella, F. Agnello, G. Porrello, A. Spinello, G. Infantino, S. Petta, D. Cabibi, A. Taibbi, T. Bartolotta*; Palermo/IT*

**Purpose:** To assess the performance and the reproducibility of US-guided attenuation parameter (UGAP) for the quantification of hepatic steatosis in patients with biopsy-proven nonalcoholic fatty liver disease (NAFLD).
**Material and Methods:** This prospective study included adult patients with NAFLD who underwent US with UGAP and liver biopsy within two months. UGAP measurements were performed by two independent and blinded radiologists. A total of 12 consecutive measurements were acquired in each patient, which were repeated twice by the first radiologist. The median values were considered for the analysis. Hepatic steatosis was graded at liver biopsy as (0)<5%; (1)5–33%; (2)33–66%; (3)>66%. Area under the receiver operating characteristic (ROC) curve (AUC) with Youden index was calculated to determine the diagnostic performance with optimal cutoff. The intraclass correlation coefficient (ICC) was used to assess the inter- and intra-observer reproducibility.
**Results:** Sixty-one patients (33 females, 53.6±11.4 years) with NAFLD were prospectively enrolled. At histopathological analysis, 15/61 (24.6%) and 26/61 (42.6%) had grade 2 and 3 steatosis, respectively. Median UGAP was 0.77dB/cm/MHz (IQR: 0.05dB/cm/MHz; IQR/median ratio: 5.6%). For the diagnosis of grade≥2 steatosis, median UGAP had an AUC of 0.812 (95%CI: 0.692–0.901, p<0.001), while for the grade 3 steatosis, median UGAP had an AUC of 0.887 (95%CI: 0.768–0.947, p<0.001). The optimal cutoff of >0.75 provided a sensitivity of 78.1% and a specificity of 85.0% for the diagnosis of grade≥2 steatosis. The inter- and intra-observer reproducibility were excellent with an ICC of 0.93 (95%CI: 0.87–0.96) and 0.95 (95%CI: 0.91–0.97), respectively.
**Conclusion:** UGAP measurements provide a good performance for the diagnosis of moderate to severe steatosis in patients with NAFLD with excellent reproducibility.

### SS 4.6

### Quantitative analysis of liver proton density fat fraction with deep learning on low-dose CT

H.-J. Chung[1], K. Jong-Min[1], J. Lee[2], J. Yoon[2], S. Park[1]; [1]Medical IP/KR, [2]Seoul/KR

**Purpose:** The purpose of this study is to evaluate a deep learning-based method for estimating liver proton density fat fraction (PDFF) maps from low-dose CT (LDCT) images.
**Material and Methods:** The dataset, which our PDFF estimator trained with, only contains small CT dataset (train: 18 cases, validation 2 cases) acquired from conventional CT Scanner (Brilliance, Philips). For low-dose simulation, the Poisson noise was inserted into projection of CT images to create a noise-corrupted sinogram at a quarter-dose level. To reconstruct LDCT images, the additional denoiser was trained using the American Association of Physicists in Medicine (AAPM) low-dose CT challenge dataset. 145 adult clinical patients who had both CT (IQon, Philips Healthcare) and MRI (Skyra 3.0T, Siemens Healthineers) scan were retrospectively included for external validation. The performance of our estimation method was evaluated using R-squared metric. For measuring liver PDFF, the liver mask from vendor-provided solution (Siemens MedCom, Siemens Healthineers), and the commercial body composition analyzer (DeepCatch v1.2, Medical IP) was used for MRI and CT, respectively. The proposed deep neural network was implemented inDeepCatch (v1.x, Medical IP, South Korea).
**Results:** The R-squared value between MRI- and equation-based liver PDFF from original LDCT, deep learning-based liver PDFF from simulated LDCT without and with denoiser, and original standard dose CT were 0.6922, 0.7044, 0.8651, and 0.8664, respectively.
**Conclusion:** In this study, we proposed the deep learning method for generating liver PDFF maps from LDCT images through estimator and denoiser. The proposed method may be a promising tool for measuring liver fat content without additional MRI acquisition.

### SS 4.7

### MRI hepatic steatosis and sarcopenia in metabolic patients: a correlation prospective study

B. Masci, M. Zerunian, T. Polidori, A. Del Gaudio, D. Caruso, A. Laghi; Rome/IT

**Purpose:** To analyse the possible correlation between hepatic steatosis and sarcopenia evaluated with skeletal muscle index (SMI) at unenhanced MRI of the abdomen in patients with multiple metabolic risk factors.
**Material and Methods:** Thirty patients (nine females) with multiple metabolic risk factors were prospectively enrolled between October and December 2022. Each patient underwent 1.5T upper abdomen MRI examination; acquisition protocol included axial proton density fat fraction (PDFF), and axial T1-weighted dual-echo breath hold sequences targeted to the evaluation of lumbar paraspinal muscles at the third lumbar vertebra (L3) level. Two expert radiologists performed quantitative image analysis on a dedicated workstation. Steatosis estimation expressed as percentage was collected and grading of steatosis assigned. The presence of sarcopenia was assessed by segmenting the L3 dual-echo images with ImageJ pixel analysis software and calculating the SMI. Correlations among parameters were assessed using Spearman's coefficient of rank with a dedicated software and a p value<0.05 was considered significant.
**Results:** PDFF values of hepatic steatosis were found in 50% of patients (7.99±5.46%). SMI average values were compatible with sarcopenia in 66% of patients (37.82±8.16$cm^2/m^2$). The statistical analysis showed significant correlation between hepatic steatosis and sarcopenia (P<0.05, Rho 0.6); moreover, by stratifying the cohort for sex, the analysis showed significant better correlation between steatosis and sarcopenia in female patients (P<0.01, Rho 0.7).
**Conclusion:** Patients with multiple metabolic risk factors and hepatic steatosis quantified at MRI are correlated with lower SMI values and sarcopenia and the correlation is stronger in female patients. This method might be a non-invasive, radiation-free and repeatable method for a comprehensive metabolic patients' assessment at diagnosis and follow-up.

### SS 4.8

### Functional liver imaging score: an intraindividual comparison between gadoxetate disodium and gadobenate dimeglumine

M. Milazzo, R. Cannella, F. Agnello, G. Rancatore, G. Brancatelli; Palermo/IT

**Purpose:** The functional liver imaging score (FLIS) is a semiquantitative tool developed to estimate liver function based on the three hepatobiliary phase (HBP) features on gadoxetate disodium (Gd-EOB-DTPA) MRI (EOB-MRI). The aim of this study is to perform an intraindividual comparison between the FLIS obtained with EOB-MRI and gadobenate dimeglumine (Gd-BOPTA) MRI.
**Material and Methods:** This study included cirrhotic patients with available laboratory and clinical data, who underwent EOB-MRI and BOPTA-MRI within 6 months acquired with an HBP at 20 minutes and 2 hours, respectively. Three readers with different experience levels independently reviewed the HBP images to evaluate the FLIS based on the following features on a 0–2 scale: hepatic enhancement, biliary excretion, and portal vein signal intensity. Intraindividual differences in FLIS were assessed with the Wilcoxon signed rank-sum test and inter-reader agreement with the intraclass correlation coefficient (ICC).
**Results:** Sixty-one patients (44 males, mean age 65.6±11.4 years) were included. The total FLIS was significantly higher with EOB-MRI compared to BOPTA-MRI (median: R1, 6vs3, p<0.001; R2, 6vs3.5, p<0.001; R3, 6vs3, p<0.001), regardless of the Child–Pugh score. There was a significantly lower liver enhancement (p<0.001 for all readers), biliary excretion (p=0.001 for R1 and R2, p=0.004 for R3), and portal vein signal intensity (p<0.001 for all readers) in the HBP obtained with BOPTA-MRI compared to EOB-MRI. The inter-reader agreement of the total FLIS was excellent with an ICC 0.93 (95%CI: 0.88, 0.95) on EOB-MRI and 0.96 (95%CI: 0.94, 0.98) on BOPTA-MRI.
**Conclusion:** HBP acquired after the administration of EOB provided significantly higher FLIS compared to BOPTA.

### SS 4.9

### Contribution of functional liver imaging score obtained from gadoxetic acid-enhanced MRI to predict liver function in patients with chronic liver disease and liver cirrhosis

N. Inan Gurcan[1], I. Sever[1], A. Atasever[1], S. Yazici[1], T. Sahin[1], S. Server[1], O. Ulusoy[1], Y. Yuzer[1]; [1]Istanbul/TR

**Purpose:** To evaluate liver function in patients with liver cirrhosis and chronic liver disease using functional liver imaging scores (FLIS) obtained from gadoxetic acid-enhanced MRI and its association with clinical-laboratory parameters including indirect bilirubin, INR, albumin–bilirubin (ALBI) grade, Child–Pugh (CP), and model for end-stage liver disease (MELD) score.
**Material and Methods:** We retrospectively analyzed 173 patients (130 men, 43 women) between January 2017 and December 2022 with the following inclusion criteria: patients diagnosed with liver cirrhosis or chronic liver disease (CLD) who underwent gadoxetic acid-enhanced MRI. Patients with mechanical cholangiectasis on MRI were excluded. Three parameters on hepatobiliary phase images were evaluated for FLIS: liver parenchymal enhancement, biliary excretion, and signal intensity of the portal vein and all of which were scored on the 0–2 ordinal scale. We assessed the correlation between clinical-laboratory liver function parameters (indirect bilirubin, INR, ALBI grade, CP, and MELD score) and FLIS using Spearman rank correlation. Receiver operating characteristic (ROC) curve analysis was performed to demonstrate the ability of FLIS for the prediction of liver function.
**Results:** FLIS parameters showed strong to very strong correlation with liver function parameters. ROC curve analysis showed that FLIS ≥ 5 was the optimal cutoff for the prediction of CLD (sensitivity, 81%; specificity, 87%).
**Conclusion:** FLIS showed a strong correlation with liver function parameters; hence, it can be useful for the prediction of liver function in clinical practice.

### *SS 4.10*

### MELIF score: an automated, non-invasive measurement of liver function using MRI that outperforms standard liver function scores

C. Rio Bartulos[1], K. Senk[2], R. Bade[3], M. Schumacher[3], J. Platz[3], N. Kaiser[3], J. Woetzel[3], P. Wiggermann[1];
*[1]Braunschweig/DE, [2]Regensburg/DE, [3]Bremen/DE*

**Purpose:** Diffuse and chronic liver disease along with HCC are a global health burden. These diseases need to be monitored during their progression, which requires tools to assess liver function. Here we present a new liver function score, which is based on T1 relaxometry and calculated fully automatically by AI-based software.

**Material and Methods:** An image-based software system was developed that includes automatic image processing with elastic spatial registration, AI liver segmentation, and incorporation of patient weight and height into liver function modeling, resulting in the MELIF score. Gadolinium ethoxybenzyl-diethylenetriaminepentaacetic acid-enhanced MR images are used to calculate the MELIF score, and data from up to 195 patients were analyzed. To analyze its diagnostic potential, the MELIF score is retrospectively compared with classical liver function parameters such as MELD, Child–Pugh (CP), and ALBI scores, as well as with the T1 reduction rate (rrT1), a classical image-based liver function parameter on which the MELIF score is based. For this purpose, we use Pearson correlation and receiver operating characteristic area-under-the-curve analysis.

**Results:** The MELIF score correlates significantly stronger with MELD and CP score than ALBI or rrT1. Furthermore, we can show that the MELIF score is better able to distinguish between good and impaired liver function (AUC 0.8), defined by the MELD score, and between patients with and without cirrhosis (0.83) than the ALBI score (0.77 and 0.79).

**Conclusion:** In the future, this developed software will enable fully automated routine determination of liver function. The MELIF score can compete with common liver function parameters and perform better than the ALBI score or rrT1.

09:00 - 10:30 Room 1

*Scientific Session SS 5*

### Hepatocellular carcinoma: detection and characterisation

### SS 5.1

### The combination of non-contrast abbreviated MRI and alpha foetoprotein has high performance for HCC screening

R. Girardet[1], G. Manasseh[1], M. Dubois[1], M. Jreige[1], C. Du Pasquier[1], E. Canniff[1], M. Gulizia[1], M. Bonvin[1], Y. Aleman[1], B. Taouli[2], M. Fraga[1], C. Dromain[1], N. Vietti Violi[1]; [1]Lausanne/CH, [2]New York, NY/US

**Purpose:** To compare the diagnostic performance of two abbreviated MRI sets [non-contrast (NC)-AMRI with/without alpha foetoprotein (AFP), and dynamic (Dyn)-AMRI] to complete MRI for HCC detection in an at-risk population.

**Material and Methods:** This retrospective study included 351 patients (M/F 264/87, mean age 57 y.o.) with chronic liver disease, who underwent extracellular contrast-enhanced MRI between 2014 and 2020. Two reconstructed AMRI sets were obtained based on complete MRI: NC-AMRI [T2-weighted imaging(WI)+diffusion-WI] and Dyn-AMRI [T2-WI+dynamic T1-WI] and were assessed by 2 radiologists who reported all suspicious lesions, using LI-RADS/adapted LI-RADS classification. The reference standard was based on all available patient data. Inter-reader agreement was assessed and MRI diagnostic performance was compared to the reference standard.

**Results:** The reference standard demonstrated 83/351 HCC-positive patients (prevalence: 23.6%, median size: 22 mm, and positive MRIS: 83/631). Inter-reader agreement was substantial for all sets; k=0.68 (NC-AMRI), 0.67 (Dyn-AMRI) and 0.68 (complete MRI). Sensitivity of Dyn-AMRI and complete MRI (both 92.8%) were similar, higher than NC-AMRI (72.3%, $p<0.001$). Specificities were not different between sets. NC-AMRI+AFP (92.8%) had similar sensitivity to Dyn-AMRI and complete MRI. In patients with small-size HCCs (≤2cm), sensitivities of Dyn-AMRI (85.3%) and complete MRI (88.2%) remained similar ($p=0.564$), also outperforming NC-AMRI (52.9%, $p<0.05$). NC-AMRI+AFP had similar sensitivity (88.2%) to Dyn-AMRI and complete MRI ($p=0.706$ and $p=1$, respectively).

**Conclusion:** Dyn-AMRI has similar diagnostic performance to complete MRI for HCC detection, while both outperform NC-AMRI, especially for small-size HCCs. NC-AMRI+AFP demonstrates similar sensitivity to Dyn-AMRI and complete MRI.

### SS 5.2

### A preliminary study on the prediction of HCC in patients with chronic hepatitis B using heterogeneous data

J.-M. Kim[1], H.-J. Chung[1], D. Lee[2], J.-H. Lee[2], S. Park[1]; [1]Medical IP., Co., Ltd/KR, [2]Seoul National University College of Medicine/KR

**Purpose:** To investigate whether a CT-derived quantitative factors with deep learning technology is appropriate to improve the performance of HCC prediction in patients with chronic hepatitis B.

**Material and Methods:** The two types of HCC prediction model were developed using gradient-boosting machine (GBM) algorithm in chronic hepatitis B patients. The first model comprises 10 baseline parameters used in PLAN-B model (cirrhosis, age, platelet count, ETV/TDF, sex, serum ALT, and HBV DNA, albumin and bilirubin levels, and HbeAg status). The second model further includes two CT-derived quantitative factors, liver and spleen volumes, using commercial body composition analyzer software (DeepFore v1.x, Medical IP, South Korea). 1,375 patients with chronic hepatitis B were retrospectively included. 1100 patients (diagnosed with and without HCC ratio was 0.1455) randomly chosen in the cohort were used for training and the rest were used for internal validation. For model comparisons, the area under the receiver operating characteristics (AUROC) was used.

**Results:** The validation dataset including 42 HCC and 233 non-HCC patients was studied. The AUROC of model with only 10 baseline parameters was 0.6232 and those of model with 10 baseline parameters and volume of liver and spleen was 0.7073.

**Conclusion:** In this study, we developed and evaluated two HCC prediction models with and without using CT-derived quantitative factors. According to the results, the model with liver and spleen volumes has significantly better predictive performance than those with only 10 baseline parameters.

### SS 5.3

### Prospective randomized controlled trial of US versus US and biomarkers for early detection of HCC

H. Farhang Zangeneh, O. Cerocchi, K. Khalili, L.A. El-Karim, H. Janssen, B. Hansen, J. Feld; Toronto, ON/CA

**Purpose:** To determine the impact of triple blood biomarkers in US screening for early HCC.

**Material and Methods:** Adults with cirrhosis or high-risk HBV infection followed at one institution were randomized to HCC screening with US alone (Group A) or US+BM (Group B) with measurement of alpha-fetoprotein (AFP), lectin-reactive fraction of AFP (AFP-L3) and des-gamma-carboxy prothrombin (DCP). Elevated BM levels and/or findings on US triggered CT/MRI for confirmation of HCC diagnosis. The primary outcome was the proportion of HCCs diagnosed at a curable stage (BCLC 0/A) within Milan criteria.

**Results:** 1,208 patients were enrolled with median age 59 (18–88) years; 72% were male. Primary disease: HBV (64%) and HCV (21%). 770 patients (64%) were cirrhotic. In Group A (mean follow-up of 33 (CI95% 25.5–39.6) months, 9.2 US/pt), 35 HCCs were diagnosed, 31 (87%) in cirrhotics, of which the BCLC was 0 in 17 (49%), A in 13 (37%), B in 3 (9%) and C for 2 (6%). In Group B (mean follow-up 21 (CI95% 14.5–27.5) months, 7.9 US/pt), 27 HCCs were diagnosed, 24 (89%) in cirrhotics, of which BCLC was 0 in 9 (33%), A in 13 (48%), B in 2 (7%) and C in 3 (11%). Three HCCs were detected incidentally by other means and excluded for analysis. In Group A, 27/32 (sensitivity 84%) HCCs were diagnosed at a curable stage (BCLC 0/A), compared to 22/27 (sensitivity 81%) in group B (NS). In group B, 21 (78%) HCCs were evident on US and 9 (33%) were associated with elevated BM of which 6 (22%, 5/6 BCLC 0/A) were found by BM with a negative corresponding US.

**Conclusion:** US at a specialized center has a high sensitivity for HCC screening, diminishing impact of BMs.

Scientific Sessions

Authors' Index

### SS 5.5

### A different diagnostic criteria based on abbreviated MR imaging protocol for HCC identification in high-risk patients

F. Che, S. Bin; *Chengdu/CN*

**Purpose:** The aim of this study was to prospectively evaluate the detection performance of various MR sequences and abbreviated MRI (aMRI) protocols in different clinical settings and further compare the different imaging criteria for the diagnosis of HCC using either extracellular contrast agent (ECA) or hepatobiliary-specific contrast (HBSC) MR imaging.
**Material and Methods:** This prospective study included 247 nodules in 222 patients (mean age, 53.32 ±10.84 years; range, 22–79 years). The detection performance and imaging features of each nodule were evaluated in all MR sequences by three experienced abdominal radiologists. Detection performance of each nodule on all MR sequences was compared and further the diagnostic performance of various diagnostic criteria was evaluated.
**Results:** For those patients who underwent ECA-MRI, the conventional imaging hallmark of “AP+PVP and/or DP” was recommended, as 100% lesion detection rate and 60.19% sensitivity and 80.95% specificity. Additionally, for those patients who underwent HBSC-MRI, the diagnostic criteria of “DWI+HBP” were strongly recommended. These diagnostic criteria demonstrated both in all tumor size and for nodules ≤2 cm, higher sensitivity (93.07% and 90.16%, all p<0.05, respectively) and slightly lower specificity (64.71% and 87.50%, all p>0.05, respectively) than that of European Association for the Study of the Liver (EASL) criteria.
**Conclusion:** Different diagnostic criteria were recommended according to the lesion detection and diagnostic performance in different clinical settings. Our new diagnostic criteria demonstrated high lesion detection rate and significantly higher sensitivity, comparable specificity than that of EASL criteria for HCC with HBSC-MR.

### SS 5.6

### Diagnostic accuracy of washout at subtraction imaging for the diagnosis of HCC in spontaneous T1 hyperintense observations

J. Bizeul; *Angers/FR*

**Purpose:** Unlike arterial phase, there is no consensus regarding the use of subtraction imaging on post-arterial phase images (i.e., portal venous (PVP), delayed/transitional (DP/TP) or hepatobiliary phases (HBP)). This study aimed to assess the value of subtraction on post-arterial phase images for the non-invasive diagnosis of HCC in spontaneous T1 hyperintense nodules in cirrhotic patients.
**Material and Methods:** This study is retrospective from a prospective multicenter database that included patients from August 2014 to September 2017. All nodules with spontaneous T1 hyperintensity were included. All patients underwent extracellular agent (ECA) and hepatobiliary agent (HBA) MRI within one month. Two reading sessions were performed without and with subtraction images on post-arterial phase images.
**Results:** Forty-six nodules (including 26 HCC) in 39 patients with cirrhosis were analyzed. Using LI-RADS and ECA-MRI, the sensitivity was 63.9% (95%CI: 40.7, 82.8) without subtraction and 72.7% (95%CI: 49.8, 89.3) (p=1.000) with subtraction. The specificity was 66.7% (95%CI: 41.0, 86.7) and 33.3% (95%CI: 13.3, 59) (p=0.553, respectively).
**Conclusion:** These results suggest that subtraction images should not be used on post-arterial phase images (i.e., PVP, DP/TP and HBP) for the non-invasive diagnosis of HCC on spontaneous T1 hyperintense nodules on cirrhosis.

### SS 5.7

### Deep learning-based reconstruction-applied diffusion-weighted imaging, in comparison with conventional technique

J. Park[1], G. Seo[1], J. Sung[2], E. Lee[1], Y. Park[1]; [1]*Busan/KR*, [2]*Seoul/KR*

**Purpose:** To evaluate DWI using deep learning-based reconstruction (DL-DWI) in comparison with conventional DWI (C-DWI) in liver imaging.
**Material and Methods:** Patients who underwent gadoxetic acid-enhanced liver MRI at 3T from January to February 2022 were included in the study. Qualitative evaluation using 4-point scale for liver edge, ghosting/distortion artifact and image quality was done in both DL-DWI and C-DWI with b values of 0, 50, 400 and 800 s/mm$^2$. Among the study group, patients with imaging diagnosis of HCC were assorted for quantitative assessment. Quantitative evaluation was performed with estimated SNR (signal-to-noise ratio) and lesion-to-liver contrast ratio.
**Results:** A total of 109 patients (age 27–89, mean 63.31) were found in the study. DL-DWI presented higher score for liver edge, ghosting/distortion artifact and image quality than C-DWI in all b values (P < 0.001). In quantitative evaluation, 36 patients (age 43–81, mean 63.28) with 113 numbers of HCC showed higher SNR and lesion-to-liver contrast ratio for DL-DWI than C-DWI (P = 0.001–0.036, P = 0.045 in each).
**Conclusion:** Deep learning using DWI improved image quality and artifact with higher SNR and liver-to-lesion ratio when compared with C-DWI.

### SS 5.8

### HCC prediction using multi-sequence MR radiomics in hepatobiliary phase hypointense nodules without arterial phase hyperenhancement: a multicenter study

S. Youn, J.-I. Choi, Y. Nam, I. Joo, S. Kim, B. Park, C.-H. Lee, H. Park; *Seoul/KR*

**Purpose:** To develop a radiomics prediction model using multi-sequence MRI to predict HCC in hepatobiliary phase (HBP) hypointense nodules without arterial phase hyperenhancement (APHE)
**Material and Methods:** This retrospective multicenter study included patients with pathologically confirmed HBP hypointense nodule without APHE who were screened with gadoxetic acid-enhanced liver MRI between January 2008 and June 2016 in 8 hospitals. Blinded central review for the pathology was performed for the diagnosis of HCC and non-malignant cirrhosis-associated nodules. We used T2-weighted images, T1-weighted images, HBPs and apparent diffusion coefficient maps. Totally 420 radiomic features were extracted from each volume of interest on these four key sequences. In training set, a per-lesion HCC classification model was developed by support vector machine (SVM). Fivefold cross validation was done. Radiomic model testing with external validation was done using area under the receiver operating characteristics (AUC).
**Results:** A total of 278 patients with 291 lesions were finally included in this study. There were 199 HCCs and 92 nonmalignant cirrhosis-associated nodules. 221 lesions from 6 hospitals were included in the training set and 70 lesions from 2 hospitals were included in the validation set. In the training set, mean AUC to predict HCC was 0.80 with a range from 0.74 to 0.89. In validation, AUC was 0.66, which is relatively lower than the performance of the training set.
**Conclusion:** A radiomics model based on multi-sequence MRI serves as a helpful quantitative approach for distinguishing HCC from non-malignant cirrhotic nodule in HBP hypointense nodules without APHE.

### SS 5.9

### Application of liver imaging reporting and data system standardised reporting recommendations on CT and MRI radiology reports in clinical practice

M. Milazzo, F. Sorrentino, R. Cannella, G. Brancatelli; *Palermo/IT*

**Purpose:** The liver imaging reporting and data system (LI-RADS) encourages the use of standardised reporting and defines requirements to improve consistency and reporting standards. This study evaluates the application of LI-RADS reporting recommendations in CT and MRI reports from clinical practice.

**Material and Methods:** This retrospective study included radiology reports obtained between January 2018 and May 2022 using the LI-RADS algorithm. Reports were reviewed to assess the timing (during day or nightshift), priority (ordinary or urgent), experience of the radiologists (subspecialised abdominal radiologists or general radiologists), and categorisation of the observations (described individually or in aggregate). The description of location, size, major and ancillary features of the observations, other hepatic or extrahepatic findings, and impression with final observation diagnosis or management recommendations were recorded and compared using the Pearson $\chi^2$ or Fisher's exact test.

**Results:** A total of 973 reports provided by 28 different radiologists were included (720 reports from subspecialised abdominal radiologists and 253 from general radiologists). According to the highest category, the LR-5, LR-M, and LR-TIV were described in 247 (25.4%), 31 (3.2%), and 53 (5.4%) reports, respectively. Reports from subspecialised abdominal radiologists more frequently provided ancillary features (27.0% vs. 9.9%; p<0.001) and impression with detailed diagnosis or management recommendations (67.5% vs. 57.7%; p=0.005), while reports from general radiologists more frequently described hepatic vascular findings (96.8% vs. 91.7%; p=0.006) and extrahepatic findings (99.2% vs. 94.7%; p=0.002). No differences were observed according to the timing or priority of the reports.

**Conclusion:** Findings and impression reporting are affected by the radiologists' experience in liver imaging, with general radiologists less commonly reporting ancillary features and final diagnosis or management recommendations in the impressions.

### SS 5.10

### Liver imaging reporting and data system version 2018 for HCC <1.0 cm on MRI

H. Jang, S. Choi; *Seoul/KR*

**Purpose:** We aimed to develop and evaluate a modified liver imaging reporting and data system (LI-RADS) version 2018 using significant ancillary features for diagnosing HCC <1.0 cm on gadoxetate disodium-enhanced MRI.

**Material and Methods:** Patients who underwent preoperative gadoxetate disodium-enhanced MRI for focal solid nodules <2.0 cm within 1 month of MRI between January 2016 and December 2020 were retrospectively analyzed. Major and ancillary features were compared between HCCs of <1.0 cm and 1.0–1.9 cm using the chi-square test. Significant ancillary features associated with HCC <1.0 cm were determined by univariable and multivariable logistic regression analysis. The sensitivity and specificity of LR-5 were compared between LI-RADS v2018 and our modified LI-RADS (applying the significant ancillary feature) using generalized estimating equations.

**Results:** Of 796 included nodules, 248 were <1.0 cm and 548 were 1.0–1.9 cm. HCC <1.0 cm less frequently showed an enhancing capsule (7.1% vs. 31.1%, p<.001) and threshold growth (0% vs. 8.3%, p=.007) than HCC of 1.0–1.9 cm. Restricted diffusion was the only ancillary feature significant for diagnosing HCC <1.0 cm (adjusted odds ratio = 11.50, p<.001). In the diagnosis of HCC, our modified LI-RADS using restricted diffusion had significantly higher sensitivity than LI-RADS v2018 (61.8% vs. 53.5%, p<.001), with similar specificity (97.3% vs. 97.8%, p=.157).

**Conclusion:** Restricted diffusion was the only significant independent ancillary feature for diagnosing HCC <1.0 cm. Our modified LI-RADS using restricted diffusion can improve the sensitivity for HCC <1.0 cm.

**09:00 - 10:30** | **Room 3**

*Scientific Session SS 6*

### Inflammatory bowel disease

### SS 6.1

### Diagnostic performance of sonographic activity scores for adult terminal ileal Crohn's disease compared to MR and histological reference standards: experience from the METRIC trial

S. Kumar[1], T. Parry[1], S. Mallett[1], A. Plumb[1], G. Bhatnagar[1], R. Beable[2], M. Betts[3], G. Duncan[4], A. Gupta[1], A. Higginson[2], R. Hyland[5], R. Lapham[5], U. Patel[1], J. Pilcher[1], A. Slater[4], D. Tolan[5], I. Zealley[3], S. Halligan[1], S. Taylor[1], METRIC study Investigators[1]; *[1]London/GB, [2]Portsmouth/GB, [3]Dundee/GB, [4]Oxford/GB, [5]Leeds/GB*

**Purpose:** The simple ultrasound activity score for Crohn's disease (SUS-CD) and bowel ultrasound score (BUSS) are promising intestinal US (IUS) indices of CD but studied mainly in small settings with few sonographers. We compared SUS-CD and BUSS against histological and MR enterography (MRE) reference standards in a post hoc analysis of a prospective multicentre, multireader trial.

**Material and Methods:** Participants recruited to the METRIC trial (ISRCTN03982913) were studied, including those with available terminal ileal (TI) biopsies. Sensitivity and specificity of SUS-CD and BUSS for TI CD activity were calculated with 95% confidence intervals (CI), from the prospective observations of the original METRIC trial sonographers against the histological activity index (HAI) and the simplified magnetic resonance index of activity (sMARIA).

**Results:** We included 284 patients (median 31.5 years, IQR 23–46) from 8 centres, who underwent IUS and MRE. Of these, 111 patients had available terminal ileal biopsies with HAI scoring. Against histology, sensitivity and specificity for active disease were 79% (95% CI 69–86%) and 50% (31–69%) for SUS-CD, and 66% (56–75%) and 68% (47–84%) for BUSS, respectively. Compared to sMARIA, the sensitivity and specificity for active CD were 81% (74–86%) and 75% (66–83%) for SUS-CD, and 68% (61–74%) and 85% (76–91%) for BUSS, respectively. The sensitivity of SUS-CD was significantly greater than BUSS against HAI and sMARIA (p<0.001), but its specificity was significantly lower than BUSS against the MRE reference standard (p=0.003).

**Conclusion:** Particularly when compared to MRE activity scoring, SUS-CD and BUSS are promising tools in a real-world clinical setting.

### SS 6.2

### Relationship between MR enterography findings and Crohn's disease-related outcomes

J. Rimola[1], A. Fernàndez-Clotet[1], N. Capozzi[2], J. Panes[1], E. Ricart[1], I. Ordas[1]; [1]Barcelona/ES, [2]Bologna/IT

**Purpose:** The value of MR enterography (MRE) for assessing the efficacy of therapeutic interventions in Crohn's disease (CD) is limited by our lack of understanding of the relationship between disease activity measured by MRE and clinical outcomes. The objective is to determine the predictive value of MRE findings 46 weeks after initiating biologic therapy for clinically relevant CD-related outcomes in patients with CD.

**Material and Methods:** This is a longitudinal and single-center observational prospective study conducted between 2010 and 2019. We included patients with CD starting a biologic drug evaluated by MRE pre- and at week 46 post-treatment with a minimum post-MRE follow-up of 2 years. MRE was performed using standard MRE protocol. Assessment of inflammation and/or complications was done in segment-by-segment basis including calculation of the MaRIA score.

**Results:** 89 patients with a median follow-up of 53 months (IQR 44–76) were included. At inclusion, 31 (34.8%) had an inflammatory phenotype, 27 (30.3%) stenosing, 9 (10.1%) fistulizing, and 22 (24.7%) stenosing/fistulizing. During follow-up, 25 (28.1%) required surgery, 17 (19.1%) hospitalization, 40 (44.9%) had a clinical recurrence (CDAI >150), and 12 (13.5%) underwent endoscopic dilation. MaRIA score ≥11 in any intestinal segment at week 46 was associated with increased risk of surgery [HR 6.28 (1.42–27.84), p=0.02] or surgery/endoscopic dilation [HR 3.76 (1.24–11.38), p =0.02] during follow-up. Increased wall contrast enhancement was associated with a higher risk of clinical recurrence [HR 1.01 (1.00–1.02), p=0.03].

**Conclusion:** MRE findings at week 46 after initiating biologic therapy predict long-term clinical outcomes in CD patients. The main determinants of poor prognosis are a MaRIA score ≥11 and mural enhancement.

### SS 6.3

### Single-breath-hold single-shot fast spin echo of MR enterography in Crohn's disease: impact of deep learning-based reconstruction

E. Park, Y. Lee, J.-H. Son, J.-H. Yoon; Busan/KR

**Purpose:** To compare the image quality of single-breath-hold (SBH) single-shot fast spin echo (SSFSE) and multiple BH (MBH) SSFSE with and without deep learning-based reconstruction (DLR) in patients with Crohn's disease (CD).

**Material and Methods:** This study included 61 patients (mean age 32.9 ± 1.50 years) who underwent MR enterography for CD. The following T2-weighted coronal images were compared: single BH SSFSE with DLR (SBH-DLR) and without DLR (SBH-CR), multiple BH SSFSE with DLR (MBH-DLR) and without DLR (MBH-CR). Two radiologists independently reviewed overall image quality, artifacts, sharpness, and motion-related signal loss using a 5-point scale. Parameters suggesting active inflammation (mural thickness, mural T2 intensity, perimural T2 intensity) were evaluated in three bowel segments (ileum, terminal ileum, ascending colon). Presence of spatial misalignment of slices was evaluated. Signal to noise levels (SNR) were calculated in two different locations of each sequence.

**Results:** DLR statistically significantly improved image quality, artifact, and sharpness of SBH but not MBH. The MBH-CR and SBH-DLR showed no significant difference in scores. SBH-DLR showed highest SNR ($p < .01$). Inter-reader agreement for inflammatory features was good to excellent in all bowel segments ($\kappa$ = 0.77-0.95) and inter-sequence agreement was nearly perfect ($\kappa$ = 0.92-0.94). Misalignment artifacts were observed more in MBH than SBH images ($p < .01$).

**Conclusion:** SBH-DLR demonstrated nearly equivalent quality and performance for evaluating bowel inflammation compared to MBH-CR. Furthermore, it can be acquired in less than half of the scan time, without exhausting multiple BHs and reducing slice misalignment.

### SS 6.4

### A simple scoring system using MR enterography based on the most inflamed segment to evaluate disease activity in Crohn's disease

N. Seo, J. Lim, S.-S. Kim, H. Bae, J. Cheon; Seoul/KR

**Purpose:** Routine use of MR enterography (MRE)-based indices in Crohn's disease (CD) is limited in clinical practice due to its complexity. We aimed to validate a simple MRE-based scoring system based on the most inflamed bowel segment to evaluate disease activity in adult CD patients.

**Material and Methods:** In this retrospective study, including 252 CD patients, three abdominal radiologists assessed the simplified magnetic resonance index of activity (MaRIAs) score using MRE. The maximal segmental MARIAs was defined as the largest segmental MARIAs among six bowel segments within a patient. The global MARIAs results from the sum of the each segmental MARIAs. The correlation analysis was performed between global and maximal segmental MARIAs. For patients with endoscopic results, correlation analysis was performed between the Simple Endoscopic Score for CD (SES-CD) and MARIAs. The diagnostic performance of MARIAs to predict endoscopic remission (SES-CD<3) was evaluated using the receiver operating characteristic (ROC) curve analysis.

**Results:** Global MARIAs and maximal segmental MARIAs showed strong correlation ($\rho$=0.954 [95% CI, 0.941–0.964]). In 77 patients with endoscopic results, global MARIAs and maximal segmental MARIAs showed moderate correlation with SES-CD ($\rho$=0.685 and $\rho$=0.634) without significant difference (P = 0.168). The area under the ROC curve of global MARIAs and maximal segmental MARIAs to predict endoscopic remission was 0.850 and 0.843, without significant difference (P = 0.733).

**Conclusion:** The maximal segmental MARIAs based on the most inflamed bowel segment can be a rapid and practical MRE-based index to represent overall disease activity and to predict endoscopic remission in CD.

### SS 6.5

### MRI factors associated with relapse following discontinuation of biologic medication in Crohn's disease

J. Pakpoor, H. Fitzke, M. Hameed, T. Glover, J. Holmes, W. Blad, L. Whitley, S. Taylor; London/GB

**Purpose:** The decision to stop biologic medication in Crohn's disease (CD) in apparent clinical remission is challenging. We investigated MR enterography (MRE) observations associated with future relapse.

**Material and Methods:** 49 CD patients discontinuing biologics due to clinical remission and undergoing MRE within 12 months prior, or 1 month after, were identified. Two blinded radiologists assessed small bowel disease presence/activity and determined length-of-disease, wall-thickness, fat-wrapping, and s-MARIA. Relapse status was assigned based on clinical records including patient symptoms, biochemistry, imaging and medications. Groups were compared with Chi-square and Wilson–Cox statistics.

**Results:** Patient mean age was 30.1 (SD: 13.0, 19 female). Median follow-up was 55 months (6–110). Twenty-five (51%) had disease relapse (DR) and 24 (49%) no relapse (NDR). Sixteen/24 (67%) and 15/25 (60%) of the NDR and DR, respectively, still had small bowel disease present on MRE prior to biologic cessation. This was subjectively deemed active in 10/15 (66%) DR vs just 5/16 (31%) of NDR; however, there was no significant difference in median global s-MARIA score (2 [DR] versus 1 [NDR]), p=0.14 or presence of fat wrapping (in 9 [DR] versus 4 [NR]), P=0.14. However, median wall thickness was greater in DR (7.4mm [IQR: 6.0–9.6]) vs. NDR (5.4mm [3.4–6.6]), p=0.01.

**Conclusion:** Presence or absence of residual small bowel abnormality does not in itself predict future relapse. However, in those with residual disease, bowel wall thickness is greater in those who relapse and may be a simple tool to guide the decision to discontinue biologic medications.

### SS 6.6

### Replacing IV gadolinium with quantified small bowel motility does not affect diagnostic accuracy for active small bowel Crohn's disease: a multi-reader study

H.E. Fitzke[1], J. Holmes[1], T. Parry[1], A. Patel[1], H. Lambie[2], E. Mainta[1], R. Greenhalgh[3], I. Zealley[4], D. Tolan[2], C. Robinson[5], N. Patel[1], J. Barber[1], S. Ballantyne[6], S. Upponi[7], A. Higginson[8], R. Hyland[2], H. Rafiee[1], P. Lung[1], G. Bhatnagar[9], S. Mallett[1], S. Taylor[1]; *[1]London/GB, [2]Leeds/GB, [3]Middlesex/GB, [4]Dundee/GB, [5]Reading/GB, [6]Glasgow/GB, [7]Cambridge/GB, [8]Portsmouth/GB, [9]Frimley/GB*

**Purpose:** The impact of replacing IV gadolinium (IV Gad) with a quantified small bowel motility index (MI) (Plumb et al. 2015) in MR enterography (MRE) protocols is not known. We compared the diagnostic accuracy, inter-reader agreement for small bowel Crohn's disease (SBCD) activity and identification of most severely affected segment (mSS) between MRE + IV Gad and MRE + MI.
**Material and Methods:** Patients with SBCD were identified from the METRIC trial (Taylor et al. 2018) and MRE scans with either IV Gad or MI imaging were interpreted by 16 radiologists in a cross-over design. Readers were blinded to all clinical information and scored diagnostic confidence for overall SBCD as normal (inactive); equivocal, or abnormal (active). MI was reported for the mSS in arbitrary units (a.u.) using commercially available software (GIQuant®, Motilent, London).
**Results:** 80 patients were included (54% female; 41% aged 15–25 years) of which 64 (80%) had active SBCD according to the reference standard. There was no significant difference in sensitivity or specificity for active SBCD between MRE + IV Gad and MRE + MI. The inter-rater agreement for SBCD activity and most severely affected segment was not significantly different between the two reading paradigms. The mean difference in MI between readers was 7 a.u. (95% LoA −155, 275) or 5% (95% LoA −71, 80).
**Conclusion:** Replacing IV Gad with MI did not affect the diagnostic accuracy or inter-reader agreement for the detection of SBCD activity or most severe segment.

### SS 6.7

### Motility in small bowel strictures in Crohn's disease measured with cine-MRI

K.J. Beek, K. Van Rijn, C. de Jonge, F. de Voogd, C. Buskens, G. D'Haens, K. Gecse, J. Stoker; *Amsterdam/NL*

**Purpose:** To investigate correlations between small bowel (SB) stricture motility measured with cine-MRI, and disease duration and Harvey–Bradshaw Index (HBI), respectively.
**Material and Methods:** CD patients (>18yrs) with SB strictures were included. Patients fasted 4 hours, after which they drank 1600 mL (1.9% mannitol solution) in 60 minutes prior to 3T MRI. All underwent coronal dynamic 2D-bFFE scan positioned at the most stenotic SB and the pre-stenotic dilation, during a 20-second expiration breath-hold. Bowel motility was assessed with a validated displacement mapping technique (GIQuant, Entrolytics, Motilent, UK). Strictures (wall thickening >3mm and >50% luminal reduction) and pre-stenotic dilations (luminal diameter >3cm) were delineated on a reference image and motility was quantified within these regions of interest (ROI) on a motility map, producing a single, numerical motility score (arbitrary units=AU). Stricture and pre-stenotic dilation motility scores are presented in medians [IQR]. Correlations were tested between stricture motility, disease duration and HBI by means of spearman's rank correlation test.
**Results:** Twenty-two patients (55% male, age 37yrs [IQR 25–55], disease duration 7yrs [IQR 4–12]) were included. SB stricture motility was 66AU [IQR 58–79]. Pre-stenotic dilation motility (n=6) was 150AU [IQR 102–381]. Disease duration and stricture motility showed no correlation (r=0.2, p=0.5). HBI and stricture motility were inversely correlated (r=−0.5, p<0.05).
**Conclusion:** An inverse correlation between SB stricture motility and HBI was found. This result suggests that lower motility is associated with poorer clinical condition. This finding can possibly lead to earlier endoscopic or surgical intervention. No correlation was found between SB stricture motility and disease duration.

### SS 6.8

### Cine-MRI assessment of colonic motility in patients with ulcerative colitis-associated constipation

C. Miller, J. Pakpoor, H. Fitzke, A. Emmanuel, N. Zarate-Lopez, A. Menys, W. Blad, J. Holmes, S. Bloom, S. Taylor; *London/GB*

**Purpose:** Ulcerative colitis-associated constipation (UCAC) can cause significant morbidity in UC patients but is incompletely understood and treated. Using a novel cine-MRI technique, we aimed to investigate regional colonic motility in active UC with UCAC.
**Material and Methods:** Thirty prospectively recruited participants were split into 3 groups: 10 healthy controls, 10 patients with active UC with UCAC (faecal calprotectin > 250 or an endoscopic Mayo score >1), and 10 patients with active UC without UCAC. UCAC subjects met American Gastroenterology Association constipation criteria. Subjects underwent a 12-minute cine-MRI scan after consuming 1.4 litres of 2% mannitol. Using manual ROIs, regional colonic motility indices (MI) were derived using GI-Quant® (Motilent) and compared across the 3 groups using Kruskal–Wallis and Mann–Whitney U statistics.
**Results:** Mean age was 33.4 [22–43]. Mean disease duration was 8.6 years (UCAC) and 5.7 years (UC without UCAC). Mean ascending colon MI was lower in UC with UCAC (392.8 [352.9–437.4]) compared to UC without UCAC (424.7 [330–664.3]) and healthy controls (431.4 [341.5–577.8]), although there was no significant difference between groups. The same trend of reduced MI in patients with UCAC compared to controls was also seen in the transverse colon (TC) (394 [345.9–452.8] vs 467.3 [369.9–562.6], respectively) (p=0.06), and compared to UC without UCAC (424.3 [315.2–538.6]).
**Conclusion:** In this proof-of-concept study, motility is potentially reduced in the ascending and transverse colon in patients with left-sided UC and symptoms of UCAC compared to controls, justifying study in larger cohorts. Hypomotility may be a contributing factor in UCAC suggesting stimulants/prokinetics may be effective.

### SS 6.9

### Quantified small bowel motility as a biomarker of Parkinson's disease: a feasibility study using an abbreviated MR enterography protocol

J. Holmes, H. Fitzke, J. Pakpoor, A. Menys, D. Atkinson, G. Bhatnagar, S. Taylor; *London/GB*

**Purpose:** GI dysmotility is an early feature of Parkinson's disease (PD), preceding diagnosis by >10 years (Schapira et al. 2017). We assessed the feasibility of an abbreviated MR enterography protocol (MRE) with reduced oral contrast volume to quantify global small bowel motility in PD and compared to healthy volunteers.
**Material and Methods:** PD patients suspended motility-influencing drugs 24 hrs before their scan (REC #: 11/LO/1634). They sipped 800 mL 2% mannitol over 45 mins before successive 2D dynamic sequences were acquired in the supine position. Images were processed using a commercially available non-rigid registration algorithm (GIQuant, Motilent, UK) to quantify small bowel motility. Mean ± S.D motility (arbitrary units, a.u) was compared using a t test against healthy volunteers (HV) scanned using a similar protocol—1L of 1.7% mannitol in the prone position.
**Results:** Nine PD patients (aged 53–79, 100% male) were scanned: 7 completed the 800 mL drink; oral contrast had reached the terminal ileum or beyond in 6; and median (Q1–Q3) scan duration was 15 (13–20) mins. Global small bowel motility in PD patients was 245 ± 61 a.u. compared to 331 ± 65 a.u. in six HVs (ages 24–33, 16% female p = 0.028).
**Conclusion:** The abbreviated MRE protocol was feasible in most PD patients. Small bowel motility may be reduced compared to HVs. Future work will focus on minimising acquisition time and oral contrast load and comparing age-matched controls to PD patients at different stages of disease to assess whether differences are a function of age or disease progression.

### *SS 6.10*

### MAGNIFI-CD index is appropriate for treatment monitoring in perianal fistulizing Crohn's disease

K.J. Beek, L. Mulders, K. Van Rijn, J. Tielbeek, K. Horsthuis, C. Buskens, G. D'Haens, K. Gecse, J. Stoker; *Amsterdam/NL*

**Purpose:** To validate the MAGNIFI-CD index for evaluating treatment response in perianal fistulizing Crohn's disease (pfCD).

**Material and Methods:** Patients with complex pfCD had pelvic MRI before and 3–24 months after medical and/or surgical therapy. Two independent, blinded abdominal radiologists randomly scored MAGNIFI-CD and separate items. Two clinicians scored clinical outcomes (remission, response, non-response) using "fistula drainage assessment" (FDA). Responsiveness (FDA; Wilcoxon signed rank test), interobserver agreement (ICCs or weighted K) and test accuracy (ROC follow-up (FU) MAGNIFI-CD) were determined. Youden indices were used for optimal cut-off values for FDA categories.

**Results:** Sixty-seven patients (median age 30 [IQR 23–47], 51% female) with mean CD and pfCD duration of 7.0 yrs (SD 8.7) and 3.7 yrs (SD 4.3), respectively. Baseline MAGNIFI-CD was 18 [IQR 9–20]. Significant decrease in MAGNIFI-CD was observed in responders and remitters ($p=0.005/p<0.001$); a non-significant decrease ($p=0.22$) in non-responders. MAGNIFI-CD had almost perfect ICC (0.88 (95%CI 0.78–0.92)); reliability of the separate items was moderate to substantial. For response, AUCs of improvement and relative improvement were 0.75 ($p=0.002$) and 0.77 ($p=0.001$), resulting in optimal cutoffs of ≥2 (sens/spec: 77/65%) and ≥25% (sens/spec: 64/82%). For remission, AUCs of FU MAGNIFI-CD and relative improvement were 0.81 ($p<0.0001$) and 0.72 ($p=0.002$), resulting in optimal cutoffs of ≤6 (sens/spec: 45/90%) and ≥50% (sens/spec: 49/94%).

**Conclusion:** The MAGNIFI-CD index is suitable for treatment monitoring as it has an almost perfect agreement, robust responsiveness to clinical change and an acceptable to excellent test accuracy to determine response or remission (according to the FDA). Given the moderate agreement, the item "dominant feature" would need additional training or better definition.

### *SS 6.11*

### A clinical/radiological risk assessment model can accurately predict perianal fistula healing in patients with Crohn's disease: a multicenter cohort study

J. Munir[1], J. McCurdy[2], S. Parlow[2], J. Reid[3], R. Yanofsky[4], T. Alenzi[4], J. Meserve[5], B. Becker[6], R. Mallick[2], T. Ramsay[2], G. Rosenfeld[3], T. Bessisow[4], V. Jairath[7], S. Singh[5], D. Bruining[6], B. Macdonald[2]; *[1]Muscat/OM, [2]Ottawa, ON/CA, [3]British Columbia, BC/CA, [4]Montreal, QC/CA, [5]San Diego, CA/US, [6]Florida, FL/US, [7]London, ON/CA*

**Purpose:** To identify clinical and radiologic variables associated with PAF (perianal fistula) healing in Crohn's disease (CD) and create risk assessment model (RAM) to predict healing after anti-TNF therapy.

**Material and Methods:** This is a multicenter retrospective study that included biologically naïve CD adults who initiated therapy for active PAF after MRI pelvis. MRI studies were prospectively re-evaluated by radiologists blinded to clinical outcomes. Variables with clinical healing at 6 months were determined by univariable and multivariable logistic regression. Variables with p value <0.1 on univariable analysis were included in full model and simplified version consisting of MRI variables alone. Model performance was determined by c-statistic and internally validated using bootstrap methods. Performance of simplified model was tested against MAGNIFI-CD and modified Van Assche scores (mVAS) based on area under receiver operating characteristics (ROC) curves using De Long test. Aggregate scores from weighted variables from simplified model were divided to predict likelihood of healing.

**Results:** In total, 216 patients: 132 (61%) males; mean age 32yrs (SD, 12), healing in 116 (54%). Adjusted model included age at diagnosis, time to therapy initiation, dominant fistula length, multiple branched tracts, external openings, complex fistulas, tract T2 hyperintensity, horseshoe anatomy and collections>1.3cm. Adjusted full model and simplified version were accurate in predicting healing (c-statistic, 0.75; 95%CI (0.68–0.81) and 0.72; 95%CI (0.65–0.79), respectively). Simplified model performed better at predicting healing than MAGNIFI-CD (AUC, 0.6087; 95%CI, 0.53–0.69; $p=0.0018$) and mVAS (AUC, 0.5787; 95%CI, 0.50–0.66; $p=0.0001$). Aggregate scores were between 0 and 75, 76 and 175 and >175 from simplified model with low (<27%), moderate (27–65%) and high (>65%) rates of healing.

**Conclusion:** We developed RAMs consisting of clinical and/or radiologic factors for predicting PAF healing in CD patients, which may have important implications in clinical study design and to help set realistic expectations of healing for patients and providers.

Scientific Sessions

Authors' Index

**11:00 - 12:30** **Room 1**

*Scientific Session SS 7*

### Hepatocellular carcinoma: prognosis

### SS 7.1

### Imaging and prognostic characterization of fat containing HCC subtypes

A. Faure[1], M. Dioguardi Burgio[1], R. Cannella[2], R. Sartoris[1], M. Bouattour[1], F. Cauchy[1], A. Beaufrère[1], V. Vilgrain[1], M. Ronot[1]; [1]Clichy/FR, [2]Palermo/IT

**Purpose:** Steatohepatitic HCC (sh-HCC) is characterized by >50% area of steatohepatitic changes. However, fat (+/- inflammation) can be found in non-otherwise specified HCC (NOS-HCC). We compared the imaging features and outcomes of sh-HCC, fatty NOS-HCC, and NOS-HCC with a steatohepatitic component <50% (sh-NOS-HCC).
**Material and Methods:** Patients undergoing hepatic resection for an HCC with fat content on pathology between 2012 and 2021 were included. Imaging features were assessed per liver imaging reporting and data system (LI-RADS) v2018 (features and categories). Fat quantification was performed on chemical-shift MRI. Tumor groups were compared, and recurrence-free (RFS) and overall survival (OS) were estimated.
**Results:** 94 patients were included (65 men, 80%; median 66 yrs (60–71)), including 21 (26%) with nonalcoholic steatohepatitis (NASH). There were 43 sh-HCC (46%), 35 sh-NOS-HCC (37%) and 16 fatty NOS-HCC (17%). The median tumoral fat fraction was 8%, with significant differences between sh-HCC and sh-NOS-HCC (9.5% vs 5% p=0.03), but not otherwise. NASH was associated with sh-HCC (p=0.015). There was no difference regarding LI-RADS major features or categories between groups, and most tumors were classified as LR-4–5 in high-risk patients. A mosaic appearance on MRI was more frequent in sh-NOS-HCC (p=0.01) and fat-in-mass on CT more frequent in sh-HCC (p=0.009). After a median follow-up of 108 months, the median RFS was 58 months (95%CI 35–81) with no difference between groups (p=0.181). The median OS was 87 months (95%CI 74–101), with no difference between groups (p=0.69).
**Conclusion:** Imaging and prognostic characterization of fat containing HCC subtypes largely overlap. A steatohepatitic HCC may be suspected in homogenous tumors with fat-in-mass and developed on NASH.

### SS 7.2

### Imaging characteristics of HCC according to prothrombin induced by vitamin K absence-II and alpha-fetoprotein serum levels

C. du Boisbaudry[1], M. Dioguardi Burgio[1], R. Cannella[2], A. Payancé[1], H. Manceau[1], K. Peoch[1], V. Chicha Cattoir[1], M. Bouattour[1], V. Paradis[1], V. Vilgrain[1], M. Ronot[1]; [1]Clichy/FR, [2]Palermo/IT

**Purpose:** To assess the imaging characteristics of treatment-naive HCC according to alpha-fetoprotein (AFP) and prothrombin induced by vitamin K absence-II (PIVKA-II) serum levels.
**Material and Methods:** Patients with all-stage HCC treated between 2016 and 2019 with both AFP and PIVKA-II serum level were included. CT and MRI features were described per liver imaging reporting and data system v2018 lexicon (features and categories). Various biomarker thresholds were tested and compared (chi$^2$ and Fisher tests).
**Results:** 133 patients were included (114 men (86%); median 66 yrs (59–73), 122 (92%) with a chronic liver disease). 129 and 89 underwent CT and MRI. HCC were BCLC A, B, C and D in 44 (33%), 38 (29%), 43 (32%) and 8 (6%). Median PIVKA-II level was 450 mAU/mL (IQR 120–3491.5) and median AFP level was 9.7 ng/mL (IQR 5–80.5 ng/mL). Several features, including portal vein tumor-in-vein (TIV), LR-M, tumor necrosis and infiltrative growth pattern were associated with both AFP and PIVKA-II increase. Some features were associated with PIVKA-II increase only, such as hepatic vein TIV [100% and 88% when PIVKA-II >200 mAU/mL (p=0.039) or >1000 mAU/mL (p=0.003), respectively] or biliary dilatation (100% when PIVKA-II >200 mAU/mL, p=0.014). Some were associated with AFP increase only, such as metastatic lymph node, extrahepatic metastases, and multifocal tumors.
**Conclusion:** The pattern of imaging features of HCC appears different according to AFP and PIVKA-II serum levels. An increase in PIVKA-II seems more representative of local tumor aggressiveness, while an increase in AFP may be more informative about distant or extrahepatic tumor spread. Both dosages are complementary and should be done.

### SS 7.3

### Prognostic value of MRI features in patients with single large HCC after curative resection

K. Gu, J. Min; Seoul/KR

**Purpose:** To investigate the prognostic value of preoperative MRI findings regarding treatment outcomes in patients with single large HCC (> 8 cm) after curative surgical resection.
**Material and Methods:** This retrospective study included 154 patients (mean age, 61 years, 128 men) with single large HCC who underwent gadoxetic acid-enhanced MRI and surgical resection between March 2008 and January 2020. Clinical variables including tumor marker and albumin–bilirubin (ALBI) grade and imaging features including tumor size, tumor margin and proportion of hypovascular component on hepatic arterial phase (HAP) > 50% were evaluated. The overall survival (OS), recurrence-free survival (RFS), and the associated factors were evaluated using the Cox proportion hazard model.
**Results:** During a median follow-up of 50.0 months, 90 (58.4%) patients showed recurrence and 63 (14.5%) patients died. Median time to recurrence and OS were 15.4 months and 50.0 months, respectively. On the multivariable analysis, ALBI grade 2-3 (hazard ratio [HR] 2.13, p=0.033) and hypovascular component on HAP > 50% (HR 2.17, p=0.030) were predictors of poorer RFS. ALBI grade 2-3 (HR 2.41, p=0.016), hypovascular component on HAP > 50% (HR 2.31, p=0.021), serum AFP level > 400 ng/ml (HR 2.33, p=0.020) and non-smooth tumor margin (HR 1.99, p=0.047) were predictors of poorer OS.
**Conclusion:** The preoperative MRI features, such as hypovascular component on HAP > 50% and non-smooth tumor margin, are useful in predicting poorer treatment outcomes of HCC after surgical resection. Clinical treatment decisions for large HCC should be made carefully even though the tumor is solitary.

### SS 7.5

### Preoperative CT features associated with a textbook outcome in patients with HCC undergoing hepatic resection

M. Baudouin[1], R. Sartoris[2], F. Cauchy[2], C. Hobeika[2], V. Vilgrain[2], M. Ronot[2]; [1]Limoges/FR, [2]Clichy/FR

**Purpose:** A textbook outcome (TO) is a composite indicator covering an entire intervention process. The ultimate goal of TO is to reflect the "ideal" intervention. The aim was to identify preoperative CT features associated with TO in patients with HCC undergoing hepatic resection

**Material and Methods:** Retrospective study included HCC patients treated by partial hepatic resection between 2012 and 2019. Preoperative CT was reviewed to collect and compute 74 qualitative and quantitative features covering the hepatic, cardiovascular, pulmonary, musculoskeletal, pancreatic, and kidney systems and anthropometrical indices. Characteristics associated with TO with a p value <0.2 were fitted in multivariate models, including clinical data and imaging features. Models were compared with the Akaike information criteria (AIC), and the areas under the ROC curves.

**Results:** 254 patients were analyzed (78% men, median 64.0 yrs [54.2–70.0]), and 76 (30%) had a TO. Univariate analysis identified coronary calcifications, lung atelectasis, volume of the future liver remnant, dysmorphic liver, tumor size, size of the common bile duct, and paravertebral muscle index as associated with TO. The best clinico-radiological model had an AUC of 0.86 (AIC of 325) for the prediction of TO. Simplified predictive models, including 13, 9, and 6 imaging features, were fitted with AUCs of 0.83, 0.81, and 0.80 and AICs of 260, 260, and 263, respectively. Models were well calibrated. A nomogram was derived from the 9-variable model (https://chctoscantopredicttbo.shinyapps.io/dynnomapp).

**Conclusion:** Several preoperative CT features are associated with a TO in patients undergoing resection for an HCC. Considering clinical and imaging features may help refine the peri-operative management of patients.

### SS 7.6

### Performances and variability of CT radiomics for the prediction of microvascular invasion and survival in patients with HCC: a matter of chance or standardization?

R. Cannella[1], J. Santinha[2], A. Bèaufrere[3], M. Ronot[3], F. Cauchy[3], C. Matos[2], N. Papanikolaou[2], V. Vilgrain[3], M. Dioguardi Burgio[3]; [1]Palermo/IT, [2]Lisbon/PT, [3]Clichy/FR

**Purpose:** To assess the performance and variability of a radiomics-based model for the prediction of microvascular invasion (MVI) and survival in patients with resected HCC, simulating its sequential development and application.

**Material and Methods:** This study included 230 patients with 242 surgically resected HCCs who underwent preoperative CT, of which 73/230 (31.7%) were scanned in external centres. The study cohort was split into training set (158 patients, 165 HCCs) and held-out test set (72 patients, 77 HCCs), stratified by random partitioning, which was repeated 100 times, and by a temporal partitioning to simulate the sequential development and clinical use of the radiomics model. A machine learning model for the prediction of MVI was developed with least absolute shrinkage and selection operator (LASSO). The concordance index (C-index) was used to assess the value to predict the recurrence-free (RFS) and overall survivals (OS).

**Results:** In the 100-repetition random partitioning cohorts, the radiomics model demonstrated a mean AUC of 0.54 (range 0.44–0.68) for the prediction of MVI, mean C-index of 0.59 (range 0.44–0.73) for RFS, and 0.65 (range 0.46–0.86) for OS in the held-out test set. In the temporal partitioning cohort, the radiomics model yielded an AUC of 0.50 for the prediction of MVI, a C-index of 0.61 for RFS and 0.61 for OS, in the held-out test set.

**Conclusion:** The radiomics models had a poor performance for the prediction of MVI with a large variability in the model performance depending on the random partitioning. Radiomics models demonstrated good performance in the prediction of patient outcomes.

### SS 7.7

### Predicting Pd-l1 expression and survival outcome in HCC patients receiving Sorafenib treatment after hepatectomy based on contrast-enhanced CT texture signature

C. Yang; Chengdu/CN

**Purpose:** Overexpression of programmed cell death protein ligand-1 (PD-L1) could cause tumor resistance to sorafenib in patients with HCC. Our study aimed to predict the expression of PD-L1 and prognostic outcomes of HCC patients who received sorafenib treatment after surgical resection (SR) using contrast-enhanced computer tomography (CECT) texture signature.

**Material and Methods:** This retrospective study included 88 HCC patients who received sorafenib treatment after SR. Immunohistochemistry was used to assess the expression of PD-L1. Texture features were extracted based on CECT. The related significant features of PD-L1 expression were determined by the logistic regression analysis. The diagnostic performance of texture features was assessed by the area under the curve (AUC). Cox proportional-hazards model and Kaplan–Meier analysis were employed to select predictive features associated with recurrence-free survival (RFS) and overall survival (OS).

**Results:** All the HCC patients were divided into a PD-L1 positive (PD-L1+) group (n = 42) and a PD-L1 negative (PD-L1-) group (n = 46). Four wavelet features were proved associated with the PD-L1 expression. The wavelet_glszm_wavelet-LHL-ZoneEntropy feature achieved the best discriminative performance of the area under the curve (AUC) as 0.708 [95% confidence interval (CI)= 0.595–0.821]. PD-L1+ was significantly related to worse RFS and OS (both P<0.05). The wavelet_glszm_wavelet-HLH-LowGrayLevelZoneEmphasis feature was associated with the risk of HCC recurrence (hazard ratio [HR], 0.43; 95% CI, 0.27–0.68; P<0.001) and overall patient mortality (HR, 0.34; 95% CI, 0.15–0.79; P=0.012).

**Conclusion:** The CECT texture signature may be an efficient technique for discriminating PD-L1 expression and predicting the survival of HCC patients receiving sorafenib treatment after SR.

### SS 7.8

### A radiomics approach to noninvasively predict programmed cell death protein-1 expression and prognosis in patients with HCC treated with sorafenib after surgery

F. Che, S. Bin; Chengdu/CN

**Purpose:** To investigate the impact of preoperative contrast-enhanced CT-based radiomics model on programmed cell death protein-1 (PD-1) prediction in HCC patients.

**Material and Methods:** The study included 105 HCC patients (training cohort: 72; validation cohort: 33) who underwent preoperative contrast-enhanced CT and received systemic sorafenib treatment after surgery. Volumes of interest included were manually delineated in the portal venous CT images. The variance analysis, spearman correlation, and lasso regression analysis were used to select features to build a radiomics score using logistic regression analysis. Univariate and multivariate analyses were performed to determine factors associated with PD-1 expression. The radiomics model was established using multivariable logistic regression analysis. The Kaplan–Meier survival analysis was used to assess overall survival (OS) in the PD-1-positive and PD-1-negative patients.

**Results:** Seventeen radiomics features were finally selected to construct the radiomics score. In multivariate analysis, serum creatinine and peritumoral enhancement were significant independent factors for PD-1 prediction. The radiomics model integrated radiomics signature with serum creatinine and peritumoral enhancement showed good discriminative performance (AUC of 0.897 and 0.794 in the training and validation cohort). OS was significantly different between the radiomics-predicted PD-1-positive and PD-1-negative groups (OS: 29.66 months, CI: 16.03–44.40 vs. 31.04 months, CI: 17.10–44.07, P<0.001). Radiomics-predicted PD-1 was an independent predictor of OS of patients treated with sorafenib after surgery (hazard ratio [HR]: 1.61 [1.23–2.1], P<0.001).

**Conclusion:** The proposed model based on radiomic signature helps to evaluate PD-1 status of HCC patients and may be used for evaluating patients most likely to benefit from sorafenib as a potentially combination therapy regimen with immune checkpoint therapies.

### SS 7.9

### Alpha-fetoprotein response patterns after Y-90 radioembolization for intermediate-to-advanced HCC predict disease progression and survival

J.T. Wen, J.R. Tse; *Stanford, CA/US*

**Purpose:** To describe alpha-fetoprotein (AFP) response patterns in patients with AFP-producing Barcelona clinic liver cancer stage B or C HCC after Y-90 radioembolization and their correlation with overall survival (OS), distant progression-free survival (DPFS), and progression-free survival (PFS).

**Material and Methods:** This single-center, retrospective study evaluated 106 patients from 2011 to 2022 with AFP-producing HCC (≥20 ng/mL) without extrahepatic metastases who underwent Y-90 radioembolization. Pre-treatment and post-treatment imaging were evaluated, latter with mRECIST. AFP response was categorized into 3 patterns: 1) no response (<20% AFP decrease or increase), 2) transient response (decrease by ≥20%, followed by increase at any time point thereafter), and 3) sustained response (persistent AFP decrease by ≥20%). OS, DPFS, and PFS were estimated using Kaplan–Meier curve analyses. Multivariate analyses for OS and PFS were assessed with Cox proportional hazards regression.

**Results:** Twenty-five (24%) patients had sustained response, 35 (33%) had transient response, and 46 (42%) had no response. Median OS of sustained, transient, and no response were 9.0, 7.3, and 6.5 months, respectively (p=0.019). Median DPFS of sustained, transient, and no response were 7.7 months, 5.5 months and 3.6 months, respectively (p<0.001). Median PFS of sustained, transient, and no response were 7.7, 2.9, and 2.7 months, respectively (p<0.001). At multivariate analyses, ECOG Performance Scale, AFP response, and Child–Pugh class were independent prognostic indices of OS. Baseline AFP and AFP response were independent prognostic indices of PFS.

**Conclusion:** More than half of patients have an initial AFP response relapse at follow-up and have only slightly better outcomes compared to those with no response.

### SS 7.10

### Diagnostic and prognostic models for abdominal radiology: how many are evaluated externally?

M. Hameed, J. Yeung, D. Boone, S. Mallett, S. Halligan; *London/GB*

**Purpose:** Prognostic and diagnostic models must work in their intended clinical setting, proven via “external evaluation”, preferably by authors uninvolved with model development. By systematic review, we determined the proportion of abdominal imaging models published in high-impact radiological journals that are evaluated subsequently.

**Material and Methods:** We hand-searched three radiological journals for multivariable diagnostic/prognostic models related to abdominal imaging, 2013–2015 inclusive. We assessed completeness of data presentation to allow subsequent external evaluation. We then searched literature to August 2022 to identify external evaluations of these index models.

**Results:** We identified 36 index studies (23 prognostic; 13 diagnostic) with hepatopancreaticobiliary, the commonest theme (n=16). Eight presented an evaluation, only one of which was external. No model was updated. Six studies presented a model equation. 27% (4/15) of studies developing Cox models presented a risk table and 13% presented the baseline hazard. Just one of 21 studies developing non-Cox models presented the intercept. 12 studies presented a Kaplan–Meier curve of the final model. The 36 studies attracted 1494 citations (median 32, 202 self-citations). There were just five external evaluations of an index model, four of which were by researchers uninvolved with model development, and from a different institution.

**Conclusion:** Very few abdominal radiology prognostic or diagnostic models are evaluated externally, suggesting wasted research effort and resources. Authors’ published models should present data sufficient to allow external evaluation by others. To maximise clinical utility, researchers should concentrate on model evaluation and updating instead of continual redevelopment.

**11:00 - 12:30** **Room 3**

*Scientific Session SS 8*

### Pancreatic adenocarcinoma: response assessment and prediction of complications

### SS 8.1

### Variation in the diameter of the main pancreatic duct during neoadjuvant treatment of resected cephalic pancreatic adenocarcinoma: correlation with histological response

A. Gence-Breney, R. Sartoris, M. Dioguardi Burgio, L. de Mestier, S. Dokmak, A. Sauvanet, V. Vilgrain, M. Ronot; *Clichy/FR*

**Purpose:** To assess the value of the variation in the main pancreatic duct (MPD) diameter in predicting the histopathological response to neoadjuvant treatment in patients with resected pancreatic ductal adenocarcinoma (PDAC).

**Material and Methods:** CT at baseline and after neoadjuvant treatment of patients treated by pancreaticoduodenectomy for PDAC between 2016 and 2021 were analyzed. The variation of the maximum MPD diameter was evaluated. Histopathological response was assessed by the CAP score. Radiological response was assessed per RECIST 1.1 considering locoregional vascular invasions. Radio-pathological correlations were performed.

**Results:** 156 patients were included (76 women; mean 63±9 years). Twenty-one tumors were upfront resectable (13.5%), 121 borderline resectable (77.5%), and 14 locally advanced (9%). All patients received chemotherapy (FOLFIRINOX 89%), and 128 radiochemotherapy (82%). On pathology, most tumors were CAP-2 (69.2%), 10 were CAP-0 (6.4%), 19 were CAP-1 (12.2%), and 19 were CAP-3 (12.2%). After neoadjuvant treatment, the MPD diameter, tumor size, and attenuation decreased (p=0.004, p<0.001, and p<0.001). Vascular invasions were not significantly modified, except for venous stenosis, which decreased (p<0.001). On imaging, 39.7% and 59% of patients showed objective response or stability. There was no correlation between the radiological and histopathological response (p=0.065) but an MPD diameter decrease was associated with histopathological response (p=0.001). Combining the radiological assessment and a 25% decrease in the MPD diameter improved the histopathological response prediction (correct classification 122/156 (78.2%) vs. 97/156 (62.2%) patients).

**Conclusion:** The variation in MPD diameter after neoadjuvant treatment seems associated with the histopathological response in patients with resected PDAC. This additional feature may help refine the radiological assessment of the tumor response.

### SS 8.2

### CT evaluation of ectasia of the gastrocolic trunk, jejunal and colic veins as a secondary sign of venous infiltration after neoadjuvant therapy in patients with pancreatic adenocarcinoma

M. Borzi, G.A. Zamboni, E. Boffa, M. Bariani, G. Malleo, G. Mansueto*; Verona/IT*

**Purpose:** To analyze the correlation between ectasia of the gastrocolic trunk, jejunal, and colic veins and infiltration of the portomesenteric veins after neoadjuvant therapy (NAT) in patients with pancreatic adenocarcinoma.

**Material and Methods:** This retrospective study included patients who underwent pancreaticoduodenectomy (PD) after NAT, and had a preoperative contrast-enhanced CT available for review (PAD-R registry, n1101CESC). Patients were divided into one group that had undergone venous resection, and a second group that had not. For patients who underwent venous resection, pathology specimens were reviewed for invasion and classified as positive or negative neoplastic venous infiltration. Two readers in consensus reviewed the last preoperative CT and analyzed: tumor size and location, contact with peripancreatic vessels, resectability based on NCCN guidelines, and presence of ectasia of the gastrocolic trunk, jejunal, and colic veins.

**Results:** We included 135 patients who underwent NAT and PD between January 2013 and December 2021. 65 patients (48%) underwent venous resection during PD: 23/65 patients (35%) had venous ectasia on CT. In 20/23 (87%), neoplastic venous infiltration was confirmed at pathology. 70 patients (52%) did not undergo venous resection, 52/70 (74%) without venous ectasia on CT. A statistically significant correlation was observed between venous resection and venous ectasia on CT (p=0.0329) and between histological venous infiltration and venous ectasia on CT (p=0.0005).

**Conclusion:** The presence of ectasia of the gastrocolic trunk, jejunal and colic veins appears to be correlated with venous neoplastic infiltration at histology. This could help overcome the difficulty of differentiating between fibrosis post-NAT and neoplastic vascular infiltration.

### SS 8.3

### CT features of chemotherapy-associated liver injury to predict postoperative complications in patients undergoing pancreaticoduodenectomy for pancreatic ductal adenocarcinoma

F. Tessier, R. Sartoris, T. Codjia, M. Dioguardi Burgio, L. de Mestier, S. Dokmak, A. Sauvanet, V. Vilgrain, M. Ronot*; Clichy/FR*

**Purpose:** To assess the CT changes associated with chemotherapy-associated liver injury (CALI) and their influence on severe postoperative complications in patients undergoing pancreaticoduodenectomy (PD) after neoadjuvant oxaliplatin-based chemotherapy for pancreatic ductal adenocarcinoma (PDAC).

**Material and Methods:** Patients undergoing PD after oxaliplatin-based chemotherapy for PDAC (2017–2020) were included. Baseline and preoperative CT were reviewed to extract qualitative and quantitative features. Severe complications were defined as Clavien–Dindo ≥3. Features associated with CALI and severe complications were identified by paired univariate analysis.

**Results:** 122 patients were analyzed (64 men, 52%; median 65 yrs [IQR, 57–69]). Fifteen tumors were upfront resectable (12%), 97 were borderline resectable (80%) and ten were locally advanced (8%). Most patients received FOFLIRINOX (88%, median of 6 cycles) and 79% received radiotherapy. After chemotherapy, the spleen volume, hepatic parenchymal heterogeneity, and portosystemic shunts increased (p<0.001). Liver surface nodularity did not significantly increase (p=0.065). A lower precontrast hepatic attenuation and heterogeneity of the hepatic parenchyma on the preoperative CT were associated with severe complications (p<0.001). Precontrast liver attenuation inversely correlated with the comprehensive complication index (rho −0.329, p<0.001). More granular analysis showed that CT features were mostly associated with ascites and sepsis, but not with clinically significant pancreatic fistula.

**Conclusion:** Neoadjuvant oxaliplatin-based chemotherapy for PDAC leads to CT changes, including splenic volume increase, heterogeneous hepatic enhancement, and portosystemic shunts. In addition to clinical and surgical characteristics, lower precontrast hepatic parenchyma attenuation and heterogeneous enhancement were associated with severe liver-related complications after PD.

### SS 8.4

### CT evaluation of liver steatosis after neoadjuvant chemotherapy as a predictive factor for surgical complications in patients with pancreatic adenocarcinoma

E. Boffa[1], A. Spezia[1], G.A. Zamboni[1], G. Mansueto[2]*;*
*[1]Verona/IT, [2]Verona/IT*

**Purpose:** To evaluate the liver density changes on CT after neoadjuvant treatment (NAT) for pancreatic adenocarcinoma to quantitatively assess drug-induced hepatic damage and evaluate the role of moderate/severe steatosis as a predictive factor for post-operative complications.

**Material and Methods:** Informed consent for the utilization of clinical and radiologic data was provided by all patients (PAD-R registry, n1101CESC). IRB approval was not required for this retrospective study. Two readers reviewed the pre- and post-chemotherapy CT scans performed between April 2019 and February 2022 on 76 patients with pancreatic adenocarcinoma who received NAT and went to surgery. Liver steatosis was evaluated by drawing multiple ROIs on liver and spleen parenchyma (6 on liver, 4 on spleen) on the venous phase, obtaining the mean difference in density between liver and spleen. The population was divided into 2 groups based on steatosis degree after NAT (group 1: <30%; group 2: >30%), then post-surgical complications were compared across groups applying the chi-squared test.

**Results:** Twenty-eight patients (37%) developed or worsened their steatosis degree after neoadjuvant chemotherapy. 42 patients (55%) developed post-surgical complications (POPF, liver failure, bile leak, PPAP). Moderate/severe steatosis had a positive trend of correlation with post-surgical PPAP, although not significant, and in the non-PPAP patients was significantly correlated with post-surgical complications frequency (Clavien–Dindo index after 90 days >3).

**Conclusion:** Moderate/severe liver steatosis after neoadjuvant treatment demonstrates a correlation with post-surgical complication frequency, thus reflecting an increased surgical risk in patients with chemotherapy-induced liver damage.

### SS 8.5

### MRI and CT findings after irreversible electroporation for locally advanced pancreatic cancer

M. Vionnet, B. Belardy, J. Damion, J. Goujon, S. Velasco, A. Coulibaly, G. Herpe, D. Tougeron, J.-P. Tasu*; Poitiers/FR*

**Purpose:** Locally advanced pancreatic cancer (LAPC) accounts for 30% of patients with pancreatic adenocarcinoma. In LAPC, irreversible electroporation (IRE) has been tested to improve survival and quality of life. However, imaging findings post-IRE remains described in only 2 previous reports. The aim of this study was, therefore, to describe pancreas post-IRE imaging findings.

**Material and Methods:** This study is an ancillary study from a prospective single-center, open-label, non-randomized study called IRECAP (clinicaltrials.gov identifier: NCT03105921). All participants provided written informed consent. Patients underwent enhanced CT and MRI before IRE and within six days (D6), one month (D30), and three months (D90) after IRE. On CT and MRI, 4 patterns were evaluated, normal pancreas parenchyma, fat inflammation, tumor and necrosis. Only descriptive statistic data were performed.

**Results:** 15 patients were included (mean age 61 years, range 37–77). After IRE, ablation zone was bigger in size than the tumor targeted, without clearly demarcated margins due to peripheral inflammation. Transient necrosis was observed in all patients. A tissular mass can be detected at one month after IRE inside the treated area. The nature of this mass, tumoral or fibrotic, remains unknown but pathological analysis is on-going and results will be presented for the congress. Vascular involvement seems unmodified after IRE.

**Conclusion:** Evaluation of IRE for LPAC treatment by CT and MRI remains difficult considering complexity of post-therapeutic changes including inflammation, fibrosis, residual tumor and normal parenchyma.

### SS 8.6

### Utility of contrast-enhanced US in the diagnosis of acute venous pancreas graft thrombosis

C. Perez-Serrano, J. Soler, C. Bassaganyas, A. Darnell, G. Rafart Martinez, J. Ferrer-Fabrega, A. Garcia-Criado*; Barcelona/ES*

**Purpose:** To determine the value of contrast-enhanced US (CEUS) in the diagnosis of acute venous pancreas graft thrombosis when colour Doppler US (CDUS) identifies neither flow nor thrombus.

**Material and Methods:** We performed a retrospective review of pancreas transplant patients in our centre from January 2009 to January 2021. We considered acute venous thromboses those occurring during the first 30 days after transplantation. Our protocol includes at least one CDUS at 24–48h post-transplant and another at 7 days. CEUS was performed when CDUS detected neither flow nor thrombus in the splenic or mesenteric veins of the pancreatic graft.

**Results:** During this period, 247 pancreas transplants were performed. The CDUS detected 35 venous thromboses. CEUS was performed in 28 other cases where CDUS did not identify either flow or thrombus. In 15 of these patients, CEUS demonstrated that the splenic and mesenteric veins were permeable, but with low flow. In 11 cases, thrombosis was confirmed by angio-CT or arteriography. In one case, angio-CT showed misdiagnosis of thrombosis due to filiform vessels. The remaining case corresponded to peripheral thrombosis and no further tests were performed. There were only two cases of acute venous thrombosis in which the pancreas could not be assessed ultrasonographically and CT had to be performed.

**Conclusion:** Contrast-enhanced US (CEUS) allows the differentiation between venous low flow and thrombosis when no Doppler signal is found in the pancreas graft veins.

### SS 8.7

### Early intravoxel incoherent motion diffusion-weighted imaging for the prediction of post-pancreatectomy acute pancreatitis

L. Fortuna, G.A. Zamboni, L. Costa, B. Maris, E. Bannone, G. Marchegiani, G. Mansueto*; Verona/IT*

**Purpose:** This study aims to evaluate early diffusion-weighted (DW)-MRI radiological findings and texture analysis parameters that will predict the development of post-pancreatectomy acute pancreatitis (PPAP) in patients undergoing pancreaticoduodenectomy.

**Material and Methods:** 65 patients underwent MRI on the third postoperative day after pancreaticoduodenectomy. Scan protocol included standard sequences, post-gadolinium acquisitions, and intravoxel incoherent motion diffusion-weighted imaging (IVIM-DWI). IVIM DICOM images were analyzed with in-house software that produced F, D, and D* maps and allowed to calculate texture parameters of three different ROIs: the stump, the tail, and the entire pancreatic remnant. By retrospectively applying the 2021 ISGPS definition of PPAP, patients were divided into a group with and a group without POH/PPAP. Texture parameters and radiological findings were compared between the two groups (Kruskal–Wallis and ANOVA tests).

**Results:** 20 patients developed post-operative hyperamylasemia (POH) and 6 of these PPAP grade B or C. Significant differences in texture parameters were identified between the POH/PPAP and the non-POH/PPAP groups for mean ADC ((1.33±0.22 vs 1.56±0.28)×10-3mm$^2$/s; p=0.006) and D value (0.11±0.14 vs 0.22±0.15; p=0.03), F (4.5±0.10 vs 4.1±0.08 (SE); p=0.004) and D* (2.7±0.73 vs 1.3±0.38 (SE); p=0.01) entropy of ROIs including the pancreatic stump. Similar results were found evaluating ROIs of the tail and entire pancreatic remnant. No macroscopic features consistent with PPAP were identified.

**Conclusion:** Early postoperative MRI texture analysis of IVIM-derived parameters might predict who will develop PPAP after pancreaticoduodenectomy.

### SS 8.8

### The preoperative-fistula risk score: CT-based radiomics to preoperatively predict a postoperative pancreatic fistula in patients undergoing a pancreatoduodenectomy

E. Ingwersen[1], I. Verpalen[1], J. Bereska[1], B. Janssen[1], J. Stoker[1], A. Balduzzi[2], G. Marchegiani[2], R. De Robertis[2], Y. Nio[1], F. Struik[3], F. Daams[1]*; [1]Amsterdam/NL, [2]Verona/IT, [3]Lelystad/NL*

**Purpose:** Predicting postoperative pancreatic fistula (POPF) accurately can assist surgeons in making more informed treatment decisions. However, there is a lack of a preoperative, publicly available prediction models. Radiomic features, which provide new parameters and enable preoperative prediction, may be able to address this gap.

**Material and Methods:** Radiomic features were derived from preoperative CT scans from adult patients with an indication for a pancreatoduodenectomy at the Amsterdam University Medical Center to develop the preoperative-fistula risk score (pre-FRS). These radiomics features were analyzed with four machine learning classifiers. The model was then validated in an independent dataset from Verona University Hospital. The predictive performances of the models were evaluated using a range of metrics.

**Results:** A total of 118 patients were included in the model design cohort, with 50 (42.8%) of the patients developing POPF. The external validation cohort comprised 57 patients, of whom 22 (38.6%) developed POPF. The AUROC of the prediction model was 0.87 (95% CI: 0.71–0.98) on the model design cohort and decreased to 0.80 (95% CI: 0.69–0.92) on the external validation cohort. The calibration slightly improved in the external validation cohort compared to the model design cohort.

**Conclusion:** The pre-FRS was successfully validated in an external cohort and found to have adequate performance for predicting POPF. This preoperative prediction of POPF has the potential to improve patient prognosis and could assist surgeons making tailored treatment decisions.

### SS 8.9

### Correlation between visceral fat volume, sarcopenia and post-operative hyperamylasemia in patients who underwent major pancreatectomy

M. Bariani, M. Brotto, M. Borzi, G.A. Zamboni, G. Mansueto*; Verona/IT*

**Purpose:** Persistent post-operative hyperamylasemia (POH) >48 h is one diagnostic criterion for post-pancreatectomy acute pancreatitis (PPAP) according to the International Study Group of Pancreatic Surgery (ISGPS). Our purpose was to analyze correlations between visceral obesity, sarcopenia, and POH, to evaluate if these parameters can predict PPAP development after major pancreatic resections.

**Material and Methods:** Informed consent was provided by all patients (PAD-R registry, n1101CESC). We selected patients who underwent major pancreatectomy at the Verona Pancreas Institute in 2022 and had a CT scan between post-operative days 3 and 15. We divided the patients into 2 groups according to amylase values: group 1= normal values or POH<48 h; group 2= POH>48 h. We used a commercially available software (Syngo.via, Siemens) to segment visceral fat, paraspinal, and psoas muscles at the umbilical level. We logged volume (cm3) and mean density values (HU) for each. Quantitative variables were described as mean and compared by Student's t test.

**Results:** We included 30 patients, 16 M and 14 F, mean age 56.7. Group 1 had visceral fat volume (VFV) of 15.4 cm$^3$, with mean density (MD) values of -82.6 HU; muscles had volume of 8.2 cm$^3$ and MD of 22.1 HU. Group 2 had VFV of 13.4, with MD of –83.0; muscles had volume of 7.4 cm$^3$ and MD of 21 HU. No statistically significant differences were found comparing the findings in the 2 groups.

**Conclusion:** In our small series, markers of visceral obesity and sarcopenia do not appear to be correlated to persistent POH, and therefore, to the risk of PPAP.

### SS 8.10

### Dynamic contrast-enhanced US in pancreatic transplantation: normal values and its potential utility in evaluating rejection

C. Bassaganyas Vancells, J. Soler-Perromat, A. Darnell, V. Sapena, A. Soler-Perromat, C. Perez Serrano, J. Ferrer-Fàbrega, P. Ventura-Aguiar, Á. Garcia-Criado; *Barcelona/ES*

**Purpose:** Dynamic contrast-enhanced US (dCEUS) is a novel non-invasive ultrasonographic technique that allows quantification of tissue perfusion. This study aims to determine dCEUS values in normofunctioning vs rejection pancreatic grafts.

**Material and Methods:** Prospective study including all pancreas transplantations in our centre between October2016 and January2020. These were evaluated at 1 week (dCEUS), 3 weeks (dCEUS+biopsy) and 12 months (dCEUS+biopsy) after surgery. In addition, a dCEUS+biopsy was performed in all grafts with dysfunction or for surveillance following rejection treatment (regardless of transplantation date). Patients with postoperative complications were excluded. VueBox® was used to evaluate time–intensity curves, providing 12 parameters/dCEUS. Evaluations were classified according to the biopsy (normal/rejection) and compared. Those without biopsy were classified according to clinical management. Using Youden's criteria, cut-off values were determined for all evaluated parameters.

**Results:** During this period, 132 dCEUS studies and 85 biopsies were performed on 56 patients. Three patients were excluded because of postoperative complications (5 studies). Time–intensity curves showed a high dispersion of the values of all evaluated parameters during the first 3 months, which may be related to postsurgical factors. After this period, significant differences were observed in parameters dependent on the area under the curve (median [IQR], a.u.), with lower enhancement in the presence of rejection (wash-in AUC: 1589[1704] rejection vs 3386[5568] normal, p=0.007). In patients with concomitant biopsy, a cut-off value of 601 for peak enhancement and 118 for wash-in rate were found (AUC 0.64[0.53–0.76, 95%CI], p=0.026 for both).

**Conclusion:** After the first 3 months, acute graft rejection is associated with lower graft perfusion. These results highlight that dCEUS may be a helpful non-invasive tool to screen patients requiring pancreas graft biopsy or aid in diagnosing pancreas graft rejection.

**14:30 - 16:00** **Room 1**

*Scientific Session SS 9*

### Liver fibrosis and portal hypertension

### SS 9.1

### Performance of multifrequency MR elastography for diagnosing progressive forms of non-alcoholic fatty liver disease

E. Gonzalez-Montpetit[1], P. Garteiser[1], M. Dioguardi-Burgio[1], G. Pagé[1], D. Valla[1], L. Castera[1], M. Ronot[2], V. Vilgrain[2], V. Paradis[2], B. Van Beers[2]; *[1]Paris/FR, [2]Clichy/FR*

**Purpose:** Single-frequency MR elastography (MRE) has diagnostic potential for assessing the severity of non-alcoholic fatty liver disease (NAFLD) and viral hepatitis (Huwart 2008, Imajo 2016). Moreover, multifrequency MRE may be useful for activity grading in viral hepatitis (Garteiser 2021). However, the value of multifrequency MRE in assessing NAFLD is unknown. Here we investigated the performance of multifrequency MRE for diagnosing three definitions of progressive NAFLD, namely NASH (Bedossa 2014), NAFLD with significant fibrosis (Kim 2022) and fibro-NASH (Noureddin 2022).

**Material and Methods:** This study was conducted in a prospective, single-center recruitment, included on the basis of type 2 diabetes and fatty liver. MRE was acquired at 40, 60 and 80 Hz. Stiffness at each frequency was extracted and a dispersion coefficient was calculated using 40 and 60 Hz only. MRE results were compared against histologic analyses of liver biopsies, classified according to NASH, NAFLD-related fibrosis (NAFLD and F >= 2) and fibro-NASH (NASH with NAS >= 4 and F >= 2) using ROC analysis.

**Results:** 168 patients were recruited (101 men, 60%, median 59 years, range 30–79), among which 54% had NASH (average NAS 4.8), 53% had NAFLD-related fibrosis (average NAS 4.4) and 43% had fibro-NASH (average NAS 5.1). The dispersion coefficient had an AUC of 0.61 (95% CI 0.53–0.69, p=0.02) for diagnosing NASH, 0.72 (0.64–0.79, p < 0.0001) for NAFLD with significant fibrosis, and 0.66 (0.58–0.74, p=0.0004) for fibro-NASH.

**Conclusion:** MRE-derived stiffness dispersion coefficient has potential in diagnosing progressive NAFLD forms that take fibrosis into account.

### SS 9.2

### Prospective study to assess the prevalence of significant hepatic fibrosis through the application of one single-shot liver elastography (acoustic radiation force impulse) in a population of patients undergoing abdominal US in a tertiary hospital

I. Herraiz, A. Fernández-Moscoso, F. Del Amo, P. Bellot, D. Ferrández, Á. Palazón; *Alicante/ES*

**Purpose:** Liver fibrosis, especially associated with MAFLD, is a relatively frequent complication in the general population (5%). The determination of hepatic stiffness (Kpa) by non-invasive methods such as elastography is a useful tool for non-invasive diagnosis and screening. The objective is to estimate the prevalence of hepatic fibrosis by means of IFRA in a population of patients undergoing abdominal US for any reason.

**Material and Methods:** 559 patients underwent ultrasonography (+elastography in those patients with altered liver echogenicity) by three different radiologists.

**Results:** 30 of the 559 (5.3%) patients had a measurement compatible with significant liver fibrosis (F3–F4) with a cut-off point of > 9.5 Kpa, with a mean age of 54 years, 50% were women. Twenty-five patients underwent conventional elastography (median of 7 measurements) with 16 patients (64%) confirmed to have significant fibrosis. The most frequent cause of fibrosis was metabolic fatty liver disease (72%) followed by alcohol consumption (6%).

**Conclusion:** The prevalence of hepatic fibrosis is approximately 5% in the population undergoing US of any cause. The most frequent cause is hepatic steatosis. The screening elastography may be a useful tool for liver fibrosis screening.

### SS 9.3

### Usefulness of 2D shear wave elastography for evaluation of hepatic fibrosis and treatment response in patients with autoimmune hepatitis

E. Soh, Y. Lee, Y. Kim*; Iksan/KR*

**Purpose:** To determine the usefulness of 2D shear wave elastography (2D SWE) in the assessment of liver stiffness (LS) and dispersion slope (DS) to evaluate hepatic fibrosis and the treatment response of patients with autoimmune hepatitis (AIH).

**Material and Methods:** Patients diagnosed with AIH and underwent 2D SWE between June 2014 and June 2021 were enrolled in this retrospective study. We classified the patients into four groups according to the histologic stage of fibrosis (F1–F4). The baseline characteristics, laboratory test results, histologic results, and 2D SWE results were analyzed. We investigated the diagnostic performance of LS measurements in hepatic fibrosis staging and compared variables before and after steroid treatment for AIH.

**Results:** A total of 69 patients were analyzed. The LS values differed according to the stage of liver fibrosis ($P < 0.001$). The area under the curve of the LS value was 0.903, 0.815, and 0.854 for F2, F3, and F4, respectively. The diagnostic performance of LS measurement was significantly greater than that of serum biomarkers, except for fibrosis index-4 for F4 ($P < 0.05$). There was a significant difference in both the LS value and DS in patients who received steroid therapy during the follow-up examination ($P = 0.012$ and 0.011, respectively).

**Conclusion:** 2D SWE is a useful method for the assessment of hepatic fibrosis in patients with AIH. For follow-up examinations, LS and DS can be used as reliable parameters to evaluate treatment response to AIH.

### SS 9.4

### Automated T1 mapping quantification of the liver with deep-learning-based 3D T1 vibe Dixon liver parenchyma segmentation and affine transformation with voxel-wise co-registration

L. Zbinden[1], D. Catucci[2], L.A. Hulbert[1], M. Brönnimann[1], L. Ebner[1], A. Christe[1], V. Obmann[1], R. Sznitman[1], A. Huber[1]*; [1]Bern/CH, [2]Herrenschwanden/CH*

**Purpose:** To evaluate an automated T1 mapping quantification of the liver with deep-learning-based 3D T1 vibe Dixon liver parenchyma segmentation and affine transformation with voxel-wise co-registration.

**Material and Methods:** A dataset of 170 liver MRI was used to train a convolutional neural network for liver parenchyma segmentation by excluding the liver vessels on non-contrast T1 vibe Dixon in-phase acquisitions. 21 prospectively acquired liver MRI of healthy volunteers unseen by the neural network with T1 vibe Dixon and shMOLLI T1 mapping sequences were assessed by measuring parenchymal T1 relaxation time based on a manual ROI of the whole liver by excluding the liver vessels. The previously unseen T1 vibe Dixon in-phase acquisitions were then automatically segmented with the AI model and registered to the T1-maps using affine transformations. Subsequently, the segmentation was eroded along parenchymal and vessel borders to exclude partial-volume effects. Finally, T1-relaxation times with standard deviation and ROI voxel size were determined using the AI segmentation mask and compared with the manual annotations as the ground truth.

**Results:** The automated AI-based parenchyma ROI calculation achieved a mean voxel intensity of 862+/−72ms, compared to the manually annotated ROI with 846+/−72ms. The Spearman correlation between the automated AI and the manual calculation was 0.94 ($p<0.001$). ROI size did not differ significantly between AI and manual segmentation (5285+/−1092 vs. 4744+/−1257, $p=0.11$).

**Conclusion:** Automated T1 mapping quantification of the liver with deep-learning-based 3D T1 vibe Dixon liver parenchyma segmentation and co-registration allows for accurate sequence-agnostic automated region of interest calculation.

### SS 9.8

### A CT-based virtual portal vein pressure gradient: a noninvasive computational model for the diagnosis of portal hypertension

S. Wan[1], L. Ren[2], Y. Wei[3], B. Song[4]*; [1]Cheng Du/CN, [2]Beijing/CN, [3]Chengdu/CN, [4]Cheng DU/CN*

**Purpose:** Portal hypertension (PH) is the main consequence of liver cirrhosis, the invasive portal vein pressure gradient (PVPG) measurement remains the most accurate technique for diagnosing PH. This study aimed to develop a virtual portal vein pressure gradient (vPVPG) model simulating the PVPG measurement and to see if it could predict PH noninvasively using CT images.

**Material and Methods:** Ninety-seven patients were included in this retrospective study from March 2019 to July 2022. The patients were divided into a PH group (n=65) and a non-PH group (n=32) based on their portal hypertension history. Patients with PH underwent transjugular intrahepatic portosystemic shunt (TIPS) procedure for the PVPG measurements; all participants in the two groups underwent CT angiography. The 3D model of portal venous system was reconstructed, and vPVPG was then computed with this model and the computational fluid dynamics (CFD) simulation.

**Results:** vPVPG was identified statistically significant for discriminating between the PH (2.4, interquartile ranges (IQR) 1.62–3.04) and non-PH groups (1.03, IQR 0.66–1.41, $p < 0.001$), with an odd ratio (OR) of 7.07 (95% confidence interval (CI): 3.12,16, $p < 0.001$). The receiver operating characteristic (ROC) analysis showed that vPVPG had a satisfactory capability for diagnosing PH, with an area under the curve (AUC) of 0.891 (95% CI 0.828–0.954), an accuracy of 0.825, a sensitivity of 0.813, a specificity of 0.831, and a cut-off value of 1.157.

**Conclusion:** We confirmed that the computational model could be used to noninvasively diagnose portal hypertension in cirrhosis and may be used as a substitute for invasive transjugular PVPG measurements.

### SS 9.9

### Concomitant left gastric vein embolization during tips placement for acute variceal bleeding has no effect on its recurrence: results of an observational retrospective multicenter study

P. Calame[1], E. Delabrousse[1], J. Gelfi[3], A. Rodes[4], F. Douane[1], M. Fatima-Zohra[5], A. Bouvier[6], O. Sutter[7], J. Goupil[8], P. Papadopoulos[9], J.-R. Risson[1], L. d'Alteroche[10], M. Greget[11], R. Loffroy[12]*; [1]Besancon/FR, [3]Grenoble/FR, [4]Lyon/FR, [5]Toulouse/FR, [6]Angers/FR, [7]Avicenne/FR, [8]Nîmes/FR, [9]Pessac/FR, [10]Tours/FR, [11]Strasbourg/FR, [12]Dijon/FR*

**Purpose:** The objective of this study was to evaluate whether concomitant left gastric vein embolization (LGVE) during TIPS for acute variceal hemorrhage could reduce the risk of bleeding recurrence.

**Material and Methods:** A national multicenter observational study was conducted in 14 centers between January 2019 and December 2020. All cirrhotic patients who underwent TIPS placement for acute variceal bleeding were included. A propensity score for the occurrence of LGVE was calculated and we used propensity score-matched survival analysis and propensity score-adjusted multivariate analysis to assess the independent predictors of recurrent bleeding at day-42 and one year.

**Results:** In total, 356 patients were included (mean age 57.3±10.8 years; 340 males). Median follow-up was 11.2 months [IQR=1.2; 13.3]. The main indication for TIPS was pre-emptive TIPS (162/356, 46%), rebleeding despite secondary prophylaxis (105/356, 29%), and salvage TIPS (89/356, 25%). Overall, 128 patients (36%) underwent LGVE during the TIPS procedure. At day-42 and one year, rebleeding-free survival did not differ significantly between patients who underwent LGVE and those who did not (6/128 (5%) vs. 15/228 (7%) at day 42, and 11/128 (5%) vs. 22/228 (7%) at one year, respectively). By propensity score-matched analysis, the rebleeding rate was not lower in patients with LGVE. Multivariate analysis identified persisting PPG >10 after TIPS placement as the only predictor of bleeding recurrence.

**Conclusion:** In this multicenter national real-life study, we did not observe any benefit of concomitant LGVE during TIPS placement for acute variceal bleeding on recurrence rate.

### *SS 9.10*

### A CT-based virtual model of radiomics nomogram may be helpful for identifying severe esophageal varices in patients with liver cirrhosis

S. Wan, X. Zhang, B. Song; *Cheng Du/CN*

**Purpose:** Severe esophageal varices (EV) is the leading cause of GI bleeding that is life threatening in cirrhotic patients; we aim to explore whether the multiparametric radiomics nomogram combining the liver radiomics and relevant indices of EV on CT can be used for predicting severe EV.

**Material and Methods:** 136 consecutive patients from January 2020 to August 2022 with liver cirrhosis were enrolled in this study, patients were divided into a non-conspicuous EV group (mild-to-moderate EV, n=30) and a conspicuous EV group (severe EV, n=106) according to endoscopic results. The radiomic scores (Rad-scores) were constructed from radiomics features of regions of interest (ROIs) in left liver (LL) and right liver (RL), respectively. The multiparametric nomogram was established by the better performance Rad-score and EV-relevant indices, the calibration, discrimination and clinical usefulness of developed nomogram were evaluated using calibration curves, decision curve analysis (DCA) and net reclassification index (NRI) analysis, respectively.

**Results:** The LL Rad-score was selected with relatively higher area under the curve (AUC; 0.88, training cohort; 0.865, validation cohort), and cross-sectional surface area (CSA) of EV was identified as the important predictor ($P<0.05$). The multiparametric nomogram containing LL Rad-score and CSA showed better predictive performance and good calibration in training (C-index, 0.953 [95%CI, 0.892 to 0.973]) and validation cohort (C-index, 0.938 [95%CI, 0.841 to 0.961]), resulting in an improved NRI (categorical NRI of 25.9% (P=0.0128), continuous NRI of 120% (P=0)). DCA demonstrated that the multiparametric radiomics nomogram was clinically useful.

**Conclusion:** Multiparametric radiomics nomogram on CT, which incorporates the liver radiomics signature and EV-relevant indices, is a useful tool for non-invasively predicting severe EV to complement endoscopy and may be helpful to prevent the subsequent GI bleeding.

**09:00 - 10:30** **Room 1**

*Scientific Session SS 10*

### Rectal cancer and colorectal liver metastases

### SS 10.1

### Diagnostic performance of dedicated high-resolution rectal CT for rectal cancer staging: comparative results with high-resolution rectal MRI

S. Kim, J. Suh, B. Hur, S. Jeon, J. Bae*; Seoul/KR*

**Purpose:** To compare the diagnostic performance of high-resolution dedicated rectal CT in preoperative rectal cancer staging to conventional CT, with high-resolution MRI results as gold standard.
**Material and Methods:** Fifty-one patients who underwent CT and MRI with rectum distension for preoperative rectal cancer staging were enrolled. High-resolution CT images were obtained with a quadrupled-matrix size and oblique multiplanar reconstruction. Two radiologists compared the diagnostic performance of T staging, extramural depth of invasion (EMD), ≤T2/≥T3, extramural venous invasion (EMVI), mesorectal LN metastasis between conventional CT and high-resolution CT, considering MRI results as gold standard. Results were compared using the Chi-square test, Fisher's exact test, linear weighted kappa, ROC analysis, and Pearson's correlation coefficients.
**Results:** Compared to conventional CT, high-resolution rectal CT showed higher accuracy in T staging (reviewer 1, 82.4% vs. 76.5% [P=0.463]; reviewer 2, 82.4% vs. 62.7% [P=0.027]) and better correlation to MRI (weighted kappa; reviewer 1, 0.89 vs. 0.83; reviewer 2, 0.82 vs. 0.64 [Ps<0.001]). In categorizing ≤T2/≥T3, high-resolution CT showed better correlation with MRI than conventional CT (weighted kappa; reviewer 1, 0.87 vs. 0.78; reviewer 2, 0.74 vs. 0.57). Reviewer 2 yielded better correlation to MRI in high-resolution CT than conventional CT in EMD (Pearson's coefficient; 0.97 vs 0.91) and EMVI (weighted kappa; 0.78 vs 0.47), while the difference was minimal in reviewer 1. Accuracy of mesorectal LN metastasis did not significantly differ between both CT modalities.
**Conclusion:** High-resolution rectal CT showed better performance in the T staging of rectal cancers than conventional CT, considering high-resolution MRI as gold standard.

### SS 10.2

### What are the barriers preventing template-style reporting for rectal MRI by GI radiologists?

C. Muthoo[1], P. Brown[2], H. Rossington[1], S. Alderson[1], P. Quirke[1], D. Tolan[1]*; [1]Leeds/GB, [2]York/GB*

**Purpose:** Complete rectal cancer MRI (RC-MRI) reports are essential for colorectal cancer multi-disciplinary teams (CRC-MDTs) treatment planning. While template-style reporting improves reporting standards, adoption is not widespread. Previous research has shown that dedicated quality improvement programmes can significantly increase template-style adoption rates but the barriers hindering radiologist adoption are poorly understood.
**Material and Methods:** Detailed recorded interviews were conducted with sixteen specialist radiologists involved in a bowel cancer improvement programme from thirteen English CRC-MDTs serving a population of 5.2 million. Interviews were performed by a single experienced radiologist interviewer following the same format with 20 minutes of questions. The responses were recorded by two researchers and collated to ensure no discrepancies were present. 20 interview questions were created using the Consolidated Framework for Implementation Research to assess barriers in 5 key domains: intervention, inner setting, outer setting, characteristics of individuals and process.
**Results:** 50% of interviewed radiologists reported they were unfamiliar with the evidence base in favour of template style reporting and 37.5% felt there was no significant advantage compared to free text. 43.75% had problems incorporating templates into their workflow. 68.75% reported initial negative attitudes towards the template, either personally or from colleagues.
**Conclusion:** Despite prior education and dissemination of evidence that template reporting improves the completeness and quality of primary staging rectal MRI reports, several key barriers to comprehensive adoption have been identified.

### SS 10.3

### Can T2W texture analysis of the primary tumour differentiate nodal status after neoadjuvant chemoradiation in rectal cancer?

D. Van der Reijd[1], R. Dijkhoff[1], S. Drago[2], J. van Griethuysen[1], D. Lambregts[1], F. Bakers[3], R. Beets-Tan[1], M. Maas[1]*; [1]Amsterdam/NL, [2]Monza/IT, [3]Maastricht/NL*

**Purpose:** The morphological assessment of lymph nodes after chemoradiation remains a challenge. The aim of this study is to investigate if T2-weighted (T2W) texture analyses of the primary tumour derived from baseline and restaging MRI, can differentiate ypN0 from ypN+ after neoadjuvant chemoradiation (CRT) in rectal cancer.
**Material and Methods:** 170 rectal cancer patients underwent baseline and restaging MRI after neoadjuvant CRT. A volume of interest was manually delineated around the primary tumour on pre-CRT-T2W-MRI and around the tumour remnant or fibrotic scar on post-CRT-T2W-MRI. 10 histogram texture features were extracted with the use of PyRadiomics and delta features were calculated by comparing pre- with post-CRT features. Histopathological evaluation after resection classified patients as ypN0 or ypN+. Patients without signs of recurrence after >24 months of follow-up in the Watch&Wait program were also classified as ypN0. Means were compared with independent t tests and a significance level of p≤0.05 was applied.
**Results:** 42/170 patients had ypN+. On pre-CRT-MRI, only skewness was significantly higher in ypN+ patients (1.03 vs 0.776, p=0.028). On post-MRI, 5/10 features were significantly higher in ypN+, including mean (5.29 vs 4.19, p=0.005), median (4.97 vs 3.86, p=0.005), minimum (–0.756 vs –1.31, p=0.014), 10th percentile (2.24 vs 1.26, p=0.001) and 90th percentile (8.70 vs 7.47, p=0.022). 7/20 delta features were significantly smaller in ypN+ compared to ypN0. Additionally, delta-skewness was positive in ypN0 and negative in ypN+ (0.217 vs –0.0969, p=0.035).
**Conclusion:** T2W texture analysis derived from post-CRT MRI seems to have more discriminative value for ypN-status compared to pre-CRT MRI. Texture features related to signal intensities had higher values in ypN+ patients.

### SS 10.4

### Locoregional nodal regrowth in rectal cancer patients following a watch-and-wait approach: a diagnostic dilemma?

B.M. Geubels, M. Maas, G.L. Beets, B.A. Grotenhuis*; Amsterdam/NL*

**Purpose:** To evaluate the diagnostic aspects and management of (suspected) locoregional nodal regrowth in rectal cancer patients following watch-and-wait (W&W) after neoadjuvant treatment or local excision.
**Material and Methods:** 35 rectal cancer patients with suspected nodal regrowth on MRI during W&W were retrospectively identified. 27/35 followed W&W for a complete response after (chemo)radiation; 8/35 patients underwent a local excision without neoadjuvant treatment. MRIs, diagnostic strategy and treatment outcome were evaluated.
**Results:** 16/35 patients had initial cN+ disease. Median time from initial treatment and suspected nodal regrowth on MRI following W&W was 9 (6–20) months. After this first detection, 20/35 patients directly proceeded to treatment; half of them also had endoscopic luminal abnormalities. In the remaining 15/35 patients, the MRI was repeated after 2–3 months. MRI revisions showed that in 8/15 patients, the nodal regrowth was previously visible as the largest or most suspicious lymph node (LN) at primary staging. Growth of the suspected LN on repeated MRI was the decisive factor to proceed to treatment. Eventually, 34/35 patients were treated: 9 patients received (re-)(chemo)radiation and 33 underwent TME-surgery. In 27/33 patients, the nodal regrowth was pathologically confirmed in the surgical TME-specimen. Of the 6/33 patients without nodal involvement, 5 patients had pathologically confirmed luminal regrowth and 1 patient underwent unnecessary TME-surgery immediately after the first detection of suspected LN on MRI.
**Conclusion:** MRI is an important tool in the detection and follow-up of nodal regrowth in rectal cancer patients following W&W. Repeating MRI helps to identify nodal regrowth and to guide treatment decision.

### SS 10.5

### Prediction of neoadjuvant CRT response in rectal cancer: importance of imaging-based body composition parameters

F. Hisoğlu, E. Aydın, M. Ustun, T. Kaya, S. Sahin, B. Ergin; *Izmir/TR*

**Purpose:** Radiotherapy and/or chemotherapy is applied before the operation in medium-risk and high-risk rectal cancers. In recent years, interest in the relationship between body composition and prognosis in cancer patients has increased. In this study, we aim to investigate the importance of visceral fat quantification, sarcopenia and sarcopenic obesity before the neoadjuvant chemoradiotherapy (CRT) treatment of rectal cancer patients and evaluate the treatment response using those parameters.
**Material and Methods:** 78 patients who were diagnosed with rectum cancer and received neoadjuvant CRT treatment between 2010 and 2022, with preoperative abdominopelvic CT and rectum MRI were included. Muscle-fat distribution, visceral fat area (VFA), subcutaneous fat area (SFA), skeletal muscle area (SMA) measurements on axial images through L3 vertebra and mesorectal fat volume (MFV) measurement were performed on CT scan. Rectal tumor volume was measured on MRI. Pathological treatment response was assessed with the Modified Ryan score. Mann–Whitney U test and Pearson $\chi^2$ test were used for statistical analysis. ROC curve was used in the diagnostic power evaluation and $p < 0.05$ was considered significant.
**Results:** There was no statistically significant difference between the group responders and non-responders, in terms of SFA, VFA, total fat area (TFA), tumor volume, SMA, sarcopenia, and sarcopenic obesity. On the other hand, MFV was significantly less in the non-responder group. Ryan score decreased as MFV increased (p=0.02). In addition, there was weak positive correlation between MFV and SFA (p<0.01), VFA (p<0.01) and TFA (p<0.01).
**Conclusion:** CT-based mesorectal fat volume measurements, which are non-invasive and easy to apply, are important parameters that can be used practically in the prediction of neoadjuvant treatment response in rectal cancer patients. Future studies are needed for validation of this parameter.

### SS 10.6

### Association between dynamic contrast-enhanced MRI parameters and prognostic factors in patients with primary rectal cancer

S. Kim, H. Kim, K. Nam; *Busan/KR*

**Purpose:** We aimed to evaluate the association between perfusion parameters derived from dynamic contrast-enhanced MRI (DCE-MRI) with prognostic factors in patients with primary rectal cancer.
**Material and Methods:** A total of 51 patients (31 men, 20 women; mean age, 69 years; range, 45–89 years) who had pathologically proven rectal adenocarcinoma and were treated via surgery were retrospectively enrolled. All the patients underwent preoperative DCE-MRI. Two blinded radiologists determined the tumor border after radiologic–pathologic correlation in each patient and drew regions of interest along the tumor border on consecutive slices bearing tumor to cover the whole tumor volume. The four perfusion parameters, including the volume transfer constant (Ktrans), were calculated under the extended Toft model. Tumor stage, lymph node stage, extramural venous invasion, Kirsten-ras mutation, carcinoembryonic antigen, circumferential resection margin status, tumor size and tumor differentiation were included as prognostic factors. The association was assessed via correlation or t test. In the case of significant prognostic factors, receiver operating characteristic (ROC) curve analyses were performed to estimate the diagnostic predictive values.
**Results:** Ktrans only showed a significant difference according to tumor differentiation, specifically, between the well-differentiated (n = 6) and moderately differentiated (n = 45) groups (0.127 ± 0.032, 0.084 ± 0.036, P = 0.036). The AUC was 0.838 (95% CI, 0.702–0.929), and the estimated accuracy, sensitivity, and specificity were 87, 90, and 60%, respectively. However, none of the other perfusion parameters showed significant differences in any of the prognostic factors.
**Conclusion:** Ktrans showed a significant difference according to tumor differentiation.

### SS 10.7

### A systematic review of prognostic models which incorporate imaging data that predict outcomes in rectal cancer

R. Mitchell-Hay, H. O'Brien, A. Murray, D. Mclernon; *Aberdeen/GB*

**Purpose:** To review the methodological quality and predictive performance of prognostic models that predict overall survival or response to chemoradiotherapy in rectal cancer patients.
**Material and Methods:** Medline, PubMed, The Cochrane Library and Web of Science were searched for papers concerning the development and validation of predictive and/or prognostic models for adult patients with rectal cancer that included both clinical and imaging data. Two authors independently screened titles, then subsequently abstracts and full texts. Any uncertainty was resolved by consensus. Data were extracted from the included papers using the CHARMS checklist with risk of bias assessed using the PROBAST tool.
**Results:** 5288 records were initially identified with 1766 duplicates removed prior to screening. 3523 records were screened with 95 full texts reviewed. 25 papers were included in the final review. 28% of models were externally validated on an independent data set. Following internal validation, AUC where it was reported ranged from 0.61 to 0.97. The overall risk of bias was recorded as high in 15 papers with unclear risk of bias in the remaining 10. High risk of bias was predominantly found in the analysis section of the PROBAST tool and primarily related to issues such as low event rate and univariate screening being used for predictor selection.
**Conclusion:** All models within rectal cancer that incorporate imaging data showed high or unclear risk of bias. Methodological improvements such as employing larger data sets and avoiding poor model building approaches could address these issues.

### SS 10.8

### Quantification of steatosis with dual-energy CT in patients with liver metastases from colorectal cancer: assessment of distribution

A. Borgheresi, A. Agostini, M. Marchegiani, E. Montecchiesi, L. Ottaviani, R. Rossi, M. Vivarelli, A. Giovagnoni; *Ancona/IT*

**Purpose:** To evaluate the role of dual-energy CT (DECT) in the assessment of the distribution of liver steatosis in patients with colorectal liver metastases.
**Material and Methods:** We retrospectively included patients (age> 18 years) with colorectal liver metastases, who underwent a DECT with a 3rd generation Dual-Source scanner (Somatom Force, Siemens, post-contrast DECT: 80–100/150Sn kV, modulated mA, iopamidol 370 mg I/mL, 1.3 ml/kg) between January 2018 and January 2019. The DECT venous dataset was processed with a 3-material decomposition algorithm (Liver VNC, Siemens). Two readers in consensus placed a 10 mm diameter circular region of interest (ROI) within 15 mm from liver metastases avoiding vascular structures, and an identical control ROI in a contralateral lesion-free liver segment. Statistical analysis was performed with non-parametric tests.
**Results:** 53 patients (39 M/14 F) were included, with a median age of 64 years, for a total of 102 nodules (median diameter 23 mm). The distribution of steatosis showed a median fat fraction of 4% in the perilesional site and 7% in the contralateral lesion-free parenchyma (Wilcoxon p = 0.046).
**Conclusion:** The quantification of liver steatosis from DECT datasets found subtle, but statistically significant differences between perilesional and lesion-free liver parenchyma.

### SS 10.9

### Multi-sequence MRI radiomics in colorectal liver metastases: which features are stable across readers?

D. Van der Reijd, E. Van Dijk, T. Buffart, B. Westerink, M. Monraats, E. Klompenhouwer, R. Beets-Tan, S. Benson, M. Maas; *Amsterdam/NL*

**Purpose:** Reproducibility is one the main challenges in radiomics. It is unclear how segmentation differences influence MR radiomics features derived from manual segmentation and whether features are stable between readers for different sequences. The aim of this study is to assess the stability of MR radiomics features across readers for multiple MRI sequences in colorectal liver metastases (CRLM).
**Material and Methods:** 30 CRLM (in 23 patients) were manually delineated by two sub-specialized abdominal radiologists on MRI before the start of chemotherapy on the portal-venous-phase (PVP), T2-weighted images (T2W) and b800 diffusion-weighted images (DWI). 107 radiomics features were extracted per sequence. An intraclass correlation coefficient (ICC) was calculated for each feature. Features with ICC>0.9 were classified as stable.
**Results:** 89% of PVP features showed ICC>0.9 with a median ICC of 0.985 (range 0.805–1.000). For DWI features, 79% showed ICC>0.9 with a median of 0.970 (range 0.272–1.000). The T2W features had a median ICC of 0.951 (range 0.513–0.999) and 68% showed ICC>0.9. When considering the lower bound of the 95% confidence intervals, 77%, 68% and 51% reached 0.9 for the PVP, DWI and T2W features, respectively. Noteworthy, the features skewness, elongation, flatness and sphericity showed ICC<0.9 in all three sequences.
**Conclusion:** MR radiomics features from b800-DWI, T2W and PVP showed very good stability for segmentations from two different readers. These preliminary results suggest that PVP features in colorectal liver metastases could be less susceptible for reader differences compared to DWI and T2W features. In the process of feature selection, intra-reader correlations can be a valuable step towards further establishing reproducibility.

### SS 10.10

### Deep learning-based detection algorithm for focal liver lesions in patients with colorectal adenocarcinoma: a comparison with radiologist

R. Sartoris[1], A. Paisant[2], C. Aubé[3], S. Malakzadeh[1], F. Matteini[1], A. Virzi[4], W. Boumalouka[4], V. Vilgrain[1], M. Ronot[1]; *[1]Clichy/FR, [2]Angers/FR, [3]Augers/FR, [4]Villepinte/FR*

**Purpose:** To assess the performance of a fully automated deep learning-based algorithm (DLA) for the detection of focal liver lesions on CT in patients with colorectal cancer.
**Material and Methods:** Retrospective bicentric study including consecutive patients with diagnosis of colorectal cancer who underwent abdominal contrast-enhanced CT. CT were reviewed by a senior radiologist and processed with the DLA; detected focal lesions were noted. Ground truth was established by two other senior radiologists in consensus considering all possible data (clinical information, MRI, follow-up, surgical report, pathology). Per-lesion, per-patient detection rates and the number of false positives were determined.
**Results:** 181 patients (103 men, (57%), mean±SD: 64±13 years) with 630 lesions (mean+SD 12.5±14.2mm) were analyzed. There were 282 liver metastases (LM) (44%, mean 19.1±17.5 mm) in 95 patients (52%). The radiologist identified 469/630 (74.4%) lesions, including 224/282 (79.4%) LM in 89 (93.7%) patients. The DLA had similar performance and detected 447/630 (70.9%, p=0.16) lesions, including 228/282 (80.8%, p=0.68) LM in 92 (96.8%) patients. There were 37 false-positive lesions in 32 patients with the radiologist reading and 39 false-positive lesions in 23 patients in the DLA reading. The detection rate of LM increased with size for both the radiologist and DLA (<4mm: 50% vs. 41.7% p=0.69; 5–9mm: 51.2% vs. 56.1% p=0.53; 10–14mm: 87.3% vs. 88.9% p=0.78; 15–19mm: 93% vs. 93%, p>0.99; ≥20mm: 98.8% vs 98.8%, p>0.99). The detection rate for the radiologist and DLA was not influenced by the location of lesions.
**Conclusion:** A deep learning-based detection algorithm offers promising detection performance, comparable to senior radiologist.

**09:00 - 10:30** **Room 3**

*Scientific Session SS 11*

### Oncology

### SS 11.1

### Validation of an automatic nnU-Net tool for neuroblastoma detection and segmentation in MR images

D. Veiga-Canuto, L. Cerda Alberich, A. Jiménez-Pastor, A. Gomis Maya, A. Alberich-Bayarri, L. Martí-Bonmatí; *Valencia/ES*

**Purpose:** To validate and assess the accuracy of a fully automatic nnU-Net CNN algorithm to identify and segment primary neuroblastoma tumors in MR images in a large children cohort.
**Material and Methods:** An international multicenter imaging repository of 300 patients from different European institutions within the PRIMAGE project with neuroblastic tumors was used to validate the performance of a previously trained tool based on the nnU-Net architecture, to identify and delineate primary neuroblastoma tumors. A dataset of 535 T2-weigthed sequences was included (486 sequences at diagnosis and 49 after finalization of first line of chemotherapy). For comparison, the segmentation masks were manually edited by an expert radiologist and the time for the manual editing was recorded. Different overlap and spatial metrics were calculated to compare both masks.
**Results:** The median Dice similarity coefficient (DSC) was extremely high (0.997). In 18 MR sequences (6%), the net was not able neither to identify nor segment the tumor. No differences were found regarding MR magnetic field, type of T2 sequence, or tumor location. No significant differences in the performance of the net were found in patients after treatment.
**Conclusion:** The automatic CNN was able to locate and segment the lesions on the T2-weighted images in 94% of cases. There was an excellent overlaid agreement between the automatic tool and the manually edited masks. The semi-automatic approach with minor manual editing of the deep learning segmentation increases the radiologist confidence in the solution.

### SS 11.2

### Comparison between MRI and pathology in the assessment of tumor regression grade in esophageal cancers

P. Chapellier, F. Fasquelle, C. Saglietti, R. Kinj, S. Mantziari, L. Haefliger, M. Jreige, N. Vietti Violi, C. Dromain; *Lausanne/CH*

**Purpose:** To assess the diagnostic performance of MRI tumor regression grade for tumor response evaluation of esophageal cancers.
**Material and Methods:** From 2018 to 2022, consecutive patients with newly diagnosed EC and planned neoadjuvant treatment underwent MR examinations for initial staging and restaging 4–6 weeks after adjuvant treatment. MrTRG classifications, based on fibrosis and tumor residue rate, equivalent to the Mandard (5-tier system) and Becker (4-tier system) classifications, were developed and assessed by 2 readers, first on T2W and DW MR images (unenhanced-MRI), and then with addition of enhanced T1W images (enhanced-MRI). The agreement between radiologist and pathologist was assessed with weighted Cohen's Kappa.
**Results:** 28 patients were included. Moderate agreement was found between mrTRG and pTRG when regression was classified according to Mandard (percent agreement (p)=88.4%, kappa (κ)=0.57) or Becker (p=84.5%, κ=0.52) on enhanced-MRI and p=87.5%, κ=0.54 and p=83.3%, κ=0.51, for Mandard and Becker, respectively, on unenhanced-MRI agreement between pTRG and mrTRG was moderate with a percent agreement (p)=88.4%, kappa (κ)=0.57 and p=84.5%, κ=0.52 for Mandard and Becker, respectively. Agreement was improved to substantial when grouping grades 1–2 for Mandard and 1a–1b for Becker with p=90.5%, κ=0.68 and p=89.3%, κ=0.65, respectively, for enhanced-MRI and p=85.7%, κ=0.64 and p=83.9%, κ=0.62, respectively, for unenhanced-MRI. Sensitivity and specificity of mrTRG in predicting pTRG were 88.3% and 80% for both Mandard (scores 1–2 versus 3–5) and Becker (scores 1a–1b versus 2–3).
**Conclusion:** The agreement between mrTRG and pTRG is substantial when grouping grade 1–2 suggesting mrTRG could be used as a surrogate of pTRG. Prognosis correlation should be assessed in further studies.

### SS 11.3

### Evaluation of sarcopenia as predicting factor of perioperative chemotherapy toxicity and response to treatment in patients with locally advanced gastric cancer

S. Nardacci, M. Zerunian, G. Arrivi, F. Mazzuca, D. Caruso, A. Laghi; *Rome/IT*

**Purpose:** In the management of patients with locally advanced gastric cancer, treatment is gastrectomy with perioperative chemotherapy (p-ChT). Unfortunately, not all patients tolerate chemotherapy and early interruption of therapy might affect the treatment strategy. Among non-invasive biomarker, sarcopenia has reached interest as possible predictor of drug toxicities and response to treatment. Thus, we aimed to test the role of sarcopenia in the prediction of p-ChT toxicity and response to treatment in gastric cancer patients.
**Material and Methods:** Patients with advanced gastric cancer who underwent contrast-enhanced CT prior to p-ChT and had images available were retrospectively enrolled. A total of 29 patients enrolled, 5 were excluded for CT unavailability. Skeletal muscle index (SMI) was assessed by manually segmented specific abdominal body region on portal-venous CT at L3 lumbar vertebra level with a dedicated software (ImageJ) and averaged for patients' height; SMI<52.4 and 38.5 $cm^2/m^2$ was considered as sarcopenic for male and female, respectively. Data on hematological, GI and neurological toxicity and response to treatment were recorded. Receiver operating characteristics (ROC) curve was obtained to assess sarcopenia status performance for p-Cht toxicity and response to treatment prediction; P<0.05 considered significant.
**Results:** 24 patients were enrolled (F:M=13:11), and 12 (50%) showed SMI values compatible with sarcopenia. Among p-Cht toxicities, sarcopenia showed a significant ability to predict both hematological and neurological toxicity (AUC=0.955 and 0.895, respectively, all P<0.001). GI toxicity and response therapy did not show significant results (P>0.05).
**Conclusion:** Evaluation of sarcopenia might represent an important non-invasive imaging biomarker to predict hematologic and neurologic toxicity in patients with gastric cancer before the beginning of chemotherapy to allow a personalized treatment management.

### SS 11.4

### The significance of conventional CT, CT perfusion and quantitative diffusion-weighted imaging in risk stratification of GI stromal tumors of the stomach

M. Mitrovic, A. Djuric - Stefanovic, K. Ebrahimi, J. Kovac, D. Saponjski, A. Jankovic, L. Lazic, S. Milosevic, K. Stošić, O. Skrobic, D. Masulovic; *Belgrade/RS*

**Purpose:** This study represents a prospective comparison of classical CT features, CT perfusion values, apparent diffusion coefficient and intravoxel incoherent motion-derived parameters. The purpose is to evaluate classical CT features, CT-perfusion and magnetic resonance diffusion-weighted imaging (MR-DWI)-related parameters in predicting the metastatic risk of gastric GIST.
**Material and Methods:** Sixty-two patients with histologically proven GIST who underwent CT perfusion and MR-DWI using multiple b values were prospectively included. Morphological CT characteristics and CT-perfusion parameters of tumor were comparatively analyzed in the high-risk (HR) and low-risk (LR) GIST groups. Apparent diffusion coefficient (ADC) and intravoxel-incoherent-motion (IVIM)-related parameters were also analyzed in 45 and 34 patients, respectively.
**Results:** Binary logistic regression analysis revealed that greater tumor diameter (p < 0.001), cystic structure (p < 0.001), irregular margins (p = 0.007), irregular shape (p < 0.001), disrupted mucosa (p < 0.001) and visible EFDV (p < 0.001), as well as less ADC value (p = 0.001) and shorter time-to-peak (p = 0.006), were significant predictors of HR GIST. Multivariate analysis extracted irregular shape (p = 0.006) and enlarged feeding or draining vessels (EFDV) (p = 0.017) as independent predictors of HR GIST (area under curve (AUC) of predicting model 0.869).
**Conclusion:** Morphological characteristics of the tumor detected by conventional CT examination still hold the greatest value in the preoperative risk stratification of gastric GIST. A significant statistical difference was shown regarding the functional CT perfusion parameters TTP and PS and especially the MR-DWI parameter ADC, which, together with classical CT features, can contribute to the more reliable prognosis of the biological behavior of GIST.

### SS 11.5

### Differential diagnosis of heterotopic pancreas and GI stromal tumor based on radiomics nomogram

C. Yang; *Chengdu/CN*

**Purpose:** To determine whether CT-based conventional parameters, relevant radiomics parameters, and combined multiparametric radiomics nomogram could be used to differentiate heterotopic pancreas from GI stromal tumor (GIST).
**Material and Methods:** A total of 84 consecutive patients with clinicopathologically confirmed heterotopic pancreas (n=29) and GIST (n=55) were retrospectively included in this study from May 2011 to May 2020. The 84 patients were randomly divided into a training set and a test set. The conventional imaging characteristics of the lesions were reviewed and a binary logistic regression model was used to construct a radiomics score (rad-score). The conventional imaging model, radiomics model, and radiomics nomogram were established, respectively, and these models were evaluated using ROC curve analyses and calibration curves.
**Results:** The diagnostic performance of rad-score built on the basis of radiomics parameters was also good, with AUCs of 0.81 (95%CI: 0.68 to 0.91) and 0.78 (95%CI: 0.60 to 0.91) in the training and validation sets, respectively. In addition, the multiparametric radiomics nomogram consisting of rad-score and significant conventional imaging parameters had optimal diagnostic performance in the training and validation sets with AUCs of 0.91 (95%CI: 0.79 to 0.97), and 0.83 (95%CI: 0.66 to 0.94); accuracy of 0.86 (95%CI: 0.74 to 0.94), and 0.73 (95%CI: 0.56 to 0.85).
**Conclusion:** The multiparametric radiomics nomogram based on the conventional imaging parameters and radiomics parameters can improve the diagnostic performance of identifying heterotopic pancreas and GIST.

### SS 11.6

### Small intestinal stromal tumors: associations between contrast-enhanced CT images and kit exon 9 gene mutation

C.-W. Yang, X.-J. Liu; *Chengdu/CN*

**Purpose:** Mutation screening for small intestinal stromal tumors (siGISTs) is crucial, and the c-kit gene (KIT) exon 9 mutation is more frequently found in the siGISTs. This study aimed to explore the associations between siGISTs with KIT exon 9 mutation and contrast-enhanced computed tomography (CECT) images.
**Material and Methods:** Pathologically proven siGISTs with definitive genotype testing results in our hospital were retrospectively included. Abdominal CECT images were analyzed. Conventional CT features and radiomics features were recorded and extracted to build the radiomics model, and the combined model (CT+radiomics). The diagnostic performances of siGISTs with KIT exon 9 mutation were evaluated.
**Results:** In total, 91 siGISTs (59 with KIT exon 11 mutation, 15 with KIT exon 11 mutation deletion involving codons 557–558, and 18 with KIT exon 9 mutation) were included. Significant CT features were found for siGISTs with KIT exon 9 mutation. The area under curves (AUCs) of the radiomics models for KIT exon 9 mutation were 0.8507 (95% CI: 0.7791–0.9223) in the training cohort, and 0.7824 (95% CI: 0.554–1) in the validation cohort. The AUCs of the combined models (CT + radiomics) for KIT exon 9 mutation were 0.887 (95% CI: 0.8191–0.9548) in the training cohort, and 0.912 (95% CI: 0.7915–1) in the validation cohort. The model (CT + radiomics) demonstrated the highest AUCs for prediction of KIT exon 9 mutation in siGISTs (P<0.05).
**Conclusion:** Our results demonstrated the associations between siGISTs with KIT exon 9 mutation sand contrast-enhanced CT images. CT radiogenomics exhibited good application potential in predicting the KIT exon 9 mutation of siGISTs.

### SS 11.7

### CT findings of GI mixed neuroendocrine/ non-neuroendocrine neoplasm: comparison with adenocarcinoma

H. Kim, S. Kim, H. Lee*; Seoul/KR*

**Purpose:** To investigate differential CT features of GI mixed neuroendocrine/ non-neuroendocrine neoplasm (MiNEN) compared with adenocarcinoma (ADC) as well as percentage of neuroendocrine component on histopathology.
**Material and Methods:** Forty patients with GI MiNEN (1 in esophagus, 22 in stomach, 1 in duodenum, and 15 in colorectum) and 38 patients with ADC were enrolled. The degree and homogeneity of enhancement for primary tumor, LN, or liver metastasis on CT were analyzed. Presence of intact overlying mucosa and EMVI were also assessed. For quantitative analysis, HU was measured for tumor, adjacent normal mucosa, metastatic LN, psoas muscle, liver metastasis, and normal hepatic parenchyma. Significant differential CT findings of MiNEN from ADC were determined using univariate analyses. Tumor-to-normal ratio was compared between MiNEN and ADC. For MiNEN, HU and ratio were correlated with % of neuroendocrine component using Pearson's correlation test.
**Results:** Compared with ADCs, GI MiNEN frequently showed intact overlying mucosa (1/38 [2.6%] vs 17/35 [48.6%], P<0.001) and exclusively accompanied with EMVI (9/35 [25.7%], P=0.001). GI MiNEN frequently showed heterogeneous enhancement on arterial (AP) and portal (PP) phases (PP: 13/38 [34.2%] vs 24/40 [60.0%], P=0.026; AP: 13/36 [36.1%] vs 14/23 [60.9%], P=0.055). GI MiNEN tended to have iso- or hyper-enhancing LN on AP with a marginal significance (10/19 [52.6%] vs 14/16 [87.5%], P=0.064). For GI MiNEN, HU of liver metastasis on PP was significantly correlated with the percentage of neuroendocrine component (correlation coefficient=0.796, P=0.032).
**Conclusion:** The presence of intact overlying mucosa with mucosal tenting and EMVI can be useful CT discriminators of GI MiNEN from ADC. Higher degree of enhancement in hepatic metastasis may represent larger component of neuroendocrine cells in GI MiNEN.

### SS 11.8

### Comparison of target lesion response by response evaluation criteria in solid tumors, modified response evaluation criteria in solid tumors, and Choi response criteria in patients treated with chemotherapy for GI neuroendocrine tumors

F. Cicalò[1], G. Tomelleri[1], M. Bariani[2], G.A. Zamboni[1], G. Mansueto[3]*; [1]Verona/IT, [2]Cerea/IT, [3]Verona/IT*

**Purpose:** Response criteria evaluate differently the response of target lesions. Our purpose is to compare response evaluation criteria in solid tumors (RECIST 1.1), modified RECIST (m-RECIST), and Choi criteria for the evaluation of response to treatment in patients with GI neuroendocrine tumors (GNET) treated with chemotherapy.
**Material and Methods:** IRB approval was not required for this retrospective study, which follows the Declaration of Helsinki. Two readers in consensus retrospectively analyzed 76 CT scans performed between 2018 and 2022 to assess response to chemotherapy in patients with GNETs. CTs were analyzed using RECIST 1.1, m-RECIST, and Choi criteria, assessing only target lesions. Chi-square test was used for analysis.
**Results:** Tumor response evaluation was concordant between RECIST, m-RECIST, and Choi criteria in 40 timepoints (52%) and in 52 timepoints (68%) between RECIST and m-RECIST. In 58 timepoints (76%), RECIST and Choi agreed on the tumor response evaluation and m-RECIST and Choi were concordant in 43 timepoints (56%). In 11 cases (14.5%), complete response (CR) was achieved according to m-RECIST but not according to RECIST 1.1 and Choi, which both agreed on a partial response (PR). No statistically significant differences were found between RECIST and CHOI (p=0.2150) and between RECIST and m-RECIST (0.2762).
**Conclusion:** Applying different response evaluation criteria may lead to different evaluations of response to chemotherapy in GNETs, although not statistically significant in our series. Further studies are required on the use of criteria other than RECIST 1.1, possibly with a correlation with PFS and OS to better understand the clinical significance.

### SS 11.9

### Comparison of 68Ga-DOTATATE positron emission tomography/CT and gadoxetic acid-enhanced MRI for the detection of liver metastases from well-differentiated neuroendocrine tumors

M. Drucker Iarovich, R. Hinzpeter, B. Moloney, K. Hueniken, P. Veit-Haibach, C. Ortega, U. Metser*; Toronto, ON/CA*

**Purpose:** To compare the detection of neuroendocrine tumor liver metastases (NLMs) on hepatobiliary-specific contrast-enhanced MRI (pMR) and 68Ga-DOTATATE positron emission tomography (PET)/CT (DT-PET).
**Material and Methods:** This retrospective study included patients with well-differentiated neuroendocrine tumors who underwent DT-PET and pMR. pMR and DT-PET were assessed independently by 2 readers who recorded number of NLMs per segment (1–4, &gt 5), SUVmax on DT-PET and signal characteristics on pMR. A second review was performed in consensus by a panel of two additional readers, settling discrepancies in number and location of metastases on both modalities.
**Results:** There were 30 patients with a mean age of 57 (±12) years. Concordance between DT-PET and pMR liver metastases count was demonstrated in 14/30 patients (47%). A greater number of NLM were identified on pMR in 12/30 patients (40%), and on DT-PET in 4/30 (13%) patients. Of the 12 patients with greater NLM count on pMR, there were 4 patients in whom multiple deposits were seen on pMR but only 0–1 lesions on DT-PET. Overall, pMR detected more metastases than DT-PET (p=0.01). When excluding the 4 outliers, agreement between the studies was nearly perfect (Kappa score: 0.945 [0.910, 0.980]). A relationship was found between increasing NLM lesion size and presence of metastases on DT-PET and presence of diffusion restriction on pMR and SUVmax on DT-PET.
**Conclusion:** pMR has a higher NLM detection rate than DT-PET. Excluding outliers in whom somatostatin receptor expression appeared limited, there is strong agreement between DT-PET and pMR for detection of NLMs in patients with well-differentiated NETs.

### SS 11.10

### Validation of the somatostatin receptor-reporting and data system 1.0 for the structured interpretation of somatostatin receptor-positron emission tomography/ CT and treatment planning in neuroendocrine tumor patients

F. Grawe*; Munich/DE*

**Purpose:** The recently proposed standardized reporting and data system (RADS) for somatostatin receptor (SSTR)-targeted positron emission tomography (PET)/CT (SSTR-RADS 1.0) showed promising first results in the assessment of diagnosis and treatment planning with peptide receptor radionuclide therapy (PRRT) in neuroendocrine tumors (NET). This study aimed to determine the intra- and interreader agreement of SSTR-RADS 1.0.
**Material and Methods:** SSTR-PET/CT scans of 100 patients were independently evaluated by 4 readers with different levels of expertise according to the SSTR-RADS 1.0 criteria at 2 time points within 6 weeks. For each scan, a maximum of five target lesions were freely chosen by each reader (not more than three lesions per organ) and stratified according to the SSTR-RADS 1.0 criteria. Overall scan score and binary decision on PRRT were assessed. Intra- and interreader agreement was determined using the intraclass correlation coefficient (ICC).
**Results:** Interreader agreement using SSTR-RADS 1.0 for identical target lesions (ICC ≥0.91) and overall scan score (ICC ≥0.93) was excellent. The decision to state "functional imaging fulfills requirements for PRRT and qualifies patient as potential candidate for PRRT" also demonstrated excellent agreement among all readers (ICC ≥0.86). Intrareader agreement was excellent even among different experience levels when comparing target lesion-based scores (ICC ≥0.98), overall scan score (ICC ≥0.93) and decision for PRRT (ICC ≥0.88).
**Conclusion:** SSTR-RADS 1.0 represents a highly reproducible and accurate system for stratifying SSTR-targeted PET/CT scans with high intra- and interreader agreement. The system is a promising approach to standardize the diagnosis and treatment planning in NET patients.

**11:00 - 12:30** **Room 1**

*Scientific Session SS 12*

### Liver and bile ducts

### SS 12.1

### Intraindividual comparison of contrast-enhanced US imaging of focal liver lesions acquired with 1.2 MI and 2.4 MI of sulfur hexafluoride

M. Mazzola[1], G. Pilato[1], R. Cannella[1], A. Taibbi[2], T. Bartolotta[1]; *[1]Palermo/IT, [2]Palermo/IT*

**Purpose:** To perform an intraindividual comparison of imaging features of focal liver lesions imaged with contrast-enhanced US (CEUS) with different doses of contrast agent.
**Material and Methods:** This study included adult patients with focal liver lesions imaged with CEUS acquired with two different doses (1.2mL and 2.4 mL) of sulphur hexafluoride (SonoVue). Two radiologists reviewed the CEUS examinations in two different sessions, evaluating the contrast-enhanced patterns in focal liver lesions. Lesions were scores as hypo-, iso-, or hypervascular in each post-contrast phase. Contrast-enhanced CT or MRI was used as reference standard for the final diagnosis. Differences in the enhancement patterns were compared using the Pearson chi-squared or Fisher's exact test and Mann–Whitney U test.
**Results:** A total of 61 patients (35 females and 26 males, mean age 53.2±16.3 years) with 61 focal liver lesions (26 malignant lesions of which 15 were diagnosed as HCC, 35 benign lesions of which 11 were haemangiomas) were included. Discordant enhancement patterns between different doses of contrast agent were observed in 8/61 (13.1%) lesions. In 5/61 (8.2%) lesions, CEUS acquired with 2.4 mL dose demonstrated more frequently hypervascularity in the arterial phase compared to 1.2 mL dose. There were no differences in diagnosis of malignancy (p=0.268), lesion size (p=0.414), BMI (p=0.240), and skin-to-lesion distance (p=0.160) between lesions with discordant and concordat enhancement patterns.
**Conclusion:** CEUS provides similar enhancement pattern in focal liver lesions with different doses of contrast agent, although hypervascularity in the arterial phase is more commonly observed with higher dose.

### SS 12.2

### Lean-body weight-based contrast injection protocol in liver CT: how low can e go?

A. Del Gaudio[1], D. De Santis[2], D. Pugliese[1], D. Valanzuolo[1], D. Caruso[2], A. Laghi[2]; *[1]Roma/IT, [2]Rome/IT*

**Purpose:** To determine the optimal lean body weight (LBW)-based contrast medium injection protocol for a uniform and optimal liver parenchymal enhancement.
**Material and Methods:** Forty-five patients who underwent a standardized multi-phase liver CT examination were randomly assigned to one of the following contrast media (CM) injection protocol groups: A, 700 mg iodine (I)/kg; B, 650 mgI/kg; and C, 600 mgI/kg. Magnitude of liver CT enhancement and signal-to-noise ratio (SNR) were calculated in portal venous phase. The total amount of iodine dose was also annotated and compared among protocols. Subjective image quality was evaluated with 5-point Likert scale. The Kruskal–Wallis and Mann–Whitney U tests were used to assess significant differences ($p < 0.05$).
**Results:** The total amount of iodine was significantly different among the three groups (A, 38.7 ± 1.8 g; B, 36.2 ± 1.3 g; C, 33.1 ± 1.4 g; $p < 0.042$). Group A obtained the highest liver enhancement (124.4 ± 18.3 HU) and SNR (8.4 ± 1.8), comparable to group B (120.3 ± 16.6 HU and 8.1 ± 1.6, respectively; $p > 0.068$). Group C obtained significantly lower liver enhancement (112.4 ± 17.9 HU) and SNR (7.5 ± 1.6) compared to group A ($p = 0.021$) and B ($p = 0.037$). No statistical differences were found among the three protocols in terms of subjective image quality (group A, 5 [IQR: 4–5]; group B, 5 [IQR: 4–5]; and group C, 4 [IQR: 3–5]; $p > 0.071$).
**Conclusion:** Group B allows significant iodine dose reduction while preserving liver enhancement, objective and subjective image quality.

### SS 12.3

### Evaluation of qualitative and quantitative imaging features of hepatic hemangiomas with and without pseudo-washout sign on gadoxetic acid-enhanced MRI: a descriptive and comparative study

S. Aslan, R.O. Onder, E. Sulun, M.N. Tasdemir; *Giresun/TR*

**Purpose:** (1) To evaluate the qualitative and quantitative features of hemangiomas on GA-enhanced MRI and (2) to compare imaging features of hemangiomas with and without pseudo-washout sign (PWS).
**Material and Methods:** We retrospectively included 93 hemangiomas who underwent GA-enhanced MRI. The presence of peripheral nodular or rapid enhancement in the arterial phase (AP), and PWSs in the transitional phase (TP) were evaluated. Signal-to-norm ratios (SINorm) of the hemangiomas, liver parenchyma, and portal vein (PV) as well as contrast-to-norm ratio (CNorm) were assessed. Additionally, hemangiomas with and without PWSs were defined as two separate subgroups, and qualitative and quantitative features were compared.
**Results:** Of 93 hemangiomas, 25 had rapid enhancement in the AP, and 49 had PWSs in the TP. In the TP, 52.6% of hemangiomas were hypointense. The mean SINorms of hemangiomas showed the highest value in the AP and then subsequently decreased ($p<0.05$). The mean CNorms showed positive values in the AP and gradually decreased to have negative values ($p<0.05$). In subgroup analyses, hemangiomas with PWSs were significantly smaller in size, and rapidly enhanced in the AP ($p<0.05$). The mean SINorms was lower in the TP, in hemangiomas with PWSs ($p=0.023$). The enhancement pattern of two subgroups was equal to that of the PV at all phases. The mean CNorms showed a significant difference in the AP between two subgroups ($p<0.001$).
**Conclusion:** When evaluating GA-enhanced MRI, radiologists should utilize quantitative measures in addition to qualitative assessment and should be aware that SI matching with PV can be a distinguishing finding in the diagnosis of hemangioma.

### SS 12.4

### Improving image quality and lesion conspicuity using deep learning-based superresolution reconstruction for hepatobiliary phase of gadoxetic acid-enhanced MRI

J. Kim, B. Kim, H. Lee; *Seoul/KR*

**Purpose:** This study aimed to compare 10-min and 20-min delayed hepatobiliary phase images (HBP) with deep learning-based super-resolution reconstruction (VIBESR) and with conventional reconstruction (VIBESD) in gadoxetic acid-enhanced MRI for adequacy of HBP and lesion conspicuity, and to determine whether 10min HBP with VIBESR could replace 20min-HBP with VIBESD.
**Material and Methods:** Eleven patients with 55 lesions were included. Perceived image adequacy, level of biliary enhancement, and signal intensity ratios (SIRs) of liver/inferior vena cava (IVC) were measured in eleven patients. Liver-to-lesion contrast (lesion CNR) and lesion conspicuity score (LCS) using a 4-point scale were evaluated in 55 lesions. Quantitative image analysis was compared using paired t test. LCS were compared using visual grading characteristics (VGC) and receiver operating characteristics (ROC)/area under the curve (AUC) analysis.
**Results:** SIRs of liver/IVC of VIBESD and those of VIBESR were significantly different at any time (10-min VIBESD, 1.44±0.36 vs. 10-min VIBESR, 1.66±0.56 [P = 0.014]; 20-min VIBESD, 1.81±0.60 vs. 20-min VIBESR, 2.00±0.46 [P=0.006]). SIR of liver/IVC of 10-min VIBESR and 20-min VIBESD was not significantly different (p=0.074). Lesion CNR of 10-min VIBESR was considerably higher than that of 20-min VIBESD (0.520±0.169 vs. 0.455±0.150, p = 0.0001). LCS of 10-min VIBESR and that of 20-min VIBESD was not significantly different (AUCVGC = 0.5), although LCS of 10-min VIBESD and that 20-min VIBESD was significantly different (AUCVGC = 0.592 [95% CI, 0.556–0.628]).
**Conclusion:** The VIBESR can potentially improve the image quality of gadoxetic acid-enhanced HBP and could shorten the time to obtain an adequate HBP.

### SS 12.5

### Imaging findings of portal cavernoma cholangiopathy on MRI: temporal evolution and correlation to biliary symptoms

E. Reizine[1], M. Ronot[2], A. Payance[2], K. Zekrini[2], S. Dokmak[2], P. Frederic[2], C. Bureau[3], P.-E. Rautou[2], A. Plessier[2], V. Vilgrain[2]; *[1]Créteil/FR, [2]Clichy/FR, [3]Toulouse/FR*

**Purpose:** To reassess the association between biliary abnormalities on MR cholangiography (MRC) and biliary symptoms in patients with portal cavernoma cholangiopathy (PCC) following non-cirrhotic extrahepatic portal vein thrombosis (NC-EHPVT).

**Material and Methods:** Monocentric retrospective study included 81 patients (47 men, 58%, median 46 yrs [38–55]) with a history of NC-EHPVT and a cavernous transformation of the portal vein. Biliary symptoms were collected at the time of the first available and follow-up MRCs. MRCs were reviewed, biliary abnormalities were graded as per Llop et al. and classified as per Chandra et al. Groups were compared with uni (Fisher and Mann–Whitney) and multivariate analysis (binary logistic regression model).

**Results:** Biliary abnormalities were present in 78/81 (98%) patients at inclusion. Most PCC were type III (67%) and grade II–III (74%). At inclusion, 14/81 (17%) patients with biliary abnormalities had biliary symptoms, including 13 with grade III PCC (93%). Among 46 patients with grade III PCC, a severe stricture of the common bile duct (CBD) was independently associated with symptoms (OR 4.08, 95%CI 1.27–13.18, p=0.02). Median follow-up was 8 years (IQR: 3.0–10.0), during which 62/81 patients (77%) underwent MRCs. Changes were identified on MRC in 5/62 patients (8%), including one PCC upgrade (II to III) and four partial/complete resolution of pseudotumoral cavernomas. 14/62 (23%) patients reported biliary interval symptoms, associated with long or severe CBD strictures.

**Conclusion:** Biliary symptoms are uncommon in patients with PCC. They mainly occur in patients with severe common bile duct strictures associated with grade III PCC. Nevertheless, 40% of these high-risk patients remain asymptomatic.

### SS 12.6

### Predication of primary sclerosing cholangitis complication using hepatobiliary phase gadoxetic acid-enhanced MRI

A. Kristic[1], S. Poetter-Lang[1], A. Messner[2], N. Bastati-Huber[1], R. Ambros[1], M. Trauner[1], A. Ba-Ssalamah[1]; *[1]Vienna/AT, [2]Wien/AT*

**Purpose:** Our goal was to assess clinical and prognostic value of T1-MRC (PFS) versus T2-MRCP dominant (DS) or high-grade (HGS) diagnoses in PSC patients.

**Material and Methods:** Six independent readers blinded to patient data reviewed 129 MRIs to diagnose DS and HGS on T2-MRCP using imaging guidelines. On T1-MRC, PFS was diagnosed if no GA excretion was seen in the common bile duct, the common hepatic duct, or the distal right or left hepatic ducts on the 20-minute HBP, i.e., impaired excretion. If 20-minute excretion was normal, patients were considered to have "no functional stricture" (NFS). T1-MRC diagnoses (NFS=87; PFS=42) were correlated with ERCP, clinical scores, labs, splenic volume, and clinical events. Statistical analyses included Kaplan–Meier curves and Cox regression.

**Results:** Interobserver agreement was excellent for NFS vs. PFS diagnosis on T1-MRC, but fair to moderate for DS and HGS on T2-MRCP. Labs and clinical scores were significantly worse for PFS vs. NFS. PFS patients underwent more diagnostic and therapeutic ERCPs, experienced more clinical events, and reached significantly more endpoints (p<0.001) than those with NFS. Multivariate analysis identified PFS as an independent risk factor for liver-related events.

**Conclusion:** T1-MRC was superior to T2-MRCP for stricture diagnosis and location. PFS correlated significantly with laboratory tests, clinical scores, and adverse events. PSC-strictures, either dominant (DS) or high-grade (HGS), can have an important prognostic impact. However, diagnostic interpretation, based upon T2-MRCP, remains controversial due to high inter-observer variability. We introduce the term "potential functional stricture" (PFS) on T1-weighted-hepatobiliary-phase (HBP) images of gadoxetic acid (GA)-enhanced-MR-cholangiography (T1-MRC).

### SS 12.7

### T1 and T2 parametric mapping to characterize patients with primary sclerosing cholangitis using non-invasive liver MRI

V.C. Obmann, S. Erb, D. Catucci, M. Ardoino, L. Ebner, J. Heverhagen, A. Christe, A.T. Huber; *Bern/CH*

**Purpose:** To analyze the predictive value of MRI T1 and T2 parametric mapping in patients with primary sclerosing cholangitis, as compared to matched healthy controls.

**Material and Methods:** For this IRB-approved study, we retrospectively included all patients with primary sclerosing cholangitis and a multiparametric MRI (1.5T and 3T), including parametric T1 and T2 sequences of the liver between 09/2018 and 05/2020 in our institution. Sex, age and field strength matched healthy controls with multiparametric MRI from our database based on no focal or diffuse liver disease in the clinical records or on the MRI images and a normal proton density fat fraction of the liver <10%. Non-contrast-enhanced relaxation times as well as extracellular volume fraction (ECV) were measured and compared between patients and controls using a Mann–Whitney U test.

**Results:** A total of 57 patients and their matched controls were analyzed (21 females and 36 males). Median age was 50 years (interquartile range 36–61 years). A median T1 and T2 relaxation time of the liver of 623 ms [IQR 577–657 ms] and 53 ms [51–57 ms] was measured in the primary sclerosing cholangitis group, compared to 583 ms [542–642 ms] and 50 ms [47–54 ms] in the control group (p=.15 and p=.004, respectively]. ECV was 37.2% [33–47%] in PSC patients and 47% [36–54%] in controls, p=.01.

**Conclusion:** MRI T1 and T2 parametric mapping is increased and ECV is decreased in patients with primary sclerosing cholangitis, as compared to age- and sex-matched healthy controls.

### SS 12.8

### Functional liver imaging score in patients with primary sclerosing cholangitis and different model for end-stage liver disease score

C. Dal Magro, A. Micelli, F. Crimì, E. Quaia, F. Vernuccio; *Padua/IT*

**Purpose:** The aim of our study was to assess the correlation between the functional liver imaging score (FLIS) on gadoxetate-disodium-enhanced MRI exam and model for end-stage liver disease score (MELD) in PSC patients with different degrees of liver dysfunction.

**Material and Methods:** We retrospectively included all patients with PSC, at least 1 gadoxetate-disodium-enhanced MRI exams, and available MELD score within 6 months from MRI. A radiologist calculated FLIS at each MRI available. Low FLIS was determined if the value was 3 or less. MELD score was distinguished in low (6 to 10) and high (>11). Categorical variables were compared using the Pearson $\chi^2$ or Fisher's exact test, as appropriate. A p value < 0.05 was considered significant. We estimated the regression between FLIS and MELD score.

**Results:** Twenty-five MRI exams were analyzed in 14 patients (mean age [SD]: 45 [11] years). FLIS was low in 7(28%) of 25 exams. MELD score was high at 11(44%) of 25 MRI exams. A score of 0 or 1 was assigned to liver parenchymal enhancement in 14/25[56%] exams, portal vein sign in 7/25[28%] and to the biliary contrast excretion in 2/25[8%]. The FLIS was inversely related (r = −0.77) with MELD score. Low FLIS was significantly associated with high MELD score (p= 0.0005).

**Conclusion:** In PSC patients, low FLIS occurs in about one third of MRI exams, mainly due to reduced liver enhancement compared to kidney. FLIS presents a strong negative correlation with the MELD score.

### *SS 12.10*

### Image findings and prognostic factors of incidentally detected gallbladder cancer

J. An, J. Kim, H. Kim, J. Choi*; Seoul/KR*

**Purpose:** To evaluate the image findings and prognostic factors of incidentally detected gallbladder cancer (IGBC) in comparison with benign GB diseases.

**Material and Methods:** Patients with surgically proven IGBC (n=85) and benign GB diseases (n=100) were retrospectively enrolled. All had contrast-enhanced CT before the surgery and patients with IGBC underwent serial CT follow-up after surgery. 82 patients had US. Two radiologists independently assessed the image findings suggestive of IGBC. They also analyzed clinical and CT findings suggestive of tumor recurrence after surgery. Univariate and multivariate analyses were performed to identify significant predictors for IGBC and recurrence.

**Results:** IGBC showed different image findings comparison with benign GB disease. In multivariate analysis, maximum GB wall thickness (OR, 1.31; 95% CI, 1.11–1.55; p=0.002) and enhancement pattern of GB wall (OR, 7.78; 95% CI, 2.84–21.39; p<0.001) were significant predictors of IGBC on CT. On US, mucosal disruption (OR, 26.29; 95% CI, 2.66–259.42; p=0.005) was a significant predictor of IGBC. 22 patients showed tumor recurrence during follow-up after surgery. For clinical factors, T-stage and N-stage were associated with recurrence (p<0.001). For image findings, only diffuse wall thickening (OR, 8.74; 95% CI, 1.13–67.49, p=0.038) on US was a significant predictor of recurrence in multivariate analysis.

**Conclusion:** Careful preoperative image interpretation and suspicion are essential to detect IGBC. Image findings can be useful to predict IGBC using imaging features such as maximum wall thickness, enhancement pattern of GB wall on CT, and mucosal disruption on US. In addition, diffuse wall thickening on US was useful to predict tumor recurrence.

### A

Aalbers A.: SS 1.8, SS 1.9
Agnello F.: SS 4.5, SS 4.8
Agostini A.: SS 10.8
Aguilers V.: SS 4.2
Ahmed F.: SS 2.5
Alberich Bayarri A.: SS 4.2, SS 11.1
Alderson S.: SS 10.2
Aleman Y.: SS 5.1
Alenzi T.: SS 6.11
Alfaro Cervello A.: SS 4.2
Ambros R.: SS 12.6
An J.: SS 3.3, SS 12.10
Andersen R.D.: SS 1.5
Andrasina T.: SS 2.6
Aonuma T.: SS 3.7
Ardoino M.: SS 12.7
Arrivi G.: SS 11.3
Aslan S.: SS 12.3
Atasever A.: SS 4.9
Atkinson D.: SS 6.9
Aubé C.: SS 10.10
Autelitano D.: SS 3.1, SS 4.4
Aydin E.: SS 10.5

### B

Bade R.: SS 4.10
Bae H.: SS 6.4
Bae J.: SS 10.1
Bakers F.: SS 10.3
Balduzzi A.: SS 8.8
Ballantyne S.: SS 6.6
Bannone E.: SS 8.7
Barber J.: SS 6.6
Bardhi E.: SS 3.1
Bariani M.: SS 8.2, SS 8.9, SS 11.8
Bartolotta T.: SS 4.5, SS 12.1
Bassaganyas Vancells C.: SS 8.6, SS 8.10
Ba-Ssalamah A.: SS 12.6
Bastati-Huber N.: SS 12.6
Baudouin M.: SS 7.5
Bauza M.: SS 4.2
Beable R.: SS 6.1
Beaufrère A.: SS 7.1, SS 7.6
Becker B.: SS 6.11
Bedossa P.: SS 4.1
Beek K.J.: SS 6.7, SS 6.10
Beets G.L.: SS 10.4
Beets-Tan R.: SS 1.8, SS 1.9, SS 10.3, SS 10.9
Belardy B.: SS 8.5
Bellot P.: SS 9.2
Belmonte E.: SS 4.3
Benlloch S.: SS 4.2
Benson S.: SS 10.9
Berardi E.: SS 1.8
Bereska J.: SS 3.6, SS 8.8
Besler M.: SS 2.7
Besselink M.: SS 3.6
Bessisow T.: SS 6.11
Betts M.: SS 6.1
Bhatnagar G.: SS 6.1, SS 6.6, SS 6.9
Bilhim T.: SS 3.10
Billingsley S.: SS 2.8
Bilreiro C.: SS 3.10
Bin S.: SS 5.5, SS 7.8
Bizeul J.: SS 5.6
Blad W.: SS 6.5, SS 6.8
Bloom S.: SS 6.8
Boffa E.: SS 8.2, SS 8.4
Bonvin M.: SS 5.1
Boone D.: SS 7.10
Borgheresi A.: SS 10.8
Borzi M.: SS 8.2, SS 8.9
Bouattour M.: SS 2.4, SS 7.1, SS 7.2
Boumalouka W.: SS 10.10
Bouvier A.: SS 9.9
Bradley D.: SS 2.8
Brancatelli G.: SS 4.8, SS 5.9
Braren R.: SS 3.2
Brönnimann M.: SS 9.4
Brotto M.: SS 8.9
Brown P.: SS 10.2
Bruining D.: SS 6.11
Buffart T.: SS 10.9
Bureau C.: SS 12.5
Buskens C.: SS 6.7, SS 6.10

### C

Cabibi D.: SS 4.5
Cairns J.: SS 2.8
Calame P.: SS 9.9
Cannella R.: SS 4.5, SS 4.8, SS 5.9, SS 7.1, SS 7.2, SS 7.6, SS 12.1
Canniff E.: SS 5.1
Cantasdemir M.: SS 2.3
Capozzi N.: SS 6.2
Cardobi N.: SS 3.1
Carli A.: SS 3.1
Caruso D.: SS 1.10, SS 4.7, SS 11.3, SS 12.2
Castera L.: SS 4.1
Castera L.: SS 9.1
Catucci D.: SS 9.4, SS 12.7
Cauchy F.: SS 7.1, SS 7.5, SS 7.6
Cerda Alberich L.: SS 4.2, SS 11.1
Cerocchi O.: SS 5.3
Chapellier P.: SS 11.2
Che F.: SS 5.5, SS 7.8
Cheon J.: SS 6.4
Chicha Cattoir V.: SS 7.2
Choi J.: SS 2.2, SS 3.3
Choi J.-I.: SS 5.8
Choi S.: SS 5.10
Choi J.: SS 12.10
Christe A.: SS 9.4, SS 12.7
Chung H.-J.: SS 4.6, SS 5.2
Cicalò F.: SS 11.8
Codjia T.: SS 8.3
Coello E.: SS 4.2
Correas J.-M.: SS 4.1
Corso R.: SS 1.4, SS 1.6
Costa L.: SS 8.7
Costa Andrade L.: SS 3.10
Coulibaly A.: SS 8.5
Crespo A.: SS 4.2
Crimì F.: SS 12.8

### D

Daams F.: SS 8.8
Dal Magro C.: SS 12.8
d'Alteroche L.: SS 9.9
Damion J.: SS 8.5
Darnell A.: SS 4.3, SS 8.6, SS 8.10
de Jonge C.: SS 6.7
de Mestier L.: SS 8.1, SS 8.3
De Robertis R.: SS 3.1, SS 4.4, SS 8.8
De Santis D.: SS 1.10, SS 12.2
de Voogd F.: SS 6.7
Del Amo F.: SS 9.2
Del Gaudio A.: SS 4.7, SS 12.2
Delabrousse E.: SS 9.9
Demir E.I.: SS 3.2
D'Haens G.: SS 6.7, SS 6.10
Dijkhoff R.: SS 10.3
Dioguardi Burgio M.: SS 2.4, SS 4.1, SS 7.1, SS 7.2, SS 7.6, SS 8.1, SS 8.3, SS 9.1
Djuric - Stefanovic A.: SS 11.4
Dokmak S.: SS 8.1, SS 8.3, SS 12.5
D'Onofrio M.: SS 3.1, SS 4.4
Douane F.: SS 9.9
Drago S.: SS 10.3
Dromain C.: SS 5.1, SS 11.2
Drucker Iarovich M.: SS 11.9
Du Boisbaudry C.: SS 7.2
Du Pasquier C.: SS 5.1
Dubois M.: SS 5.1
Dumitru R.L.: SS 2.1
Duncan G.: SS 6.1
Dupuis M.: SS 2.4

Scientific Sessions

Authors' Index

### E

Ebner L.: SS 9.4, SS 12.7
Ebrahimi K.: SS 11.4
El Homsi M.: SS 2.5
El-Karim L.A.: SS 5.3
Emmanuel E.A.: SS 6.8
Erb S.: SS 12.7
Ergin B.: SS 10.5
Escudero Garcia D.: SS 4.2

### F

Farhang Zangeneh H.: SS 5.3
Fasquelle F.: SS 11.2
Fatima-Zohra M.: SS 9.9
Faure A.: SS 7.1
Feld J.: SS 5.3
Fernandez Paton M.: SS 4.2
Fernàndez-Clotet A.: SS 6.2
Fernández-Moscoso A.: SS 9.2
Ferrández D.: SS 9.2
Ferrer-Fabrega J.: SS 8.6, SS 8.10
Fijneman P.: SS 1.9
Fitzke H.: SS 6.5, SS 6.6, SS 6.8, SS 6.9
Foley K.: SS 1.2
Fortuna L.: SS 8.7
Fraga M.: SS 5.1
Frederic P.: SS 12.5
Fujinaga Y.: SS 3.7
Fukuzawa T.: SS 3.7

### G

Garcia C.: SS 4.3
Garcia Criado M.: SS 4.3
Garcia-Criado A.: SS 8.6, SS 8.10
Garteiser P.: SS 4.1, SS 9.1
Garzelli L.: SS 2.4
Gecse K.: SS 6.7, SS 6.10
Gelfi J.: SS 9.9
Gence-Breney A.: SS 8.1
Geraci L.: SS 3.1
Geubels B.M.: SS 10.4
Giandola T.: SS 1.4, SS 1.6
Giovagnoni A.: SS 10.8
Girardet R.: SS 5.1
Glover T.: SS 6.5
Gokhan M.: SS 2.7
Gomis Maya A.: SS 11.1
Gonzalez-Montpetit E.: SS 9.1
Gorini G.: SS 1.1
Gorycki T.: SS 2.9
Goujon J.: SS 8.5
Goupil J.: SS 9.9
Grasu M.C.: SS 2.1
Grawe F.: SS 11.10
Greenhalgh R.: SS 6.6
Greget M.: SS 9.9
Gregory J.: SS 2.4
Grotenhuis B.A.: SS 10.4
Gu K.: SS 7.3
Guagenti D.: SS 4.4
Gulizia M.: SS 5.1
Gupta A.: SS 6.1

### H

Haefliger L.: SS 11.2
Hajj C.: SS 2.5
Halligan S.: SS 6.1, SS 7.10
Hamdan D.: SS 2.5
Hameed M.: SS 6.5, SS 7.10
Hansen B.: SS 5.3
Harder F.N.: SS 3.2
Herpe G.: SS 8.5
Herraiz I.: SS 9.2
Hesseldal L.: SS 1.5
Heverhagen J.: SS 12.7
Higginson A.: SS 6.1, SS 6.6
Hinzpeter R.: SS 11.9
Hisoğlu F.: SS 10.5
Hobeika C.: SS 7.5
Holmes J.: SS 6.5, SS 6.6, SS 6.8, SS 6.9
Horsthuis K.: SS 6.10
Horvat N.: SS 2.5
Huber A.: SS 9.4, SS 12.7
Hueniken K.: SS 11.9
Hulbert L.A.: SS 9.4
Hur B.: SS 10.1
Hyland R.: SS 6.1, SS 6.6

### I

Ichinohe F.: SS 3.7
Inan Gurcan N.: SS 2.3, SS 4.9
Incarbone G.: SS 4.4
Inci E.: SS 3.4
Infantino G.: SS 4.5
Ingwersen E.: SS 8.8
Ippolito D.: SS 1.4, SS 1.6
Iuhas N.G.: SS 2.1

### J

Jairath V.: SS 6.11
Jang H.: SS 5.10
Jankovic A.: SS 11.4
Janssen H.: SS 5.3
Janssen B.: SS 8.8
Jeon S.: SS 10.1
Jimenez Pastor A.: SS 4.2, SS 11.1
Jong-Min K.: SS 4.6
Joo I.: SS 5.8
Jreige M.: SS 5.1, SS 11.2
Jung E.: SS 3.2

### K

Kaiser J.: SS 3.5
Kaiser N.: SS 4.10
Karampinos D.C.: SS 3.2
Kauczor H.-U.: SS 3.5
Kaya T.: SS 10.5
Khalili K.: SS 5.3
Kim B.: SS 12.4
Kim H.: SS 3.3, SS 11.7, SS 12.10
Kim H.: SS 10.6
Kim J.: SS 2.2
Kim J.: SS 3.3, SS 12.10
Kim J.: SS 12.4
Kim J.-M.: SS 5.2
Kim K.: SS 2.2
Kim S.: SS 10.1, SS 11.7
Kim S.: SS 10.6
Kim S.: SS 5.8
Kim S.-S.: SS 6.4
Kim Y.: SS 9.3
Kini R.: SS 11.2
Klauß M.: SS 3.5
Klompenhouwer E.: SS 10.9
Koc U.: SS 2.7
Kok N.: SS 1.8, SS 1.9
Kop M.: SS 3.6
Kovac J.: SS 11.4
Kristic A.: SS 12.6
Kumar S.: SS 6.1

### L

Laghi A.: SS 1.10, SS 4.7, SS 11.3, SS 12.2
Lahaye M.: SS 1.8, SS 1.9
Lambie H.: SS 6.6
Lambregts D.: SS 1.8, SS 1.9, SS 10.3
Lapham R.: SS 6.1
Lazic L.: SS 11.4
Lebtahi R.: SS 2.4
Lee C.-H.: SS 5.8
Lee D.: SS 5.2
Lee E.: SS 5.7
Lee H.: SS 11.7
Lee H.: SS 12.4
Lee J.: SS 2.2
Lee J.: SS 4.6
Lee J.-H.: SS 5.2
Lee L.: SS 1.2
Lee Y.: SS 6.3

Lee Y.: SS 9.3
Lim J.: SS 6.4
Liu X.-J.: SS 11.6
Loffroy R.: SS 9.9
Lung P.: SS 6.6
Lupescu I.G.: SS 2.1

### M

Maas M.: SS 10.3, SS 10.4, SS 10.9
Macdonald B.: SS 6.11
Maino C.: SS 1.4, SS 1.6
Mainta E.: SS 6.6
Makowski M.R.: SS 3.2
Malakzadeh S.: SS 10.10
Mallardi B.: SS 1.1
Malleo G.: SS 8.2
Mallett S.: SS 6.1, SS 6.6, SS 7.10
Mallick R.: SS 6.11
Manasseh G.: SS 5.1
Manceau H.: SS 7.2
Mansueto G.: SS 8.2, SS 8.4, SS 8.9, SS 8.7, SS 11.8
Mantellini P.: SS 1.1
Mantziari S.: SS 11.2
Marchegiani G.: SS 8.7, SS 8.8
Marchegiani M.: SS 10.8
Mariño Z.: SS 4.3
Maris B.: SS 8.7
Marquering H.: SS 3.6
Marti Aguado D.: SS 4.2
Marti Bonmati L.: SS 4.2, SS 11.1
Mascalchi M.: SS 1.1
Masci B.: SS 4.7
Masulovic D.: SS 11.4
Matkulcik P.: SS 2.6
Matos C.: SS 3.10, SS 7.6
Matteini F.: SS 10.10
Mayer P.: SS 3.5
Mazzola M.: SS 12.1
Mazzuca F.: SS 11.3
McCurdy J.: SS 6.11
Mclernon D.: SS 10.7
Menys A.: SS 6.8, SS 6.9
Meserve J.: SS 6.11
Messner A.: SS 12.6
Metser U.: SS 11.9
Micelli A.: SS 12.8
Milazzo M.: SS 4.8, SS 5.9
Miller C.: SS 6.8
Milosevic S.: SS 11.4
Min J.: SS 7.3
Mitchell-Hay R.: SS 10.7
Mitrovic M.: SS 11.4
Moloney B.: SS 11.9
Monraats M.: SS 10.9
Montecchiesi E.: SS 10.8
Moreno J.: SS 4.3
Mulders L.: SS 6.10
Munir J.: SS 6.11
Murray A.: SS 10.7
Muthoo C.: SS 10.2

### N

Nalbant M.: SS 3.4
Nam Y.: SS 5.8
Nam K.: SS 10.6
Nardacci S.: SS 11.3
Nederveen A.J.: SS 3.8, SS 3.9
Nio Y.: SS 3.6, SS 8.8
Nonaka T.: SS 3.7
Nowak K.: SS 2.9

### O

Obmann V.: SS 9.4, SS 12.7
O'Brien H.: SS 10.7
Olivieri A.: SS 4.4
Onder R.O.: SS 12.3
Ordas I.: SS 6.2
Ortega C.: SS 11.9
Ottaviani L.: SS 10.8
Oufighou M.: SS 4.1
Oyama K.: SS 3.7
Ozdemir M.: SS 2.7

### P

Pagé G.: SS 9.1
Paisant A.: SS 10.10
Pakpoor J.: SS 6.5, SS 6.8, SS 6.9
Palazón Á.: SS 9.2
Panes J.: SS 6.2
Papadopoulos P.: SS 9.9
Papanikolaou N.: SS 7.6
Paradis V.: SS 4.1, SS 7.2, SS 9.1
Park B.: SS 2.2
Park B.: SS 5.8
Park E.: SS 6.3
Park H.: SS 5.8
Park J.: SS 5.7
Park S.: SS 4.6, SS 5.2
Park Y.: SS 5.7
Parlow S.: SS 6.11
Parry T.: SS 6.1, SS 6.6
Patel A.: SS 6.6
Patel N.: SS 6.6
Patel U.: SS 6.1
Payance A.: SS 7.2, SS 12.5
Pedersen M.: SS 1.5
Peoch K.: SS 7.2
Perez C.: SS 4.3
Perez Girbes A.: SS 4.2
Perez Serrano C.: SS 8.6, SS 8.10
Petta S.: SS 4.5
Phillip V.: SS 3.2
Pilato G.: SS 12.1
Pilcher J.: SS 6.1
Platt J.: SS 1.7
Platz J.: SS 4.10
Plessier A.: SS 12.5
Plumb A.: SS 1.3, SS 6.1
Poetter-Lang S.: SS 12.6
Polici M.: SS 1.10
Polidori T.: SS 4.7
Porrello G.: SS 4.5
Puglia V.: SS 4.2
Pugliese D.: SS 12.2
Puliti D.: SS 1.1

### Q

Quaia E.: SS 12.8
Quirke P.: SS 10.2

### R

Rafaelsen S.: SS 1.5
Rafart Martinez G.: SS 8.6
Rafiee H.: SS 6.6
Ragusi M.: SS 1.4, SS 1.6
Ramsay T.: SS 6.11
Rancatore G.: SS 4.8
Rautou P.-E.: SS 4.1, SS 12.5
Regent B.: SS 2.9
Reid J.: SS 6.11
Reizine E.: SS 12.5
Ren L.: SS 9.8
Ricart E.: SS 6.2
Rijsemus C.: SS 1.8, SS 1.9
Rimola J.: SS 4.3, SS 6.2
Rio Bartulos C.: SS 4.10
Risson J.-R.: SS 9.9
Robinson C.: SS 6.6
Rodes A.: SS 9.9
Rohan T.: SS 2.6
Ronot M.: SS 2.4, SS 4.1, SS 7.1, SS 7.2, SS 7.5, SS 7.6, SS 8.1, SS 8.3, SS 9.1, SS 10.10, SS 12.5
Rosenfeld G.: SS 6.11
Rossi R.: SS 10.8
Rossington H.: SS 10.2
Runge J.H.: SS 3.8, SS 3.9

### S

Saglietti C.: SS 11.2
Sahin T.: SS 4.9
Sahin S.: SS 10.5
Sali L.: SS 1.1
Sanchez Martin A.: SS 4.2
Sapena V.: SS 8.10
Saponjski D.: SS 11.4
Sartoris R.: SS 4.1, SS 7.1, SS 7.5, SS 8.1, SS 8.3, SS 10.10
Satinha J.: SS 7.6
Sauvanet A.: SS 8.1, SS 8.3
Schrauben E.M.: SS 3.8, SS 3.9
Schumacher M.: SS 4.10
Seligmann J.: SS 1.7
Senk K.: SS 4.10
Seo G.: SS 5.7
Seo N.: SS 6.4
Server S.: SS 2.3
Sever I.: SS 4.9
Sever S.: SS 4.9
Singh S.: SS 6.11
Skrobic O.: SS 11.4
Slater A.: SS 6.1
Snaebjornsson P.: SS 1.9
Soh E.: SS 9.3
Soler A.: SS 4.3
Soler J.: SS 8.6
Soler-Perromat J.: SS 8.10
Soler-Perromat A.: SS 8.10
Son J.-H.: SS 6.3
Song B.: SS 9.8, SS 9.10
Sorrentino F.: SS 5.9
Spezia A.: SS 8.4
Spinello A.: SS 4.5
Spoto F.: SS 4.4
Stephenson J.: SS 1.2
Stoker J.: SS 3.6, SS 3.8, SS 3.9, SS 6.7, SS 6.10, SS 8.8
Stošić K.: SS 11.4
Struik F.: SS 3.6, SS 8.8
Suh J.: SS 10.1
Sulun E.: SS 12.3
Sung J.: SS 5.7
Sutter O.: SS 9.9
Sznitman R.: SS 9.4
Szurowska E.: SS 2.9

### T

Taibbi A.: SS 4.5, SS 12.1
Talei Franzesi C.: SS 1.4, SS 1.6
Taouli B.: SS 5.1
Tasdemir M.N.: SS 12.3
Tasu J.-P.: SS 8.5
Taylor S.: SS 1.3, SS 6.1, SS 6.5, SS 6.6, SS 6.8
Ten Esteve A.: SS 4.2
Tessier F.: SS 8.3
Tielbeek J.: SS 6.10
Todesco M.: SS 3.1
Tolan D.: SS 1.2, SS 1.3, SS 1.7, SS 2.8, SS 6.1, SS 6.6, SS 10.2
Toma M.: SS 2.1
Tomaiuolo L.: SS 3.1
Tomelleri G.: SS 11.8
Tougeron D.: SS 8.5
Trauner M.: SS 12.6
Tse J.R.: SS 7.9

### U

Ulusoy O.: SS 2.3, SS 4.9
Upponi S.: SS 6.6
Ustun M.: SS 10.5

### V

Valanzuolo D.: SS 1.10, SS 12.2
Valek V.: SS 2.6
Valla D.: SS 9.1
Van Beers B.: SS 4.1, SS 9.1
Van der Reijd D.: SS 10.3, SS 10.9
Van Dijk E.: SS 10.9
van Griethuysen J.: SS 10.3
van Laarhoven H.W.: SS 3.8, SS 3.9
Van Rijn K.: SS 6.7, SS 6.10
van Schelt A.-S.: SS 3.8, SS 3.9
Veiga-Canuto D.: SS 11.1
Veit-Haibach P.: SS 11.9
Velasco S.: SS 8.5
Ventura-Aguiar P.: SS 8.10
Vernuccio F.: SS 12.8
Verpalen I.: SS 3.6, SS 8.8
Vietti Violi N.: SS 5.1, SS 11.2
Vilgrain V.: SS 2.4, SS 4.1, SS 7.1, SS 7.2, SS 7.5, SS 7.6, SS 8.1, SS 8.3, SS 9.1, SS 10.10, SS 12.5
Vionnet M.: SS 8.5
Virzi A.: SS 10.10
Vivarelli M.: SS 10.8

### W

Walklett C.: SS 2.8
Wan S.: SS 9.8, SS 9.10
Wassenaar N.P.: SS 3.8, SS 3.9
Wei Y.: SS 9.8
Weiss K.: SS 3.2
Wen J.T.: SS 7.9
Westerink B.: SS 10.9
Whitley L.: SS 6.5
Wiggermann P.: SS 4.10
Wild C.: SS 3.5
Woetzel J.: SS 4.10
Wu Y.: SS 1.2

### Y

Yang C.: SS 7.7, SS 11.5, SS 11.6
Yanofsky R.: SS 6.11
Yazici S.: SS 4.9
Yeung J.: SS 7.10
Yoon J.: SS 4.6
Yoon J.-H.: SS 6.3
Youn S.: SS 5.8
Yuzer Y.: SS 4.9

### Z

Zamboni G.A.: SS 8.2, SS 8.4, SS 8.7, SS 8.9, SS 11.8
Zanutto P.: SS 4.4
Zappa M.: SS 1.1
Zarate-Lopez N.: SS 6.8
Zavadil J.: SS 2.6
Zbinden L.: SS 9.4
Zealley I.: SS 6.1, SS 6.6
Zekrini K.: SS 12.5
Zerunian M.: SS 1.10, SS 4.7, SS 11.3
Zhang X.: SS 9.10
